# Estimating the Incidence and Key Risk Factors of Cardiovascular Disease in Patients at High Risk of Imminent Fracture Using Routinely Collected Real‐World Data From the UK

**DOI:** 10.1002/jbmr.4648

**Published:** 2022-09-08

**Authors:** Marta Pineda‐Moncusí, Leena El‐Hussein, Antonella Delmestri, Cyrus Cooper, Alireza Moayyeri, Cesar Libanati, Emese Toth, Daniel Prieto‐Alhambra, Sara Khalid

**Affiliations:** ^1^ Centre for Statistics in Medicine, Nuffield Department of Orthopaedics, Rheumatology and Musculoskeletal Sciences, Nuffield Orthopaedic Centre University of Oxford Oxford UK; ^2^ MRC Lifecourse Epidemiology Unit University of Southampton, Southampton General Hospital Southampton UK; ^3^ UCB Biopharma Sprl Brussels Belgium; ^4^ Fundació Institut Universitari per a la recerca a l'Atenció Primària de Salut Jordi Gol i Gorina (IDIAPJ Gol) CIBERFES Barcelona Spain; ^5^ Universitat Autònoma de Barcelona Bellaterra (Cerdanyola del Vallès) Cerdanyola del Vallès Spain

**Keywords:** OSTEOPOROSIS, ORAL BISPHONATES, CARDIOVASCULAR RISK ASSESSMENT, INCIDENCE RATES, PROGNOSTIC MODEL

## Abstract

The objective of this work was to estimate the incidence rate of cardiovascular disease (CVD) events (myocardial infarction, stroke, or CVD death) at 1 year among three cohorts of patients at high risk of fracture (osteoporosis, previous fracture, and anti‐osteoporosis medication) and to identify the key risk factors of CVD events in these three cohorts. To do so, this prospective cohort study used data from the Clinical Practice Research Datalink, a primary care database from United Kingdom. Major adverse cardiovascular events (MACE, a composite outcome for the occurrence of either myocardial infarction [MI], stroke, or CVD death) were identified in patients aged 50 years or older at high or imminent fracture risk identified in three different cohorts (not mutually exclusive): recently diagnosed with osteoporosis (OST, *n* = 65,295), incident fragility fracture (IFX, *n* = 67,065), and starting oral bisphosphonates (OBP, *n* = 145,959). About 1.90%, 4.39%, and 2.38% of the participants in OST, IFX, and OBP cohorts, respectively, experienced MACE events. IFX was the cohort with the higher risk: MACE incidence rates (cases/1000 person‐years) were 19.63 (18.54–20.73) in OST, 52.64 (50.7–54.5) in IFX, and 26.26 (25.41–27.12) in OBP cohorts. Risk of MACE events at 1 year was predicted in the three cohorts. Models using a set of general, CVD, and fracture candidates selected by lasso regression had a good discrimination (≥70%) and internal validity and generally outperformed the models using only the CVD risk factors of general population listed in QRISK tool. Main risk factors common in all MACE models were sex, age, smoking, alcohol, atrial fibrillation, antihypertensive medication, prior MI/stroke, established CVD, glomerular filtration rate, systolic blood pressure, cholesterol levels, and number of concomitant medicines. Identified key risk factors highlight the differences of patients at high risk of fracture versus general population. Proposed models could improve prediction of CVD events in patients with osteoporosis in primary care settings. © 2022 The Authors. *Journal of Bone and Mineral Research* published by Wiley Periodicals LLC on behalf of American Society for Bone and Mineral Research (ASBMR).

## Graphical abstract

Among individuals at high risk of imminent fractures, those with incident fractures were at highest risk. A cardiovascular risk prediction tool was developed specifically for those at high risk of imminent fractures. The proposed tool outperforms existing models used in current clinical practice.

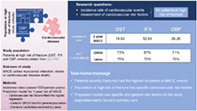

## Introduction

Cardiovascular disease (CVD) and osteoporosis are both leading causes of morbidity and mortality^(^ ^1^, ^2^ ^)^ worldwide and their prevalence increases with age.^(^ ^3^, ^4^ ^)^ Several prediction tools have been developed for cardiovascular events (heart attacks or stroke), including the Framingham Heart Study,^(^ ^5^, ^6^, ^7^ ^)^ CHADS2 tool,^(^ ^8^, ^9^, ^10^ ^)^ and QRISK tool.^(^ ^11^, ^12^ ^)^ These tools have been developed in general, usually younger, populations and have not been validated in patients with osteoporosis.

Identification of CVD risk factors is particularly challenging for patients with osteoporosis because the association between fracture risk or anti‐osteoporosis treatment and cardiovascular events remains unclear.^(^ ^13^ ^)^ Some established risk factors for osteoporosis and fractures,^(^ ^14^ ^)^ such as female sex and low weight, have been found to be protective against CVD. Conversely, some other risk factors, including age, low bone mineral density,^(^ ^15^ ^)^ prior fracture, obesity,^(^ ^16^, ^17^, ^18^ ^)^ or type 2 diabetes,^(^ ^19^ ^)^ are associated with an increased risk of CVD. Patients with a history of CVD have been shown to be at increased risk of osteoporotic fractures,^(^ ^20^ ^)^ whereas higher risk of stroke and coronary artery disease is observed among patients with osteoporotic fracture or low bone mineral density.^(^ ^21^ ^)^

The effects of anti‐osteoporosis medications on CVD risk is inconclusive: despite no evidence from clinical trials that oral bisphosphonates (BP) have an impact on cardiovascular risk,^(^ ^22^, ^23^ ^)^ some publications suggest a protective effect.^(^ ^24^ ^)^ Meanwhile, the European Medicines Agency (EMA) has advised contraindications to patients with a history of prior myocardial infarction (MI) or stroke regarding romosozumab, the most recent medication option for osteoporosis,^(^ ^25^ ^)^ in addition to the preexisting restrictions to menopausal hormone therapy^(^ ^26^ ^)^ and strontium ranelate.^(^ ^27^ ^)^ This variable impact of anti‐osteoporotic medication on CVD risk highlights the clinical utility of identifying patients who are being considered for osteoporosis treatment and might be at elevated risk of CVD.

To address this issue, our overarching aim was to assess the absolute risk of CVD experienced by elderly patients at higher fracture risk in the UK, as well as to identify key CVD risk factors (both generic and specific ones) for these patients. We estimate incidence rates of major adverse cardiovascular events (MACE) among cohorts newly diagnosed with osteoporosis, those with first recorded fracture, and oral BP therapy initiators obtained from the UK general population, and developed and internally validated models that predict 1‐year MACE in these cohorts of high‐risk patients. Additionally, a secondary analysis of 2‐year MACE and a sensitivity analysis for MI/stroke prediction is reported.

## Subjects and Methods

### Data source

Data for this study were obtained from the Clinical Practice Research Datalink (CPRD) GOLD, which contains anonymized electronic primary care records for the UK (www.cprd.com/primarycare). In addition to demographic information, the data included medication prescriptions, clinical events, tests, referrals, and hospital admissions along with their major outcomes in a sample of >16 million patients (including deceased and transferred out; 2.3 million are current patients, covering approximately 3.6% of UK population).^(^ ^28^, ^29^ ^)^ For this study, an extract from January 1, 1995, to January 31, 2017, was used. We used CPRD GOLD data linked to the Hospital Episode Statistics (HES) Admitted Patient Care, which contains clinical diagnoses during hospital admissions in England, to the Office for National Statistics (ONS) mortality records, and to the Index of Multiple Deprivation (IMD) data set. We reconciled CPRD GOLD and ONS mortality dates of death following published guidelines.^(^ ^30^ ^)^

### Participants

The study population included patients at high or imminent fracture risk as identified from literature, divided into three cohorts:

1. Patients with an incident diagnosis of osteoporosis (read or ICD‐10 codes). We refer to this group as the osteoporosis cohort (OST).
2. Patients with a first incident fracture at an osteoporotic site (all except face, skull, and digits), diagnosed either through read codes or ICD‐10 codes. This cohort is referred to as the imminent fracture risk cohort (IFX).
3. Incident users of oral bisphosphonates (BP) without BP use in the prior year, referred to as the oral BP treatment cohort (OBP).

Index date was defined as time of recorded incident diagnosis, first incident fracture, and incident use of BP, for the OST, IFX, and OBP cohorts, respectively. Participants were followed from index date up to a maximum of 2 years. We censored participants at the earliest of first: study outcome, death, transfer out of practice, or the end of follow‐up period. Included patients were at least 50 years old and had at least 1 year of data available before index date. Participants could potentially be present in more than one of the cohorts above, with different index dates. For OBP cohort, subjects with use of any anti‐osteoporotic drug (except calcium and vitamin D supplements) in the previous year were excluded.

In the IFX cohort, high risk of imminent fracture was defined following Kanis and colleagues^(^ ^31^ ^)^ designation: imminent risk period is the following 2‐year period after a fracture.^(^ ^32^ ^)^

### Candidate risk factors

The overall set of variables considered for inclusion in the prediction model contained risk factors from the QRISK model,^(^ ^12^ ^)^ as well as additional risk factors identified in the literature as being potentially associated with CVD.^(^ ^15^, ^16^, ^17^, ^20^, ^21^, ^33^, ^34^, ^35^, ^36^, ^37^, ^38^, ^39^, ^40^, ^41^ ^)^ These included sociodemographic and lifestyle factors, laboratory measurements, medications, and comorbidities (Table 1).

**Table 1:** Baseline Characteristics of Patients in the Development Set and the Validation Set

Variable	OST	IFX	OBP
	*n* = 65,295	*n* = 67,065	*n* = 145,959
Sex, male (%)	8616 (13.2)	16,386 (24.4)	29,547 (20.2)
Age >75 years	31,105 (47.6)	48,726 (72.7)	77,199 (52.9)
Age group, years (%)			
50–59	8315 (12.7)	4764 (7.1)	16,388 (11.2)
60–69	15,949 (24.4)	7646 (11.4)	31,665 (21.7)
70–79	21,165 (32.4)	15,573 (23.2)	45,679 (31.3)
80–89	16,586 (25.4)	27,546 (41.1)	42,053 (28.8)
>89	3280 (5.0)	11,536 (17.2)	10,174 (7.0)
SES (%)			
1	15,953 (24.4)	14,980 (22.3)	35,837 (24.6)
2	15,643 (24.0)	15,990 (23.8)	35,738 (24.5)
3	13,794 (21.1)	14,322 (21.4)	30,877 (21.2)
4	11,921 (18.3)	12,892 (19.2)	26,566 (18.2)
5	7918 (12.1)	8820 (13.2)	16,828 (11.5)
Smoking^a^ (%)			
Ex	20,487 (31.4)	21,484 (32.0)	50,162 (34.4)
No	34,049 (52.1)	35,272 (52.6)	75,838 (52.0)
Yes	10,759 (16.5)	10,309 (15.4)	19,959 (13.7)
Drinking^a^ (%)			
Ex	3564 (5.5)	3945 (5.9)	7226 (5.0)
No	17,062 (26.1)	21,092 (31.5)	41,791 (28.6)
Yes	44,669 (68.4)	42,028 (62.7)	96,942 (66.4)
Diabetes type I^b^ (%)	152 (0.2)	164 (0.2)	297 (0.2)
Diabetes type II^b^ (%)	3311 (5.1)	4171 (6.2)	8340 (5.7)
Chronic obstructive pulmonary disease^b^ (%)	4939 (7.6)	3745 (5.6)	11251 (7.7)
Chronic kidney disease^b^ (%)	5790 (8.9)	8117 (12.1)	13396 (9.2)
Rheumatoid arthritis^b^ (%)	5045 (7.7)	2891 (4.3)	19746 (13.5)
Lupus^b^ (%)	144 (0.2)	55 (0.1)	328 (0.2)
Systemic heart disease^a^ (%)	2341 (3.6)	563 (0.8)	5222 (3.6)
Anti‐osteoporosis use^a^ (%)	11752 (18.0)	5356 (8.0)	
Heparin use^a^ (%)	372 (0.6)	282 (0.4)	1035 (0.7)
Beta‐blocker use^a^ (%)	10564 (16.2)	10392 (15.5)	22753 (15.6)
Hypertension^a^ (%)	5091 (7.8)	3334 (5.0)	9514 (6.5)
Deep vein thrombosis or pulmonary embolism^a^ (%)	522 (0.8)	439 (0.7)	1334 (0.9)
Anticoagulant use^a^ (%)	3285 (5.0)	3536 (5.3)	7546 (5.2)
Antidepressants TCA^a^ (%)	7039 (10.8)	5352 (8.0)	14,451 (9.9)
Antidepressants SSRI^a^ (%)	5990 (9.2)	7293 (10.9)	12651 (8.7)
Hypercholesterolemia^a^ (%)	1561 (2.4)	667 (1.0)	2543 (1.7)
Statin use^a^ (%)	15571 (23.8)	14084 (21.0)	33,988 (23.3)
Osteoporosis history^b^	NA	5521 (8.2)	46215 (31.7)
Family history of cardiovascular disease (%)	6755 (10.3)	4036 (6.0)	13204 (9.0)
Family history of cardiovascular disease before age 60 years (%)	99 (0.2)	47 (0.1)	170 (0.1)
Heart failure^b^ (%)	2323 (3.6)	3405 (5.1)	5040 (3.5)
Migraine^b^ (%)	10103 (15.5)	6294 (9.4)	19961 (13.7)
Severe mental illness^b^ (%)	10203 (15.6)	8836 (13.2)	19256 (13.2)
Vascular disease^b^ (%)	839 (1.3)	1078 (1.6)	1765 (1.2)
Atrial fibrillation^b^ (%)	3664 (5.6)	4784 (7.1)	8022 (5.5)
On antihypertensive drug (%)	37053 (56.7)	38036 (56.7)	79044 (54.2)
Antipsychotic use^a^ (%)	371 (0.6)	867 (1.3)	773 (0.5)
Steroid use^a^ (%)	9367 (14.3)	5181 (7.7)	37201 (25.5)
Erectile dysfunction^a^ (%)	871 (1.3)	1065 (1.6)	2797 (1.9)
Charlson score (%)			
0	37,782 (57.9)	40,024 (59.7)	80,717 (55.3)
1	13,274 (20.3)	11,209 (16.7)	31,132 (21.3)
2	7960 (12.2)	7388 (11.0)	18,141 (12.4)
≥3	6279 (9.6)	8444 (12.6)	15,969 (10.9)
Cardiovascular disease (%)			
No	58,034 (88.9)	58,381 (87.1)	128,516 (88.0)
Ever >1 year before index date	5616 (8.6)	6996 (10.4)	13,474 (9.2)
1 year before index	719 (1.1)	743 (1.1)	1551 (1.1)
6 months before index	723 (1.1)	767 (1.1)	1763 (1.2)
1 month before index	203 (0.3)	178 (0.3)	655 (0.4)
MI or stroke (%)			
No	61,350 (94.0)	60,046 (89.5)	135,335 (92.7)
Ever >1 year before index date	2850 (4.4)	5083 (7.6)	7481 (5.1)
1 year before index	1095 (1.7)	1936 (2.9)	3143 (2.2)
Established CVD^b^ = ever (%)	7428 (11.4)	11,506 (17.2)	18,774 (12.9)
Any fracture history (%)			
No	49,542 (75.9)	67,065 (100)	114,386 (78.4)
Ever >1 year before index date	5813 (8.9)	0	10,598 (7.3)
1 year before index	9940 (15.2)	0	20,975 (14.4)
Hip fracture history (%)			
No	62,040 (95.0)	67,065 (100)	136,610 (93.6)
Ever >1 year before index date	1118 (1.7)	0	1720 (1.2)
1 year before index	2137 (3.3)	0	7629 (5.2)
Shoulder fracture history (%)			
No	64,836 (99.3)	67,065 (100)	145,199 (99.5)
Ever >1 year before index date	232 (0.4)	0	333 (0.2)
1 year before index	227 (0.3)	0	427 (0.3)
Spine fracture history (%)			
No	63,880 (97.8)	67,065 (100)	143,554 (98.4)
Ever >1 year before index date	326 (0.5)	0	356 (0.2)
1 year before index	1089 (1.7)	0	2049 (1.4)
Wrist fracture history (%)			
No	60,237 (92.3)	67,065 (100)	137,118 (93.9)
Ever >1 year before index date	2363 (3.6)	0	4040 (2.8)
1 year before index	2695 (4.1)	0	4801 (3.3)
BMI^a^ (%)			
<18.5	5615 (8.6)	9265 (13.8)	10,877 (7.5)
18.6–24.9	32,564 (49.9)	33,554 (50.0)	66,542 (45.6)
25–29.9	18,343 (28.1)	16,720 (24.9)	44,109 (30.2)
30–39.9	8164 (12.5)	6983 (10.4)	22,452 (15.4)
≥40	609 (0.9)	543 (0.8)	1979 (1.4)
No. of GP visits^a^ (%)			
0	3219 (4.9)	15,017 (22.4)	15,177 (10.4)
1–5	16,176 (24.8)	15,528 (23.2)	36,213 (24.8)
6–10	17,452 (26.7)	13,677 (20.4)	32,344 (22.2)
11–15	11,938 (18.3)	9140 (13.6)	24,466 (16.8)
≥16	16,510 (25.3)	13,703 (20.4)	37,759 (25.9)
No. of GP emergency visits^a^ (%)			
0	53,095 (81.3)	53,221 (79.4)	119,698 (82.0)
1	6570 (10.1)	6392 (9.5)	14,083 (9.6)
2	2439 (3.7)	2844 (4.2)	5282 (3.6)
3–5	2282 (3.5)	3163 (4.7)	4993 (3.4)
≥6	909 (1.4)	1445 (2.2)	1903 (1.3)
eGFR^a^ (%)			
≤29	708 (1.1)	1974 (2.9)	1777 (1.2)
30–44	3470 (5.3)	7099 (10.6)	9749 (6.7)
45–59	11,950 (18.3)	15,335 (22.9)	30,290 (20.8)
60–89	45,497 (69.7)	39,774 (59.3)	96,838 (66.3)
≥90	3670 (5.6)	2883 (4.3)	7305 (5.0)
SBP^a^ (%)			
<120	9076 (13.9)	9184 (13.7)	18,549 (12.7)
120–139	26,605 (40.7)	25,872 (38.6)	57,685 (39.5)
140–159	22,588 (34.6)	23,213 (34.6)	51,589 (35.3)
≥160	7026 (10.8)	8796 (13.1)	18,136 (12.4)
DBP^a^ (%)			
<80	33,729 (51.7)	36,247 (54.0)	75,132 (51.5)
80–89	24,505 (37.5)	23,468 (35.0)	53,798 (36.9)
90–99	5764 (8.8)	5732 (8.5)	13,678 (9.4)
≥100	1297 (2.0)	1618 (2.4)	3351 (2.3)
No. of concomitant medicines^a^ (%)			
0	5171 (7.9)	16,072 (24.0)	20,662 (14.2)
1–3	12,858 (19.7)	10,222 (15.2)	26,686 (18.3)
4–6	14,446 (22.1)	12,572 (18.7)	29,125 (20.0)
7–9	12,375 (19.0)	10,930 (16.3)	26,049 (17.8)
10–12	8788 (13.5)	7675 (11.4)	18,868 (12.9)
≥13	11,657 (17.9)	9594 (14.3)	24,569 (16.8)
Cholesterol measurement^a^ (HDL/LDL) (%)			
≤3.5	39,143 (59.9)	42,892 (64.0)	86,247 (59.1)
3.6–5	20,792 (31.8)	18,630 (27.8)	46,654 (32.0)
>5	5360 (8.2)	5543 (8.3)	13,058 (8.9)
No. of previous fractures^b^ (%)			
0	46,551 (71.3)	NA	112,120 (76.8)
1	9763 (15.0)	NA	18,129 (12.4)
≥2	8981 (13.8)	NA	15,710 (10.8)

OST = patients with incident diagnosis of osteoporosis; IFX = patients with incident fragility fracture; OBP = incident users of oral bisphosphonates; SES = socioeconomic status; MI = myocardial infarction; BMI = body mass index; eGFR = estimated glomerular filtration rate; SBP = cholesterol, systolic blood pressure; DBP = diastolic blood pressure.

^a^ In the year before start.

^b^ Ever.

### Outcomes

The main outcome of the study was 1‐year occurrence of MACE, a composite outcome of the first occurrence of either stroke, MI, or death due to CVD (recorded as the primary cause of death in ONS).

Additionally, secondary analysis of 2‐year occurrence of MACE and sensitivity analysis excluding death (MI/stroke) at 1 and 2 years is reported in the Supplemental Data: MI/stroke, a composite outcome of the first occurrence of either stroke or MI.

### Statistical analysis

Baseline characteristics of all three cohorts were described.

#### Estimation of incidence rates

Incidence rates (IR) and their 95% confidence interval of each outcome at 1 and 2 years after index date were calculated (cases/1000 person‐years) through ERIC Notebook person‐time methodology.^(^ ^42^ ^)^

#### Construction of the prediction models

Performance of QRISK variables to estimate the 1‐year risk of MACE was assessed (QRISK variables are listed in Supplemental Table S1). Finally, all the available candidate risk factors described in the above were combined into a prediction model (henceforth referred to as “ALL”). Lasso regression selected the key risk factors, which were then entered into a final logistic regression equation. Model performance was evaluated for this final equation and model coefficients and intercept terms reported. Missing data were handled using multiple imputation and combined using Rubin's rules as required.^(^ ^43^ ^)^ The data were split 50‐50 into training and validation datasets. All models were developed using training data and tested using the validation data. Supplemental Fig. S1 describes the steps used to build the final model. Further explanations of the prediction model development are described in Supplemental Materials.

The same steps were repeated for 2‐year MACE (secondary analysis), for 1‐ and 2‐year MI/stroke outcomes (sensitivity analysis) and for sex‐based models, included in Supplemental Materials.

#### Model performance

We assessed the models internally using the validation data sets. Discrimination was evaluated by calculating the area under the curve (AUC). The AUC was produced for all 20 validation data sets, then pooled using Rubin's rules. Calibration was assessed by producing calibration plots of observed versus predicted probabilities, in tenths of predicted risk. Calibration plots were also produced for 10‐year age groups and sex.

All statistical analyses took place in R version 3.6.0, including MICE, glmnet, rpart, gbm, caret, flextable, pROC, and officer packages.

### Patient and public involvement

Used data were previously collected and all participant records were linked‐anonymized. Hence, no patients or members of the public were directly implicated in the design or analysis of the reported data.

## Results

**Fig. 1:**
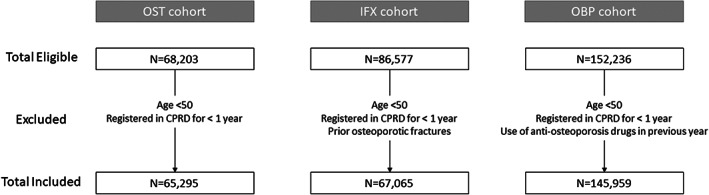
Study flow chart. OST = patients with incident diagnosis of osteoporosis; IFX = patients with incident fragility fracture; OBP = incident users of oral bisphosphonates; CPRD = clinical practice research datalink.

A total of 65,295, 67,065, and 145,959 participants were included in the OST, IFX, and OBP cohorts, respectively (Fig 1). Most of the cohorts were populated by women (OST: 86.80%; IFX: 76.7%; OBP: 79.8%). The IFX cohort had the older population (mean age, years [standard deviation]: 79.52 [11.01]), followed by the OBP (74.35 [10.88]) and OST (73.05 [10.67]). Baseline characteristics of each cohort are shown in Table 1. Baseline characteristics stratified by outcome for the development and validation data sets are provided in Supplemental Table S2*A–C*.

**Fig. 2:**
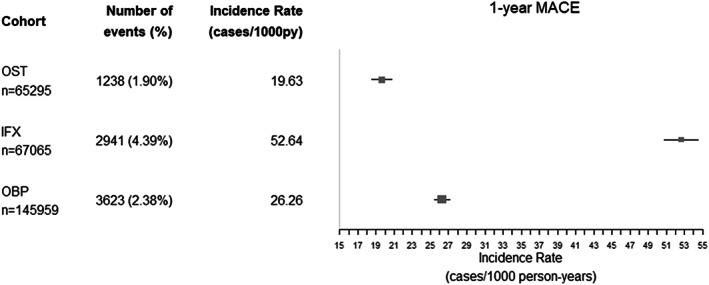
Incidence rates of MACE after 1 year of follow‐up. Incidence rate is reported with 95% confidence intervals. OST = patients with incident diagnosis of osteoporosis; IFX = patients with incident fragility fracture; OBP = incident users of oral bisphosphonates; MACE = composite outcome for the occurrence of either myocardial infarction, stroke, or cardiovascular disease death.

At 1‐year follow‐up, MACE IR (95% confidence interval [CI], in units of cases/1000 person‐years) was 19.6 (18.5–20.7) in OST, 26.3 (25.4–27.1) in OBP, and 52.6 (50.7–54.5) in the IFX cohort (Fig. 2). IFX cohort also had the highest incidences when stratified by age groups (Supplemental Fig. S2). Those who experienced MACE were older in general (higher proportion aged >75 years) with higher comorbidity (Charlson score) and higher prevalence of drug use (eg, antihypertensives and beta‐blockers).

Supplemental Fig. S3 display the IRs of 2‐year MACE, 1‐ and 2‐year stroke/MI, and MACE and stroke/MI outcomes stratified by sex. Overall, despite the considerably lower proportion of males in this study, they suffered higher incidence rates of both outcomes in the 1‐ and 2‐year follow‐up periods.

### Development and performance of the prediction models

Predictive models using risk factors identified from lasso reach AUC values above 70% for the three models, and these equalled or outperformed the models using just QRISK factors (Supplemental Fig. S4). MACE models for OST and OBP populations had higher discrimination values compared with the stroke/MI models (Supplemental Fig. S5), whereas AUC values in IFX model were better for the stroke/MI outcomes. Differences between 1‐ and 2‐year prediction models were minimal with no apparent pattern; however, sex‐based models (Supplemental Fig. S5*B*, *C*) show lower AUC values for men‐only models. It could be related to the considerably lower sample size of those cohorts.

Supplemental Figs. S6 and S7 report 1‐year MACE calibration plots by age and by age and sex, respectively. Supplemental Figs. S8–S10 present the calibration plots of 2‐year MACE and MI/stroke models. Generally, models were well‐calibrated, with an overpredicting risk for the population <60 years old and underpredicting for those aged >80 years, probably caused by the lower proportion of participants belonging to either category.

### Selected risk factors for patients at high risk of fracture

Table 2 displays the risk factors selected from lasso for the overall models of 1‐year MACE along with their odd ratios (OR) and confidence intervals, and Supplemental Table S3 lists its beta coefficients. Sex, age, smoking, and drinking in the prior year, atrial fibrillation diagnosis, use of antihypertensive medication, prior MI or stroke, CVD history, number of concomitant medicines in the prior year, and estimated glomerular filtration rate (eGFR), systolic blood pressure (SBP), and cholesterol measurements in the prior year, appeared in all models. Predictors common in two of the three cohort models were body mass index (BMI), beta‐blocker use, number of general practitioner (GP) visits, number of GP emergency visits, and diastolic blood pressure (DBP) measurements in the year before start.

**Table 2:** Predictors of 1‐Year MACE Overall Models (Risk Factors Selected by Lasso Regression)

Predictor	OST OR (95% CI)	IFX OR (95% CI)	OBP OR (95% CI)
Sex, male (%)	1.61 (1.3, 2)	1.29 (1.12, 1.49)	1.42 (1.25, 1.61)
Age group, years (%)			
50–59	Ref	Ref	Ref
60–69	1.36 (0.79, 2.35)	7.66 (3.05, 19.23)	1.58 (1.14, 2.19)
70–79	3.07 (1.85, 5.11)	12.56 (5.01, 31.5)	2.46 (1.79, 3.36)
80–89	4.9 (2.9, 8.28)	20.39 (7.84, 53)	4.26 (3.09, 5.87)
>89	7.88 (4.44, 13.99)	27.17 (9.92, 74.38)	5.79 (4.06, 8.24)
SES (%)	x	x	
1	x	x	Ref
2	x	x	1.17 (1.02, 1.36)
3	x	x	1.28 (1.11, 1.48)
4	x	x	1.34 (1.15, 1.56)
5	x	x	1.29 (1.08, 1.54)
Smoking^a^		x	
Ex	Ref	Ref	Ref
No	0.93 (0.73, 1.18)	1 (0.85, 1.18)	0.98 (0.79, 1.23)
Yes	1.42 (1.02, 1.98)	1.16 (0.89, 1.5)	1.14 (0.89, 1.47)
Drinking^a^			
Ex	Ref	Ref	Ref
No	1.19 (0.7, 2)	1.14 (0.85, 1.54)	1.03 (0.71, 1.49)
Yes	0.84 (0.51, 1.39)	1.11 (0.79, 1.57)	0.92 (0.65, 1.31)
Diabetes type I^b^	x	x	x
Diabetes type II^b^	x	x	1.11 (0.9, 1.36)
Chronic obstructive pulmonary disease^b^	x	x	1.05 (0.87, 1.27)
Chronic kidney disease^b^	x	x	0.81 (0.61, 1.06)
Rheumatoid arthritis^b^	x	x	0.88 (0.74, 1.04)
Lupus^b^	x	x	x
Systemic heart disease^a^	x	x	0.62 (0.37, 1.03)
Anti‐osteoporosis use^a^	x	x	x
Heparin use^a^	x	x	x
Beta‐blocker use^a^	x	1.11 (0.95, 1.29)	1.16 (1.02, 1.33)
Hypertension^a^	x	x	x
Deep vein thrombosis or pulmonary embolism^a^	x	x	x
Anticoagulant use^a^	x	x	0.93 (0.75, 1.14)
Antidepressants TCA^a^	x	x	x
Antidepressants SSRI^a^	x	x	1.28 (1.09, 1.49)
Hypercholesterolemia^a^	x	x	x
Statin use^a^	x	x	x
Osteoporosis history^b^	x	x	0.74 (0.66, 0.83)
Family history of cardiovascular disease	x	x	0.94 (0.78, 1.14)
Family history of cardiovascular disease before age 60 years	x	x	x
Heart failure^b^	x	x	1.03 (0.84, 1.27)
Migraine^b^	x	x	x
Severe mental illness^b^	x	x	x
Vascular disease^b^	x	x	x
Atrial fibrillation^b^	1.61 (1.27, 2.05)	1.2 (1.01, 1.43)	1.29 (1.08, 1.55)
On antihypertensive drug	1.23 (0.97, 1.55)	1.08 (0.87, 1.33)	1.05 (0.9, 1.22)
Antipsychotic use^a^	X	X	x
Steroid use^a^		X	x
Erectile dysfunction^a^	X	X	x
Charlson score	x		
0	x	x	Ref
1	x	x	1.01 (0.87, 1.17)
2	x	x	0.94 (0.79, 1.13)
≥3	x	x	1.06 (0.85, 1.31)
Cardiovascular disease	x	x	
No	x	x	Ref
Ever >1 year before index date	x	x	1.16 (1, 1.36)
1 year before index	x	x	1.18 (0.84, 1.66)
6 months before index	x	x	1.52 (1.14, 2.03)
1 month before index	x	x	2.18 (1.49, 3.21)
MI or stroke			
No	Ref	Ref	Ref
Ever >1 year before index date	0.99 (0.7, 1.4)	1.23 (0.98, 1.55)	1.34 (1.09, 1.64)
1 year before index	2.03 (1.38, 2.99)	1.56 (1.19, 2.03)	2.52 (2, 3.18)
Established CVD^b^	1.9 (1.46, 2.48)	1.7 (1.42, 2.04)	1.49 (1.25, 1.79)
Any fracture history	x	x	x
No	x	x	x
Ever >1 year before index date	x	x	x
1 year before index	x	x	x
Hip fracture history	x	x	x
No	x	x	x
Ever >1 year before index date	x	x	x
1 year before index	x	x	x
Shoulder fracture history	x	x	x
No	x	x	x
Ever >1 year before index date	x	x	x
1 year before index	x	x	x
Spine fracture history	x	x	x
No	x	x	x
Ever >1 year before index date	x	x	x
1 year before index	x	x	x
Wrist fracture history	x	x	x
No	x	x	x
Ever >1 year before index date	x	x	x
1 year before index	x	x	x
BMI^a^		x	
<18.5	Ref	x	Ref
18.6–24.9	0.72 (0.49, 1.05)	x	0.74 (0.57, 0.96)
25–29.9	0.56 (0.35, 0.9)	x	0.58 (0.41, 0.81)
30–39.9	0.42 (0.2, 0.85)	x	0.55 (0.36, 0.84)
≥40	0.66 (0.22, 1.93)	x	0.39 (0.17, 0.91)
No. of GP visits^a^			
0	Ref	x	Ref
1–5	1.01 (0.61, 1.66)	x	0.9 (0.73, 1.1)
6–10	0.94 (0.56, 1.59)	x	0.78 (0.61, 1.01)
11–15	0.95 (0.55, 1.62)	x	0.78 (0.6, 1.01)
≥16	1.07 (0.63, 1.81)	x	0.86 (0.67, 1.12)
No. of GP emergency visits^a^	x		
0	x	Ref	Ref
1	x	1.25 (1.05, 1.47)	1.15 (0.99, 1.35)
2	x	1.43 (1.15, 1.78)	1.4 (1.13, 1.74)
3–5	x	1.21 (0.97, 1.5)	1.47 (1.2, 1.8)
≥6	x	1.13 (0.82, 1.56)	2.53 (1.96, 3.26)
eGFR^a^			
≤29	Ref	Ref	Ref
30–44	1.11 (0.44, 2.81)	0.87 (0.61, 1.24)	0.91 (0.56, 1.5)
45–59	0.89 (0.37, 2.15)	0.7 (0.38, 1.27)	0.83 (0.49, 1.4)
60–89	0.76 (0.31, 1.86)	0.59 (0.17, 2.01)	0.58 (0.27, 1.27)
≥90	0.79 (0.3, 2.07)	0.52 (0.13, 2.13)	0.5 (0.21, 1.22)
SBP^a^			
<120	Ref	Ref	Ref
120–139	1.19 (0.88, 1.61)	1.04 (0.85, 1.28)	1.09 (0.92, 1.31)
140–159	1.32 (0.98, 1.77)	1.2 (0.95, 1.51)	1.24 (1.02, 1.52)
≥160	1.32 (0.91, 1.89)	1.54 (1.11, 2.14)	1.39 (1.09, 1.77)
DBP^a^	x		
<80	x	Ref	Ref
80–89	x	1.04 (0.89, 1.21)	0.94 (0.82, 1.08)
90–99	x	1.18 (0.91, 1.54)	1.05 (0.84, 1.31)
≥100	x	1.13 (0.71, 1.79)	1.31 (0.89, 1.93)
No. of concomitant medicines^a^			
0	Ref	Ref	Ref
1–3	0.69 (0.42, 1.12)	1.14 (0.9, 1.44)	0.87 (0.7, 1.07)
4–6	0.83 (0.51, 1.36)	1.29 (1.03, 1.63)	0.75 (0.59, 0.96)
7–9	1.01 (0.61, 1.66)	1.12 (0.87, 1.43)	0.88 (0.68, 1.14)
10–12	1.12 (0.67, 1.87)	1.26 (0.96, 1.65)	1 (0.76, 1.31)
≥13	1.11 (0.66, 1.88)	1.25 (0.95, 1.66)	0.98 (0.74, 1.31)
Cholesterol measurement^a^ (HDL/LDL)		x	
≤3.5	Ref	Ref	Ref
3.6–5	1.34 (0.92, 1.95)	1.07 (0.56, 2.04)	1.2 (0.98, 1.46)
>5	1.74 (0.94, 3.2)	1.54 (0.38, 6.31)	1.35 (0.89, 2.03)
No. of previous fractures^b^			x
0	Ref	x	x
1	1.24 (1.01, 1.53)	x	x
≥2	0.99 (0.79, 1.25)	x	x

OST = patients with incident diagnosis of osteoporosis; IFX = patients with incident fragility fracture; OBP = incident users of oral bisphosphonates; OR = odds ratio; CI = confidence intervals; MACE = composite outcome for the occurrence of either myocardial infarction, stroke, or cardiovascular disease death; SES = socioeconomic status; MI = myocardial infarction; BMI = body mass index; eGFR = estimated glomerular filtration rate; SBP = cholesterol, systolic blood pressure; DBP = diastolic blood pressure.

^a^ In the year before start.

^b^ Ever.

Supplemental Table S4*A, B* summarizes risk factors selected from lasso and ORs for 2‐year MACE and for MI/stroke models, respectively, and Supplemental Table S5*A, B* lists its beta coefficients. Supplemental Table S6*A–C* reports all sex‐based models and Supplemental Table S7*A–C* lists its beta coefficients.

Detailed explanation and an example of how to obtain an estimate for an individual is reported in Supplemental Materials.

## Discussion

In this study, we evaluated the incidence of major adverse cardiovascular events though a composite outcome, MACE,^(^ ^44^ ^)^ and assessed risk factors of CVD to predict this outcome at 1 year in three different cohorts. The IFX cohort can be used for a secondary fracture prevention program, the OBP cohort has the potential to be used in primary prevention because it approximates patients newly diagnosed and treated for osteoporosis, and the OST cohort can be used as a general screening in primary care.

We observed that incidence of MACE was slightly higher at 1 year than at 2 years, especially for IFX cohort. When stratifying by sex, men had higher incidence rates than women, which agrees with results published by the British Health Foundation, where male incidences at UK in 2017 were higher than female.^(^ ^45^ ^)^ Prior studies using CPRD show that the general population aged 70+ years had an IR of MACE of 15.1 (per 1000 person‐years),^(^ ^32^ ^)^ whereas our study populations including younger individuals (ie, age 50+ patients) have higher IR. Specifically the IFX cohort had the highest incidence for MACE (51.1/1000 person‐years), which could be explained by this cohort having an older age (71% were older than 75 years) and the largest proportion of men (23.3%) among the three cohorts, followed by OBP (26.3–20.2%) and OST (19.6–13.2%) cohorts. The observation of higher incidences in IFX cohort was consistent when IR of each study cohort was stratified by age groups.

Fitting the list of risk factors from QRISK into a prediction model for 1‐year MACE events, we obtained AUC values of 0.73, 0.67, and 0.71 in OST, IFX, and OBP cohorts, respectively. However, starting from the list of “ALL” risk factors for CVD available in CPRD and selecting the most important through lasso regression, we obtained model equations that exceed QRISK (AUC in selected risk factors from ALL set: 0.75 in OST, 0.70 in IFX, and 0.75 in OBP). This list included generic features as well as those specific to the study population, and all of them can be found readily in primary care data. Among them, age had the largest statistically significant effect size.

Comparing our models to existing cardiovascular prediction tools, we found that the performances of Framingham and QRISK studies were higher in the general population: AUC >0.76 and >0.86, respectively.^(^ ^5^, ^11^ ^)^ Framingham equations have been validated and recalibrated multiple times using different populations,^(^ ^46^ ^)^ whereas QRISK has a higher accuracy for UK population than the Framingham tool.^(^ ^47^ ^)^ However, neither tool was developed for the osteoporotic/fracture risk population, and neither includes specific risk factors (eg prior fractures and alcohol consumption) for these particular patients, in whom short‐term cardiovascular risk might be over‐ or underestimated. In fact, Framingham only permits risk calculation over long periods, and there are no studies extrapolating to shorter risk intervals.

The need of specific CVD tools for populations at higher fracture risk and at short‐term can be rationalised by the lower performance of the models using the QRISK list (ie, using the predictors selected for general population). This may be particularly interesting to note in our sensitivity analysis, which uses an outcome closer to the QRISK tool: AUC values of MI/Stroke models decrease to a range of 0.62 to 0.70 when applying the QRISK factors to osteoporotic/fracture risk population.

The proposed predictive models have good predictive power and internal validity (discrimination and calibration) in OBP and OST cohorts for 1‐year MACE events (the obtained equations are included in this article), and the IFX models reach the 70% AUC threshold, considered as the minimum acceptable discrimination.^(^ ^32^ ^)^

Secondary and sensitivity analysis show no differences using 2‐year models and better performance of MACE than MI/Stroke models.

The proposed study is observational in nature and hence cannot address causality but rather describe associations. There is no guarantee that all possible risk factors are included, but for all those factors that are, multivariable regression ensures that they are adjusted for (and hence reducing the risk of confounding). The three presented cohorts are not mutually exclusive but encompass the diversity of the population at high risk of fracture, and the different criteria used to evaluate them. Another limitation is the lack of external validity, which can be assessed in future studies to ensure the validity of the models across different populations. The enhanced performance observed in female population was expected due to the higher representation of females in our cohorts. The main strengths of this study are the large sample size and the wide selection of routinely collected potential risk factors included.^(^ ^48^ ^)^

To summarize, incidence rate of MACE events in the studied populations ranged from 19.6 to 52.6, with IFX as the cohort with the higher risk. Efforts in predicting the study events outline the differences between general and the osteoporotic/fracture risk population. The resulting algorithms include risk factors specific to the study population as well as more generic features that can be found easily in primary care data. Further work will focus on validating these models in external cohorts.

## Disclosures

All authors have completed the ICMJE disclosure form at http://www.icmje.org/disclosure-of-interest/ and declare the following interests: MP‐M, LE‐H, AD, CC, and SK have no conflicts to declare. DP‐A reports an institutional grant from NIHR, grants from Chesi‐Taylor and Novartis, grants and other support from Amgen and UCB Biopharma, and other support from Astellas, AstraZeneca, Johnson and Johnson, Janssen—on behalf of IMI‐funded EHDEN and EMIF consortiums—and Synapse Management Partners. MA, CL, and ET are current employees of UCB Biopharma and hold stock shares of the company.

## Author Contributions

**Marta Pineda‐Moncusí:** Writing – original draft; writing – review and editing. **Leena El‐Hussein:** Formal analysis; writing – original draft; writing – review and editing. **Antonella Delmestri:** Data curation; writing – review and editing. **Cyrus Cooper:** Writing – original draft; writing – review and editing. **Alireza Moayyeri:** Conceptualization; writing – original draft; writing – review and editing. **Cesar Libanati:** Conceptualization; writing – original draft; writing – review and editing. **Emese Toth:** Conceptualization; writing – review and editing. **Sara Khalid:** Conceptualization; formal analysis; writing – original draft; writing – review and editing. **Daniel Prieto‐Alhambra:** Conceptualization; writing – review and editing.

### Peer Review

The peer review history for this article is available at https://publons.com/publon/10.1002/jbmr.4648.

## Supporting information


Appendix S1.

**Supplemental Fig. S1.** Steps in the development and validation of the prediction model.
**Supplemental Fig. S2.** Incidence rates by age groups. OST = patients with incident diagnosis of osteoporosis; IFX = patients with incident fragility fracture; OBP = incident users of oral bisphosphonates; MACE = composite outcome for the occurrence of either myocardial infarction, stroke, or cardiovascular disease death; MI = myocardial infarction.
**Supplemental Fig. S3.** Incidence rates of 2‐year MACE, 1‐ and 2‐year stroke/MI; and MACE and stroke/MI by sex. OST = patients with incident diagnosis of osteoporosis; IFX = patients with incident fragility fracture; OBP = incident users of oral bisphosphonates; MACE = composite outcome for the occurrence of either myocardial infarction, stroke, or cardiovascular disease death; MI = myocardial infarction.
**Supplemental Fig. S4.** Area under ROC curve for internal validation of 1‐ and 2‐year MACE outcome using risk factors from QRISK and LASSO models. OST = patients with incident diagnosis of osteoporosis; IFX, patients with incident fragility fracture; OBP, incident users of oral bisphosphonates; AUC, area under the curve; MACE, composite outcome for the occurrence of either myocardial infarction, stroke or cardiovascular disease death; MI, myocardial infarction.
**Supplemental Fig. S5.** Area under ROC curve for internal validation of Stroke/MI outcome, and MACE and Stroke/MI sex‐based models. OST = patients with incident diagnosis of osteoporosis; IFX = patients with incident fragility fracture; OBP = incident users of oral bisphosphonates; MACE = composite outcome for the occurrence of either myocardial infarction, stroke or cardiovascular disease death; MI = myocardial infarction.
**Supplemental Fig. S6.** Calibration curves for internal validation of 1‐year MACE prediction by age deciles. Models using risk factors selected by lasso regression. From left to right: OST, IFX, and OBP cohorts. OST = patients with incident diagnosis of osteoporosis; IFX = patients with incident fragility fracture; OBP = incident users of oral bisphosphonates; MACE = composite outcome for the occurrence of either myocardial infarction, stroke, or cardiovascular disease death.
**Supplemental Fig. S7.** Calibration curves of 1‐year MACE prediction stratified by age and sex. Models using risk factors selected by lasso regression. From left to right: OST, IFX, and OBP cohorts. OST = patients with incident diagnosis of osteoporosis; IFX = patients with incident fragility fracture; OBP = incident users of oral bisphosphonates; MACE = composite outcome for the occurrence of either myocardial infarction, stroke, or cardiovascular disease death.
**Supplemental Fig. S8.** Calibration curves for internal validation of 2‐year MACE prediction in cohorts OST, IFX, and OBP from left to right.
**Supplemental Fig. S9.** Calibration curves for internal validation of MI/stroke prediction by age deciles.
**Supplemental Fig. S10.** Calibration curves of MI/stroke prediction stratified by age and sex.

**Supplemental Table S1.** List of Available Variables Included in QRISK Tool
**Supplemental Table S2a.** Data Set Split Into Train and Test Sets, Stratified by Outcome (OST Cohort)
**Supplemental Table S2b.** Data Set Split Into Train and Test Sets, Stratified by Outcome (IFX Cohort)
**Supplemental Table S2c.** Data Set Split Into Train and Test Sets, Stratified by Outcome (OBP Cohort)
**Supplemental Table S3.** Model Equations of 1‐Year MACE (Risk Factors Selected by Lasso Regression)
**Supplemental Table S4a.** Predictors of 2‐Year MACE Models (Risk Factors Selected by Lasso Regression)
**Supplemental Table S4b.** Predictors of 1‐ and 2‐Year MI/Stroke Models (Risk Factors Selected by Lasso Regression)
**Supplemental Table S5a.** Model Equations for 2‐Year MACE Models (Risk Factors Selected by Lasso Regression)
**Supplemental Table S5b.** Model Equations for 1‐ and 2‐Year MI/Stroke Models (Risk Factors Selected by Lasso Regression)
**Supplemental Table S6a.** Risk Factors Selected by Lasso for 1‐ and 2‐Year Models in Sex‐Based Models (OST Cohort)
**Supplemental Table S6b.** Risk Factors Selected by Lasso for 1‐ and 2‐Year Models in Sex‐Based Models (IFX Cohort)
**Supplemental Table S6c.** Risk Risk Factors Selected by Lasso for 1‐ and 2‐Year Models in Sex‐Based Models (OBP Cohort)
**Supplemental Table S7a.** Model Equations From Lasso Selection for Women‐ and Men‐Based Models (OST cohort)
**Supplemental Table S7b.** Model Equations From Lasso Selection for Women‐ and Men‐Based Models (IFX Cohort)
**Supplemental Table S7c.** Model Equations From Lasso Selection for Women‐ and Men‐Based Models (OBP Cohort)

## Data Availability

Data that supports the findings of this study was provided by UK CPRD database. Availability of data is subject to protocol approval by CPRD's Research Data Governance Process.
