# Supplementary material for: Estimating the Incidence and Key Risk Factors of Cardiovascular Disease in Patients at High Risk of Imminent Fracture Using Routinely Collected Real‐World Data From the UK
Source: J Bone Miner Res. 2022 Sep 8;37(10):1986–96. doi: 10.1002/jbmr.4648 (PMC9826104; doi:10.1002/jbmr.4648)
Supplement: Supplementary file 1 — Appendix S1. [file JBMR-37-1986-s003.docx]

## Details on the analytical steps

Multiple imputation with chain equations was applied to handle missing values for smoking, drinking, estimated Glomerular Filtration Rate (eGFR), body mass index (BMI), cholesterol, systolic blood pressure (SBP) and diastolic blood pressure (DBP) resulting in 20 imputed datasets. Each imputed dataset was then randomly split 50/50 into a development and a validation set. For managing the large amount of risk factors from the set of ALL variables, a lasso regression model was fitted to one imputed development set. The model selected those risk factors as features that resulted in the best area under the curve (AUC) using cross-validation within the development set. The final model was then estimated by applying logistic regression to all 20 imputed development sets using the lasso-selected features and adjusting the estimates and standard errors for the variability between the imputed datasets with Rubin’s rules

## How to use the prediction models

To obtain the estimate risk of an event for an individual patient, the intercept term of the model will be sum to the value of the predictor variables multiplied by their respective coefficients (β):

“**Y ≈ β0 + β1X1 +… + β*n*X*n* + ε”**

*Where* ***Y*** *is the individual risk,* ***β0*** *is the intercept,* ***β1X1 +… + βnXn*** *are the coefficients (β) of the predictive variables (X), and* ***ε*** *the error that cannot be captured by the model.*

To transform the estimate into a probability, will need to use the formula of the logistic transformation:

**1/(1+exp(-(*estimate)*)**

Example of how to use the proposed models:

We’ll going to predict the risk of 1-year MACE for a 72-year-old men who was newly diagnosed with osteoporosis. This patient is a current smoker, do not drink, has a prior CVD history and are using anti-hypertensive drugs. He has a BMI of 27.3, an eGFR value of 65, a SBP value of 141, and had 10 GP visits the prior year. He is using 5 concomitant medicines and had 1 prior fracture.

In this case, we are going to use 1-year MACE prediction for OST population, and the first step is to obtain the values of the coefficients for this patient.

Following table displays the coefficients from 1-year MACE model in OST population, and the specific patient coefficients:

|  | **OST** | **Patient characteristics** | **Patient coefficients** |
| --- | --- | --- | --- |
| **Predictor** | ***Beta coefficients*** | ***(NO = 0) (Yes = 1)*** | ***β0 and βn*Xn*** |
| **Intercept (β0)** | -5.214 |  | -5.214 |
| **Sex = Male (%)** | 0.479 | 1 | 0.479 |
| **Smoking**** |  |  |  |
| **Ex** | ref | 0 | 0.000 |
| **No** | -0.070 | 0 | 0.000 |
| **Yes** | 0.353 | 1 | 0.353 |
| **Drinking**** |  |  |  |
| **Ex** | ref | 0 | 0.000 |
| **No** | 0.171 | 1 | 0.171 |
| **Yes** | -0.170 | 0 | 0.000 |
| **Atrial fibrillation*** | 0.478 | 0 | 0.000 |
| **On anti-hypertensive drug** | 0.203 | 1 | 0.203 |
| **Age Group (%)** |  |  |  |
| **50-59** | ref | 0 | 0.000 |
| **60-69** | 0.311 | 0 | 0.000 |
| **70-79** | 1.122 | 1 | 1.122 |
| **80-89** | 1.589 | 0 | 0.000 |
| **>89** | 2.065 | 0 | 0.000 |
| **MI or Stroke** |  |  |  |
| **No** | ref | 0 | 0.000 |
| **Ever** | -0.009 | 0 | 0.000 |
| **1 year before index** | 0.709 | 0 | 0.000 |
| **Established CVD *** | 0.643 | 1 | 0.643 |
| **BMI**** |  |  |  |
| **<18.5** | ref | 0 | 0.000 |
| **18.6 - 24.9** | -0.334 | 0 | 0.000 |
| **25 - 29.9** | -0.581 | 1 | -0.581 |
| **30 - 39.9** | -0.879 | 0 | 0.000 |
| **>=40** | -0.421 | 0 | 0.000 |
| **No. of GP visits**** |  |  |  |
| **0** | ref | 0 | 0.000 |
| **1-5** | 0.009 | 0 | 0.000 |
| **6-10** | -0.063 | 1 | -0.063 |
| **11-15** | -0.056 | 0 | 0.000 |
| **>=16** | 0.064 | 0 | 0.000 |
| **eGFR**** |  |  |  |
| **<=29** | ref | 0 | 0.000 |
| **30 – 44** | 0.101 | 0 | 0.000 |
| **45 – 59** | -0.112 | 0 | 0.000 |
| **60 – 89** | -0.280 | 1 | -0.280 |
| **>=90** | -0.236 | 0 | 0.000 |
| **SBP**** |  |  |  |
| **<120** | ref | 0 | 0.000 |
| **120 - 139** | 0.175 | 0 | 0.000 |
| **140 - 159** | 0.278 | 1 | 0.278 |
| **>=160** | 0.274 | 0 | 0.000 |
| **No. of concomitant medicines**** |  |  |  |
| **0** | ref | 0 | 0.000 |
| **1 – 3** | -0.377 | 0 | 0.000 |
| **4 – 6** | -0.183 | 1 | -0.183 |
| **7 – 9** | 0.008 | 0 | 0.000 |
| **10 – 12** | 0.111 | 0 | 0.000 |
| **>=13** | 0.105 | 0 | 0.000 |
| **No. of previous fractures*** |  |  |  |
| **0** | ref | 0 | 0.000 |
| **1** | 0.217 | 1 | 0.217 |
| **>=2** | -0.008 | 0 | 0.000 |
| **Abbreviations**: OST, patients with incident diagnosis of osteoporosis; OR, odds ratio; CI, confidence intervals; MACE, composite outcome for the occurrence of either myocardial infarction, stroke or cardiovascular disease death; * ever; ** in the year prior to start; MI, myocardial infarction; BMI, body mass index; eGFR, estimated Glomerular Filtration Rate; SBP, cholesterol, systolic blood pressure; DBP, diastolic blood pressure. | | | |

Once knowing the specific patient coefficients, we are going to sum them (i.e., all values from "Patient coefficients *β0 and βn*Xn"* column) in order to obtain the patient’s estimate.
The result is -2.855.

Then, the estimate is going to be transformed into a probability:

**1/(1+exp(-(*estimate)*) =** 1/(1+exp(-(-2.855))) = 0.054423

Thus, the proposed patient has a 5.4% risk of having a MACE event in the following year after his risk assessment.
