# Supplementary material for: Estimating the Incidence and Key Risk Factors of Cardiovascular Disease in Patients at High Risk of Imminent Fracture Using Routinely Collected Real‐World Data From the UK
Source: J Bone Miner Res. 2022 Sep 8;37(10):1986–96. doi: 10.1002/jbmr.4648 (PMC9826104; doi:10.1002/jbmr.4648)

# Supplemental FIGURES

# Figure S1. Steps in the development and validation of the prediction model.


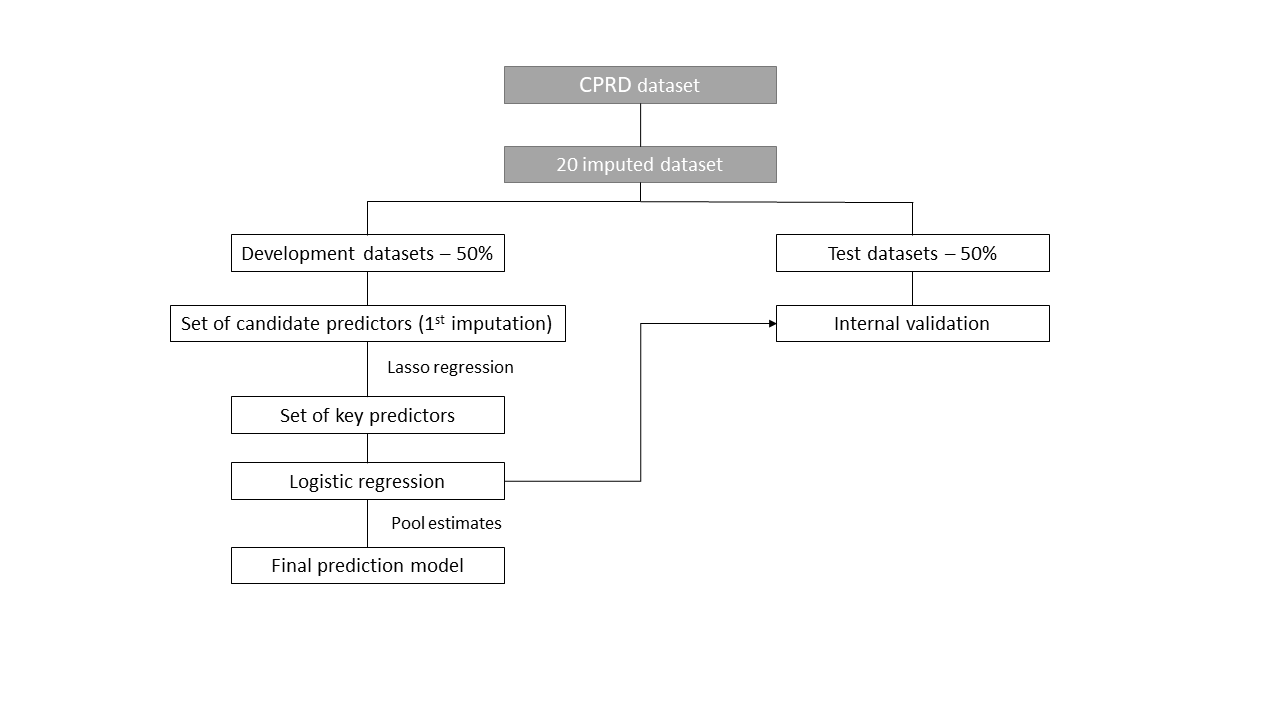


# Figure S2. Incidence rates by age groups. Abbreviations: OST, patients with incident diagnosis of osteoporosis; IFX, patients with incident fragility fracture; OBP, incident users of oral bisphosphonates; MACE, composite outcome for the occurrence of either myocardial infarction, stroke or cardiovascular disease death; MI, myocardial infarction.

## After one year of follow up (MACE)


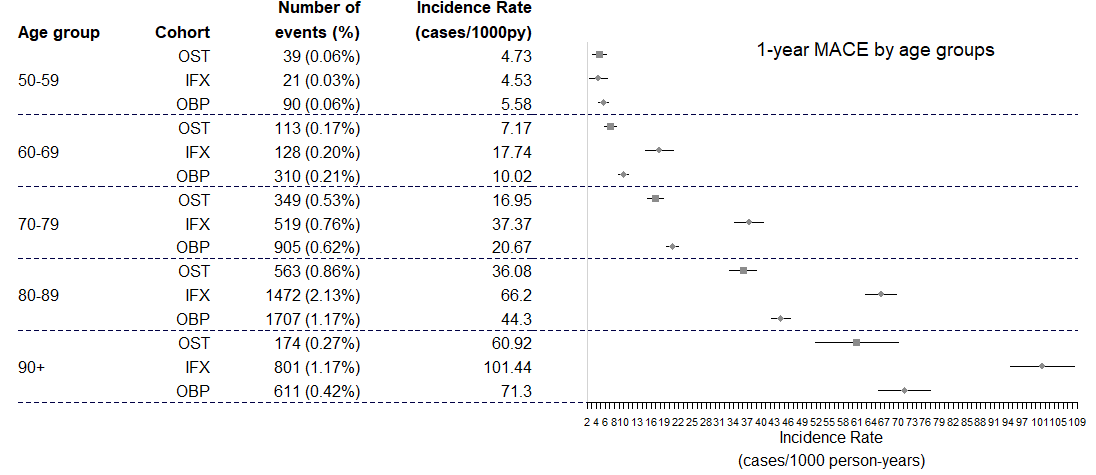


## After two years of follow up (MACE)


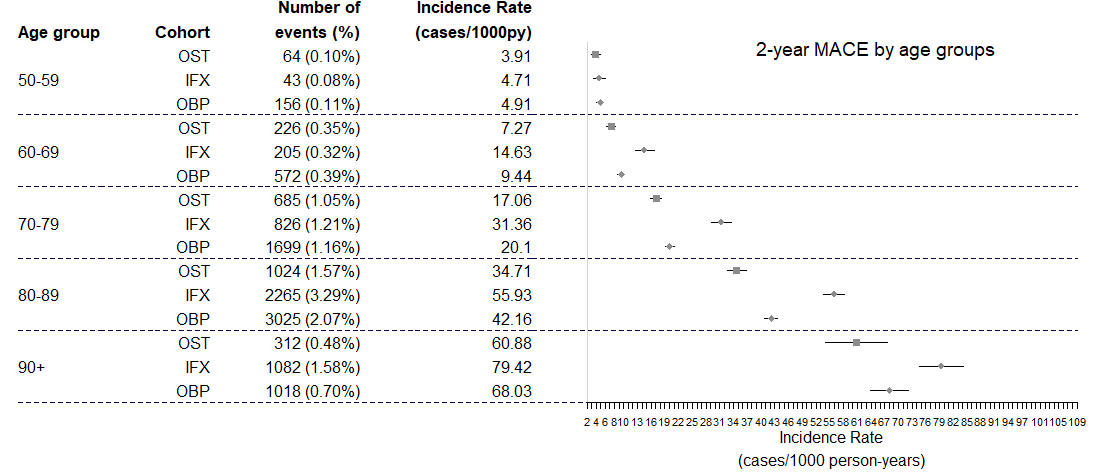


## After one year of follow up (Stroke/MI)


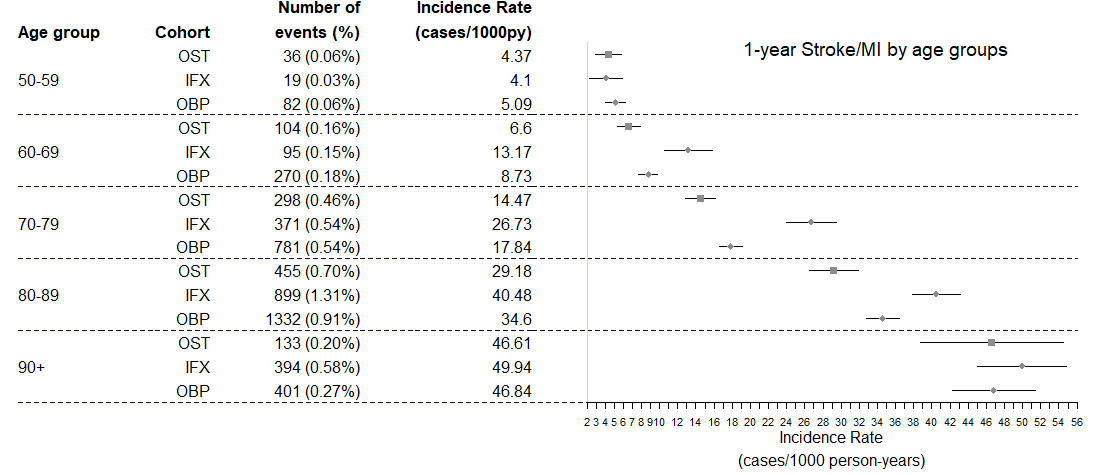


## After two years of follow up (Stroke/MI)


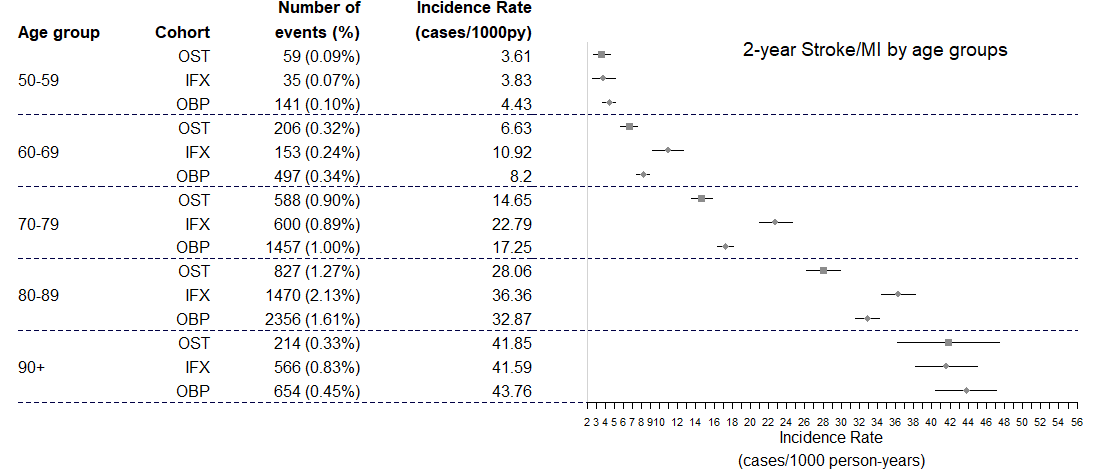


# Figure S3. Incidence rates of two-year MACE, one- and two-year stroke/MI; and MACE and stroke/MI by gender. Abbreviations: OST, patients with incident diagnosis of osteoporosis; IFX, patients with incident fragility fracture; OBP, incident users of oral bisphosphonates; MACE, composite outcome for the occurrence of either myocardial infarction, stroke or cardiovascular disease death; MI, myocardial infarction.

## After two years of follow up (MACE)


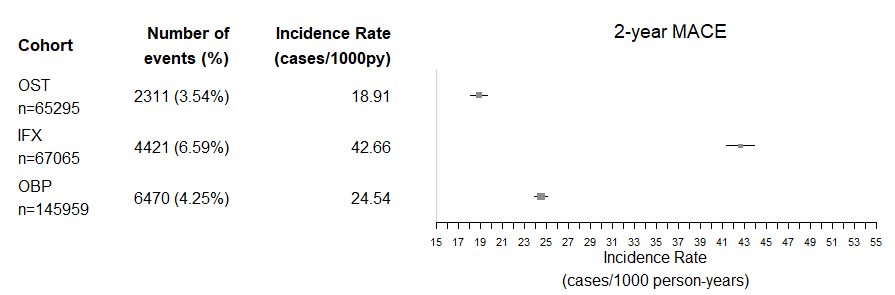


## After one and two years of follow up (Stroke/MI)


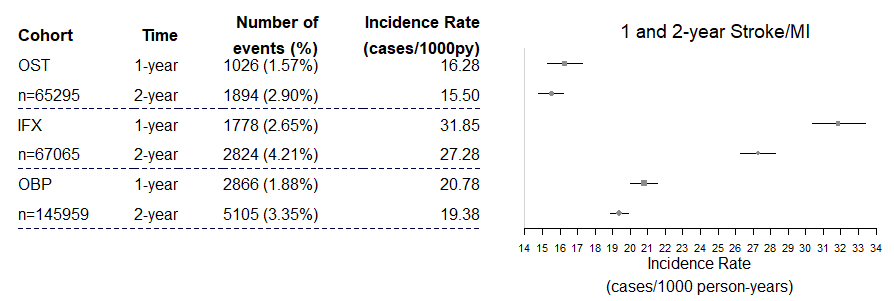


## After one and two years of follow up (Females)


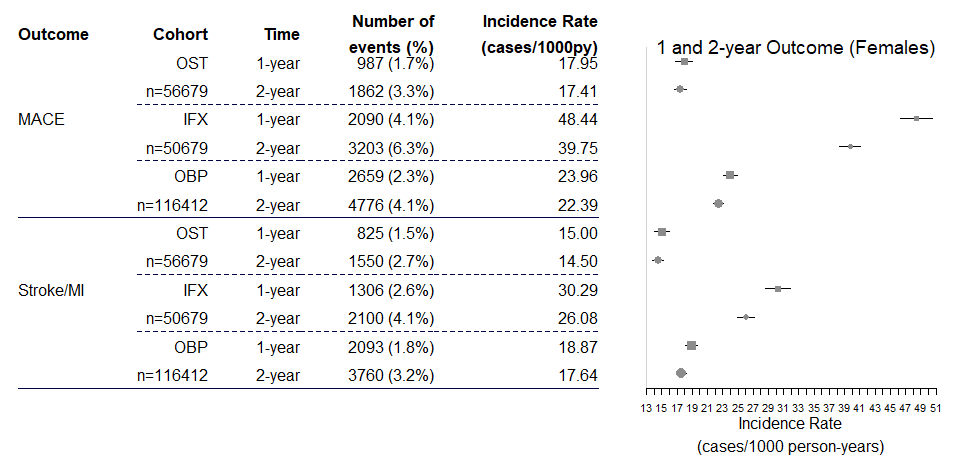


## After one and two years of follow up (Males)


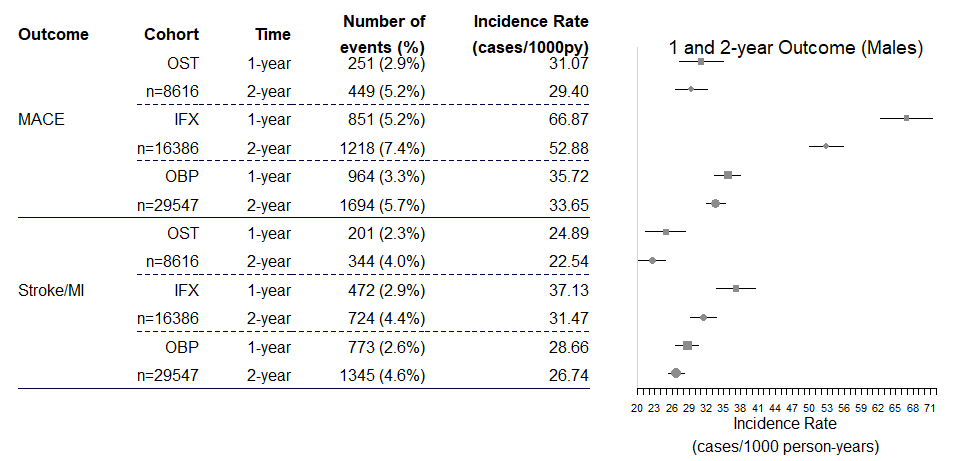


# Figure S4. Area under ROC curve for internal validation of one- and two-years MACE outcome using risk factors from QRISK and LASSO models. Abbreviations: OST, patients with incident diagnosis of osteoporosis; IFX, patients with incident fragility fracture; OBP, incident users of oral bisphosphonates; AUC, area under the curve; MACE, composite outcome for the occurrence of either myocardial infarction, stroke or cardiovascular disease death; MI, myocardial infarction.


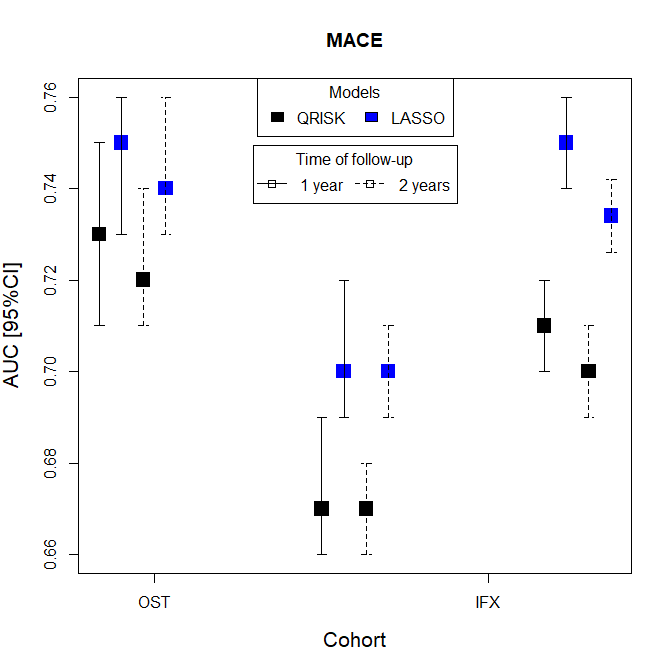


# Figure S5. Area under ROC curve for internal validation of Stroke/MI outcome, and MACE and Stroke/MI gender-based models. Abbreviations: OST, patients with incident diagnosis of osteoporosis; IFX, patients with incident fragility fracture; OBP, incident users of oral bisphosphonates; MACE, composite outcome for the occurrence of either myocardial infarction, stroke or cardiovascular disease death; MI, myocardial infarction.

## One- and two-year Stroke/MI


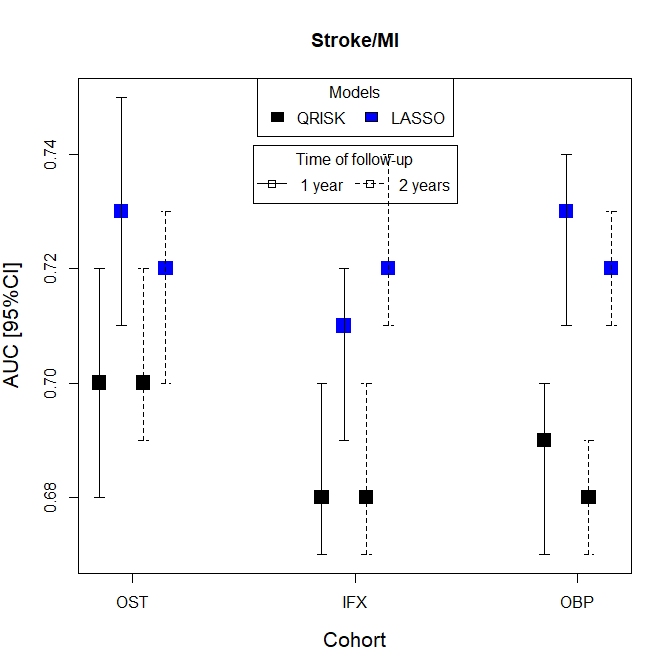


## One- and two-year MACE (gender-based models)


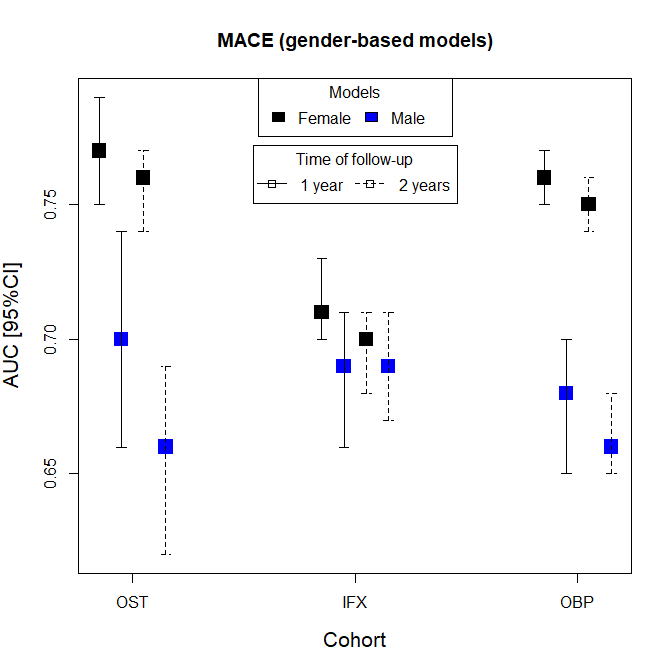


## One- and two-year Stroke/MI (gender-based models)


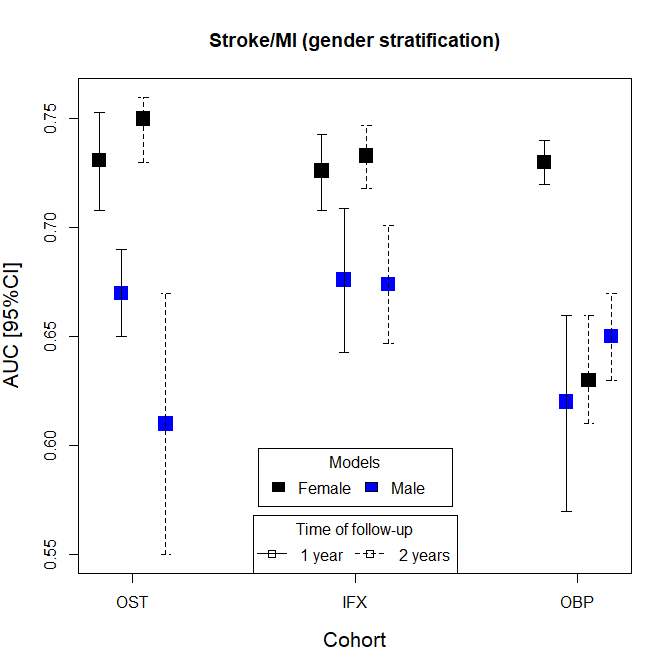


# Figure S6. Calibration curves for internal validation of one-year MACE prediction by age deciles. Models using risk factors selected by lasso regression. From left to right: OST, IFX and OBP cohorts. Abbreviations: OST, patients with incident diagnosis of osteoporosis; IFX, patients with incident fragility fracture; OBP, incident users of oral bisphosphonates; MACE, composite outcome for the occurrence of either myocardial infarction, stroke or cardiovascular disease death.


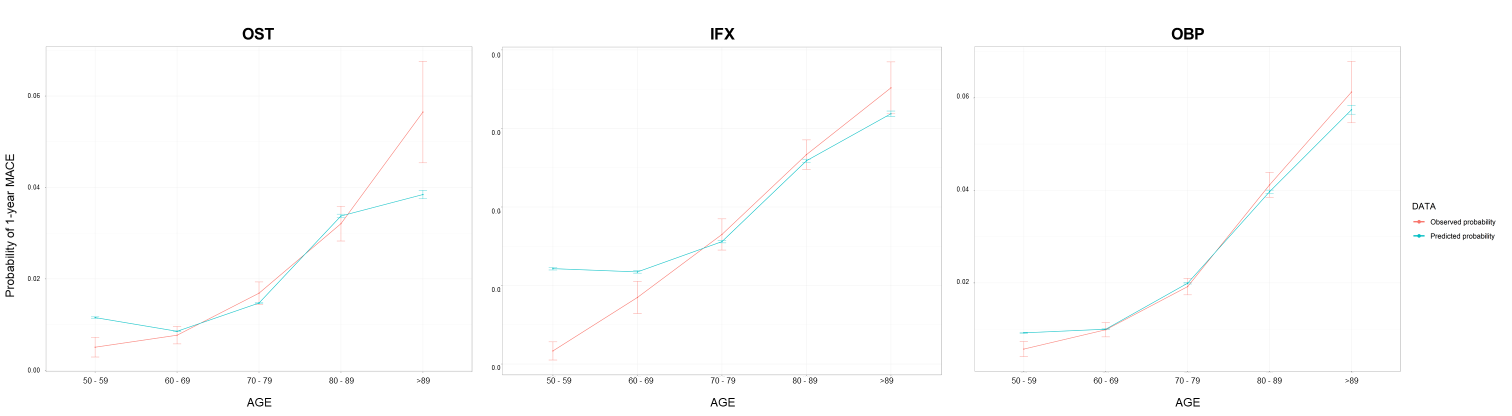


# Figure S7. Calibration curves of one-year MACE prediction stratified by age and gender. Models using risk factors selected by lasso regression. From left to right: OST, IFX and OBP cohorts. Abbreviations: OST, patients with incident diagnosis of osteoporosis; IFX, patients with incident fragility fracture; OBP, incident users of oral bisphosphonates; MACE, composite outcome for the occurrence of either myocardial infarction, stroke or cardiovascular disease death.


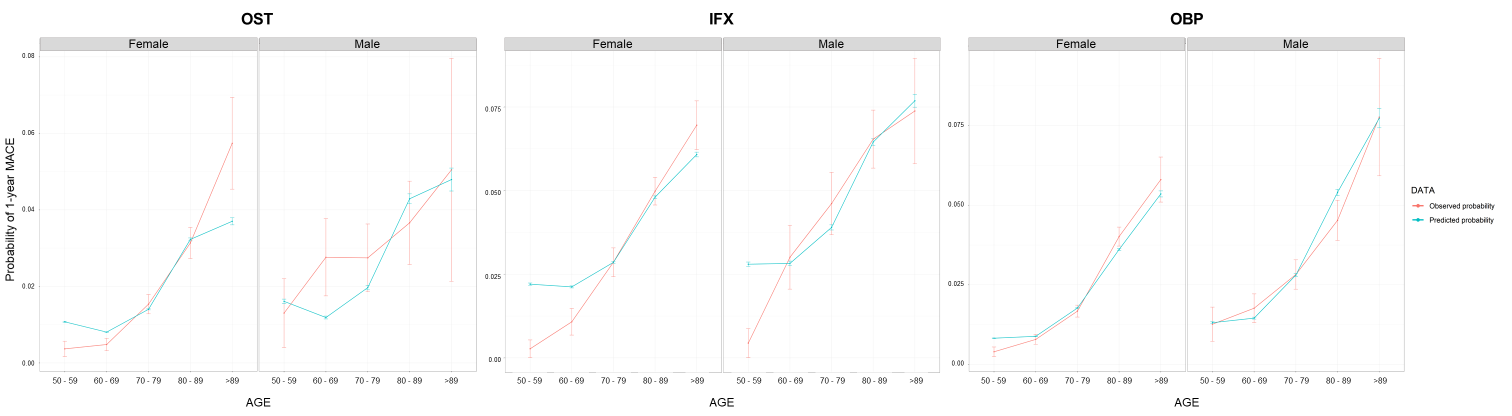


# Figure S8. Calibration curves for internal validation of two-year MACE prediction in cohorts OST, IFX and OBP from left to right

## Two-year MACE1 prediction by age deciles


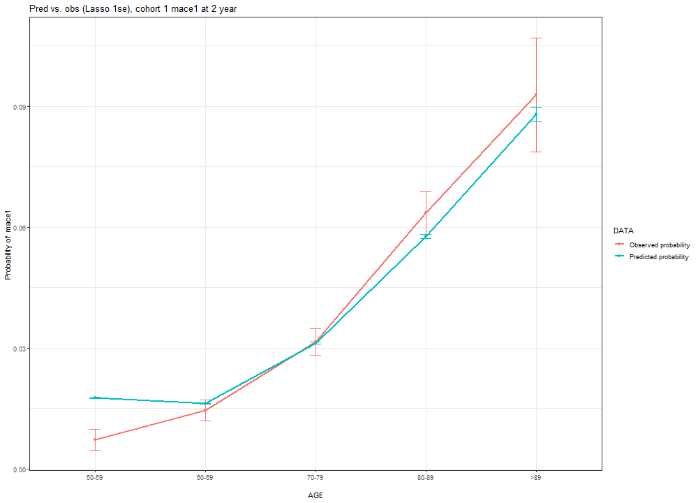

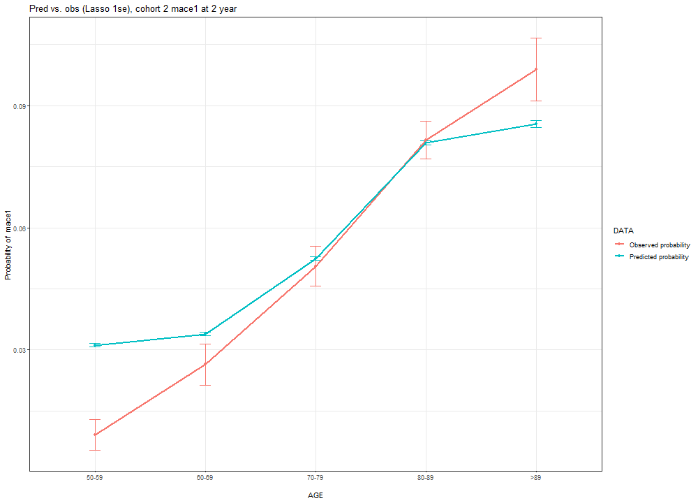

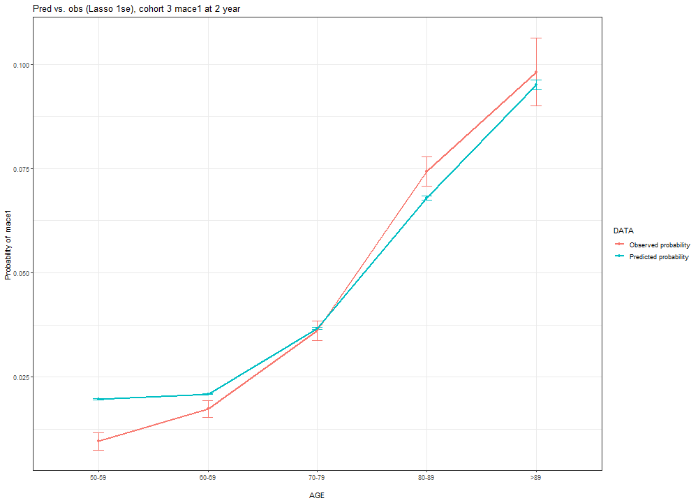


## Two-year MACE1 prediction by age and gender


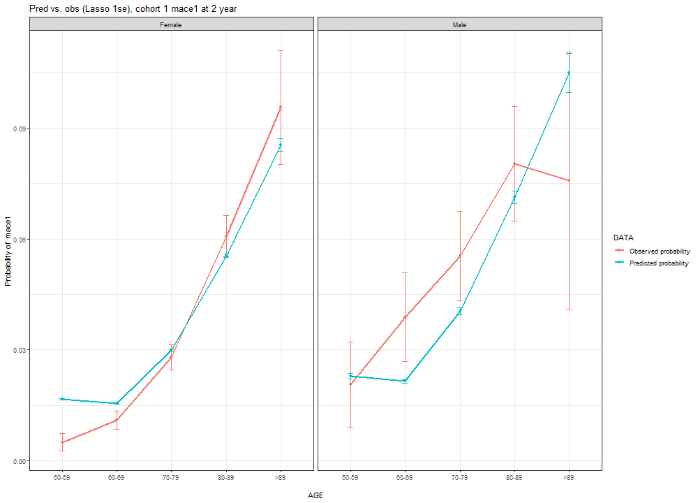

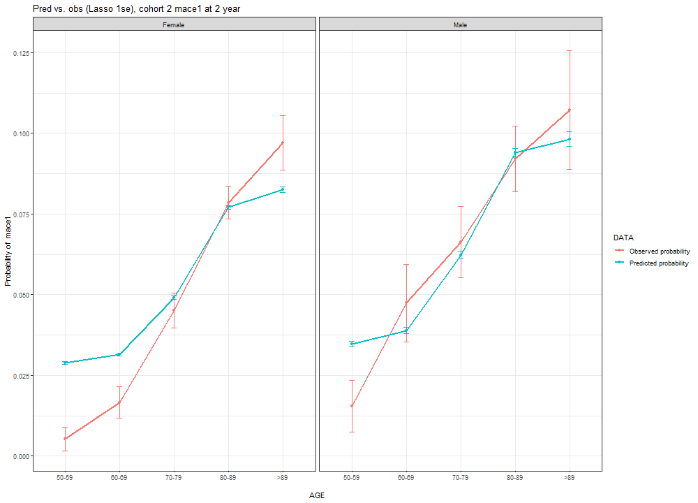

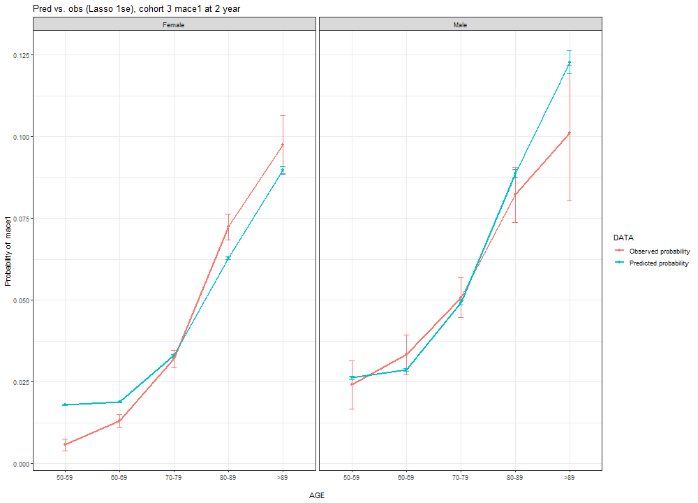


# Figure S9. Calibration curves for internal validation of MI/stroke prediction by age deciles

## One-year MI/stroke prediction cohorts OST, IFX and OBP from left to right


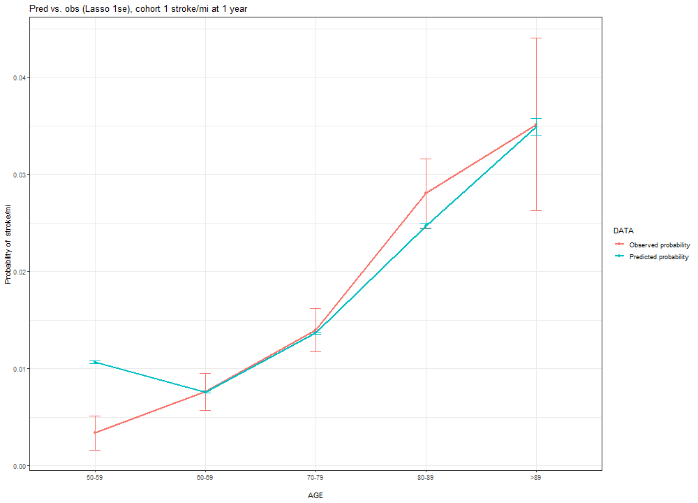

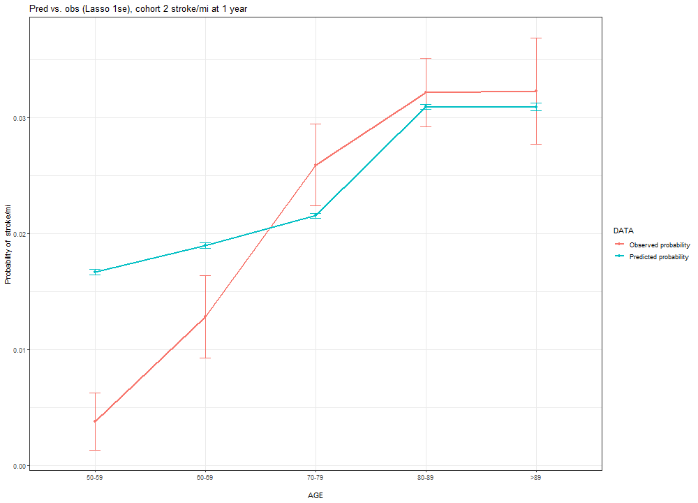

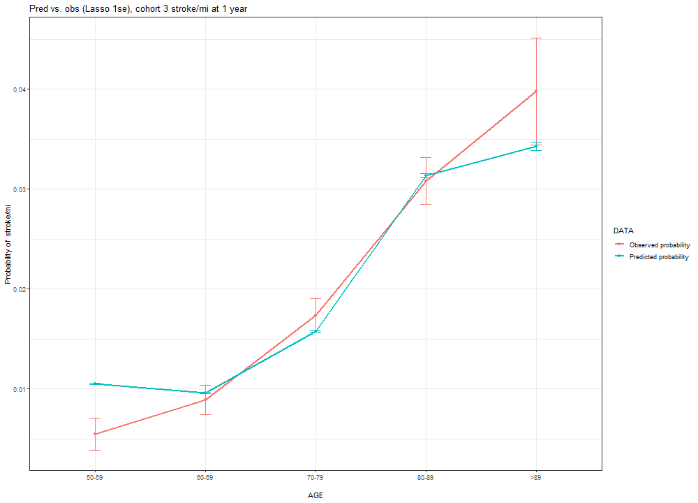


## Two-year MI/stroke prediction in cohorts OST, IFX and OBP from left to right


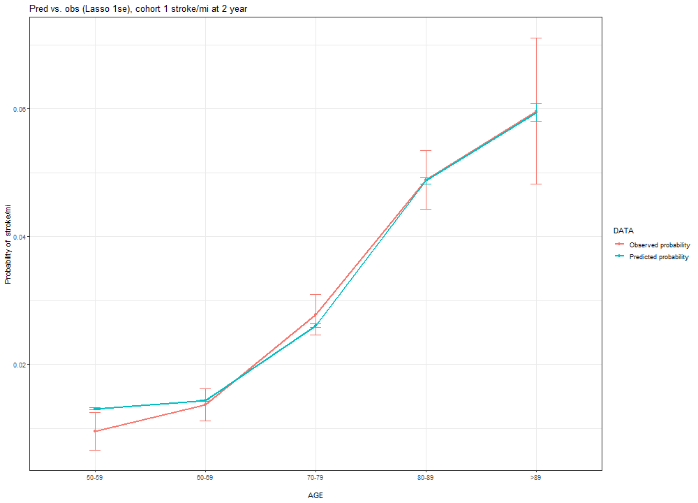

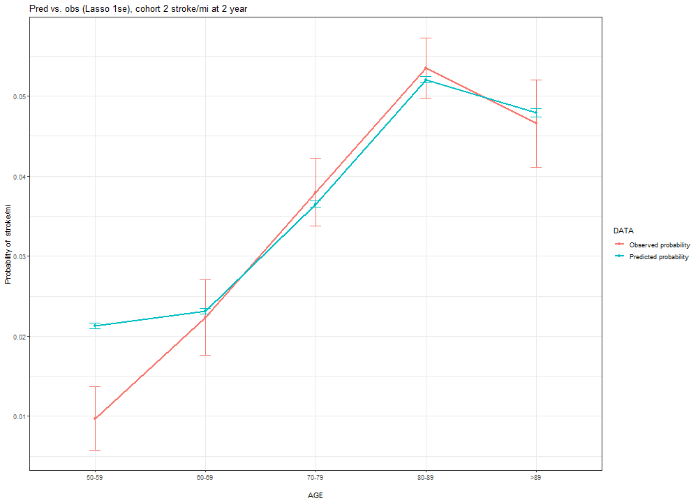

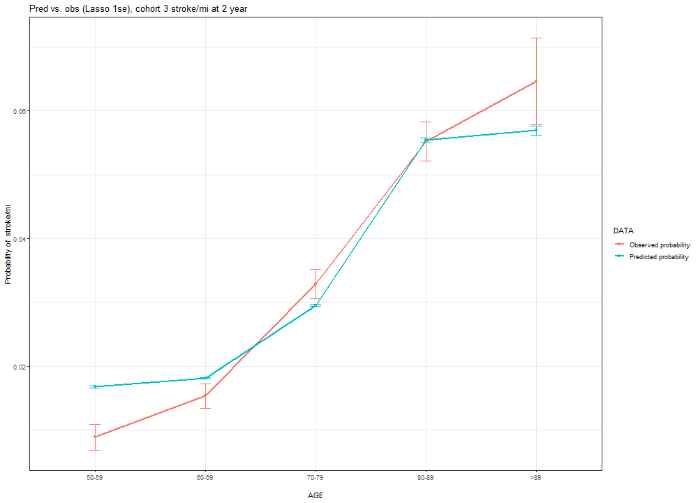


# Figure S10. Calibration curves of MI/stroke prediction stratified by age and gender

## One-year MI/stroke prediction in cohorts OST, IFX and OBP from left to right


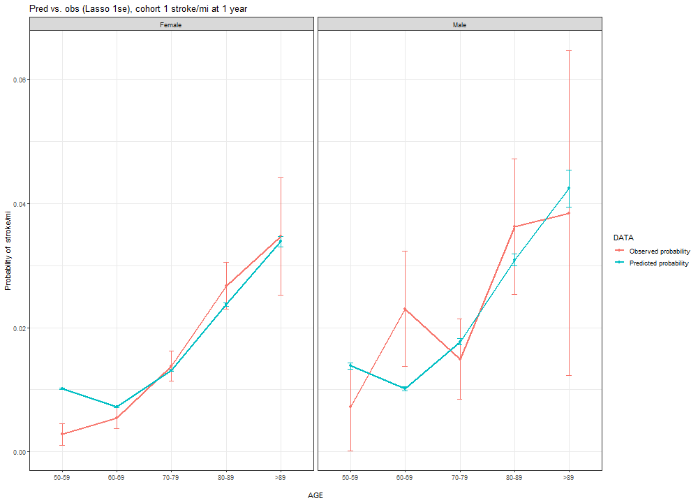

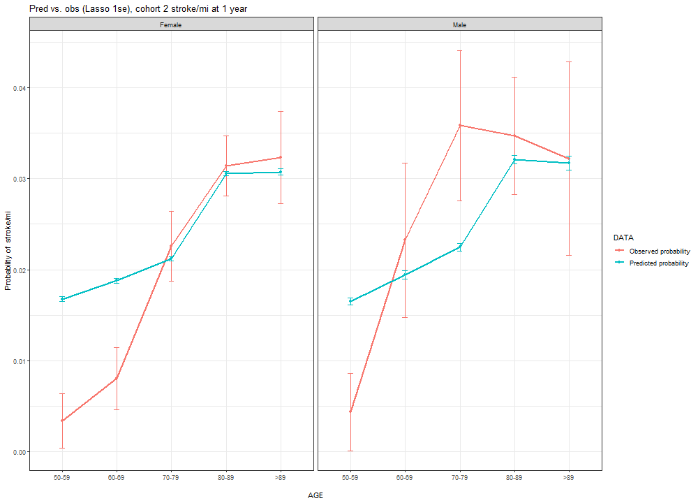

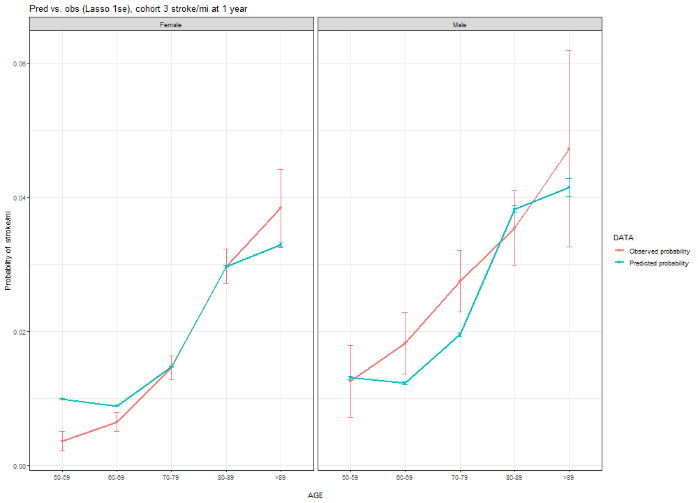


## Two-year MI/stroke prediction in cohorts OST, IFX and OBP from left to right


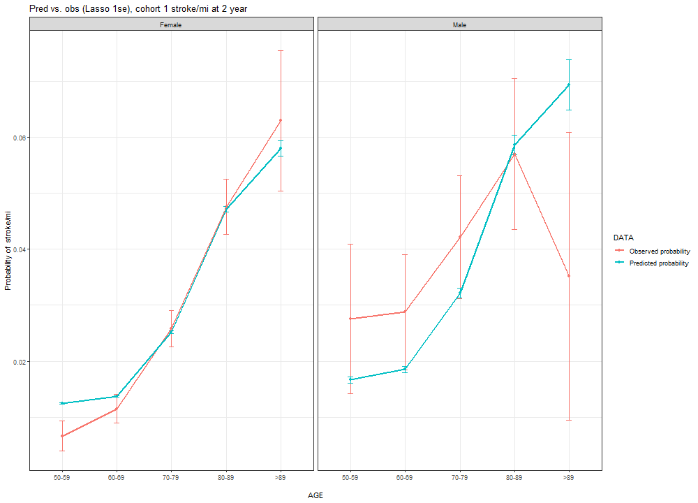

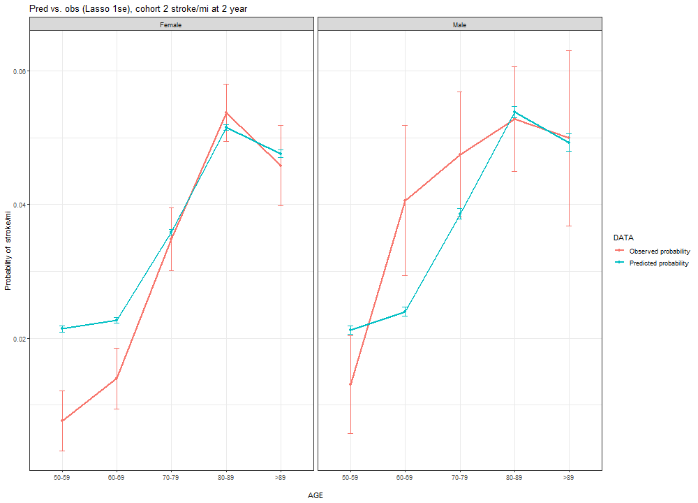

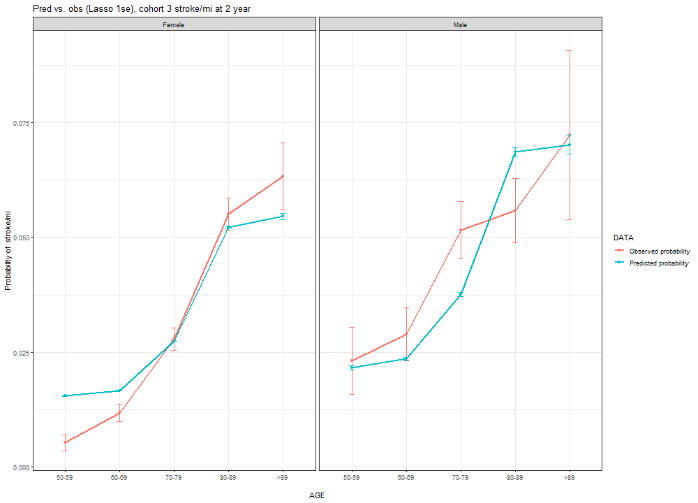

Supplement: Supplementary file 2 — Supplemental Fig. S1. Steps in the development and validation of the prediction model. Supplemental Fig. S2. Incidence rates by age groups. OST = patients with incident diagnosis of osteoporosis; IFX = patients with incident fragility fracture; OBP = incident users of oral bisphosphonates; MACE = composite outcome for the occurrence of either myocardial infarction, stroke, or cardiovascular disease death; MI = myocardial infarction. Supplemental Fig. S3. Incidence rates of 2‐year MACE, 1‐ and 2‐year stroke/MI; and MACE and stroke/MI by sex. OST = patients with incident diagnosis of osteoporosis; IFX = patients with incident fragility fracture; OBP = incident users of oral bisphosphonates; MACE = composite outcome for the occurrence of either myocardial infarction, stroke, or cardiovascular disease death; MI = myocardial infarction. Supplemental Fig. S4. Area under ROC curve for internal validation of 1‐ and 2‐year MACE outcome using risk factors from QRISK and LASSO models. OST = patients with incident diagnosis of osteoporosis; IFX, patients with incident fragility fracture; OBP, incident users of oral bisphosphonates; AUC, area under the curve; MACE, composite outcome for the occurrence of either myocardial infarction, stroke or cardiovascular disease death; MI, myocardial infarction. Supplemental Fig. S5. Area under ROC curve for internal validation of Stroke/MI outcome, and MACE and Stroke/MI sex‐based models. OST = patients with incident diagnosis of osteoporosis; IFX = patients with incident fragility fracture; OBP = incident users of oral bisphosphonates; MACE = composite outcome for the occurrence of either myocardial infarction, stroke or cardiovascular disease death; MI = myocardial infarction. Supplemental Fig. S6. Calibration curves for internal validation of 1‐year MACE prediction by age deciles. Models using risk factors selected by lasso regression. From left to right: OST, IFX, and OBP cohorts. OST = patients with incident diagnosis of osteoporosis; [file JBMR-37-1986-s002.docx]
