# Supplementary material for: Estimating the Incidence and Key Risk Factors of Cardiovascular Disease in Patients at High Risk of Imminent Fracture Using Routinely Collected Real‐World Data From the UK
Source: J Bone Miner Res. 2022 Sep 8;37(10):1986–96. doi: 10.1002/jbmr.4648 (PMC9826104; doi:10.1002/jbmr.4648)
Supplement: Supplementary file 3 — Supplemental Table S1. List of Available Variables Included in QRISK Tool Supplemental Table S2a. Data Set Split Into Train and Test Sets, Stratified by Outcome (OST Cohort) Supplemental Table S2b. Data Set Split Into Train and Test Sets, Stratified by Outcome (IFX Cohort) Supplemental Table S2c. Data Set Split Into Train and Test Sets, Stratified by Outcome (OBP Cohort) Supplemental Table S3. Model Equations of 1‐Year MACE (Risk Factors Selected by Lasso Regression) Supplemental Table S4a. Predictors of 2‐Year MACE Models (Risk Factors Selected by Lasso Regression) Supplemental Table S4b. Predictors of 1‐ and 2‐Year MI/Stroke Models (Risk Factors Selected by Lasso Regression) Supplemental Table S5a. Model Equations for 2‐Year MACE Models (Risk Factors Selected by Lasso Regression) Supplemental Table S5b. Model Equations for 1‐ and 2‐Year MI/Stroke Models (Risk Factors Selected by Lasso Regression) Supplemental Table S6a. Risk Factors Selected by Lasso for 1‐ and 2‐Year Models in Sex‐Based Models (OST Cohort) Supplemental Table S6b. Risk Factors Selected by Lasso for 1‐ and 2‐Year Models in Sex‐Based Models (IFX Cohort) Supplemental Table S6c. Risk Risk Factors Selected by Lasso for 1‐ and 2‐Year Models in Sex‐Based Models (OBP Cohort) Supplemental Table S7a. Model Equations From Lasso Selection for Women‐ and Men‐Based Models (OST cohort) Supplemental Table S7b. Model Equations From Lasso Selection for Women‐ and Men‐Based Models (IFX Cohort) Supplemental Table S7c. Model Equations From Lasso Selection for Women‐ and Men‐Based Models (OBP Cohort) [file JBMR-37-1986-s001.docx]

# Table S1 List of available variables included in QRISK tool

| Socio-demographic | Lab measurements | Comorbidities | Cardiovascular disease history | Drug use |
| --- | --- | --- | --- | --- |
| - Sex - Ethnicity - Age - BMI** - Smoking** - **Deprivation (Town score)** | - SBP** - Standardised SBP** - Cholesterol measurement** | - Rheumatoid arthritis* - Lupus* - Severe mental illness* - Diabetes type I* - Diabetes type II* - Chronic kidney disease* - Migraine* - Erectile dysfunction** | - Atrial fibrillation* - Family history of cardiovascular disease before age 60 | - On bisphosphonate use - Steroid use** - Antipsychotic use** |
| * Ever. ** In the year prior to start. In **bolt**, unavailable in CPRD. **Abbreviations**: BMI, body mass index; SPB, systolic blood pressure. | | | | |

# Table S2a Dataset split into train and test sets, stratified by outcome (OST cohort)

| **OST cohort** | **One year** | | | | | | | | **Two years** | | | | | | | |
| --- | --- | --- | --- | --- | --- | --- | --- | --- | --- | --- | --- | --- | --- | --- | --- | --- |
|  | **MACE** | | | | **Stroke/MI** | | | | **MACE** | | | | **Stroke/MI** | | | |
|  | **Development set (n=32648)** | | **Internal validation set (n=32647)** | | **Development set (n=32648)** | | **Internal validation set (n=32647)** | | **Development set (n=32648)** | | **Internal validation set (n=32647)** | | **Development set (n=32648)** | | **Internal validation set (n=32647)** | |
|  | **Outcome=NO** | **Outcome=YES** | **Outcome=NO** | **Outcome=YES** | **Outcome=NO** | **Outcome=YES** | **Outcome=NO** | **Outcome=YES** | **Outcome=NO** | **Outcome=YES** | **Outcome=NO** | **Outcome=YES** | **Outcome=NO** | **Outcome=YES** | **Outcome=NO** | **Outcome=YES** |
| n | 32029 | 619 | 32028 | 619 | 32135 | 513 | 32134 | 513 | 31492 | 1156 | 31492 | 1155 | 31701 | 947 | 31700 | 947 |
| Sex = Male (%) | 4186 (13.1) | 126 (20.4) | 4179 (13.0) | 125 (20.2) | 4283 (13.3) | 105 (20.5) | 4132 (12.9) | 96 (18.7) | 4051 (12.9) | 211 (18.3) | 4116 (13.1) | 238 (20.6) | 4235 (13.4) | 173 (18.3) | 4037 (12.7) | 171 (18.1) |
| SES (%) |  |  |  |  |  |  |  |  |  |  |  |  |  |  |  |  |
| 1 | 7904 (24.7) | 137 (22.1) | 7787 (24.3) | 125 (20.2) | 7833 (24.4) | 108 (21.1) | 7914 (24.6) | 98 (19.1) | 7743 (24.6) | 250 (21.6) | 7717 (24.5) | 243 (21.0) | 7783 (24.6) | 199 (21.0) | 7773 (24.5) | 198 (20.9) |
| 2 | 7655 (23.9) | 142 (22.9) | 7691 (24.0) | 155 (25.0) | 7645 (23.8) | 130 (25.3) | 7745 (24.1) | 123 (24.0) | 7590 (24.1) | 288 (24.9) | 7515 (23.9) | 250 (21.6) | 7662 (24.2) | 221 (23.3) | 7539 (23.8) | 221 (23.3) |
| 3 | 6749 (21.1) | 133 (21.5) | 6775 (21.2) | 137 (22.1) | 6814 (21.2) | 104 (20.3) | 6765 (21.1) | 111 (21.6) | 6615 (21.0) | 260 (22.5) | 6658 (21.1) | 261 (22.6) | 6628 (20.9) | 215 (22.7) | 6761 (21.3) | 190 (20.1) |
| 4 | 5855 (18.3) | 118 (19.1) | 5848 (18.3) | 100 (16.2) | 5889 (18.3) | 89 (17.3) | 5843 (18.2) | 100 (19.5) | 5766 (18.3) | 197 (17.0) | 5745 (18.2) | 213 (18.4) | 5802 (18.3) | 172 (18.2) | 5765 (18.2) | 182 (19.2) |
| 5 | 3827 (11.9) | 89 (14.4) | 3902 (12.2) | 100 (16.2) | 3929 (12.2) | 82 (16.0) | 3827 (11.9) | 80 (15.6) | 3738 (11.9) | 160 (13.8) | 3834 (12.2) | 186 (16.1) | 3797 (12.0) | 139 (14.7) | 3827 (12.1) | 155 (16.4) |
| Smoking** (%) |  |  |  |  |  |  |  |  |  |  |  |  |  |  |  |  |
| Ex | 10037 (31.3) | 199 (32.1) | 10062 (31.4) | 189 (30.5) | 10081 (31.4) | 169 (32.9) | 10075 (31.4) | 162 (31.6) | 9889 (31.4) | 385 (33.3) | 9839 (31.2) | 374 (32.4) | 9941 (31.4) | 290 (30.6) | 9929 (31.3) | 327 (34.5) |
| No | 16758 (52.3) | 314 (50.7) | 16661 (52.0) | 316 (51.1) | 16750 (52.1) | 259 (50.5) | 16771 (52.2) | 269 (52.4) | 16418 (52.1) | 597 (51.6) | 16447 (52.2) | 587 (50.8) | 16531 (52.1) | 515 (54.4) | 16538 (52.2) | 465 (49.1) |
| Yes | 5234 (16.3) | 106 (17.1) | 5305 (16.6) | 114 (18.4) | 5304 (16.5) | 85 (16.6) | 5288 (16.5) | 82 (16.0) | 5185 (16.5) | 174 (15.1) | 5206 (16.5) | 194 (16.8) | 5229 (16.5) | 142 (15.0) | 5233 (16.5) | 155 (16.4) |
| Drinking** (%) |  |  |  |  |  |  |  |  |  |  |  |  |  |  |  |  |
| Ex | 1702 (5.3) | 53 (8.6) | 1756 (5.5) | 53 (8.6) | 1741 (5.4) | 47 (9.2) | 1741 (5.4) | 35 (6.8) | 1708 (5.4) | 92 (8.0) | 1677 (5.3) | 87 (7.5) | 1699 (5.4) | 71 (7.5) | 1729 (5.5) | 65 (6.9) |
| No | 8244 (25.7) | 225 (36.3) | 8388 (26.2) | 205 (33.1) | 8364 (26.0) | 178 (34.7) | 8340 (26.0) | 180 (35.1) | 8175 (26.0) | 384 (33.2) | 8121 (25.8) | 382 (33.1) | 8262 (26.1) | 317 (33.5) | 8170 (25.8) | 313 (33.1) |
| Yes | 22083 (68.9) | 341 (55.1) | 21884 (68.3) | 361 (58.3) | 22030 (68.6) | 288 (56.1) | 22053 (68.6) | 298 (58.1) | 21609 (68.6) | 680 (58.8) | 21694 (68.9) | 686 (59.4) | 21740 (68.6) | 559 (59.0) | 21801 (68.8) | 569 (60.1) |
| Diabetes type I*= 1 (%) | 62 (0.2) | 2 (0.3) | 82 (0.3) | 6 (1.0) | 81 (0.3) | 2 (0.4) | 64 (0.2) | 5 (1.0) | 80 (0.3) | 3 (0.3) | 61 (0.2) | 8 (0.7) | 80 (0.3) | 4 (0.4) | 63 (0.2) | 5 (0.5) |
| Diabetes type II*= 1 (%) | 1632 (5.1) | 50 (8.1) | 1580 (4.9) | 49 (7.9) | 1634 (5.1) | 37 (7.2) | 1598 (5.0) | 42 (8.2) | 1554 (4.9) | 95 (8.2) | 1575 (5.0) | 87 (7.5) | 1583 (5.0) | 73 (7.7) | 1571 (5.0) | 84 (8.9) |
| Chronic obstructive pulmonary disease*= 1 (%) | 2332 (7.3) | 54 (8.7) | 2488 (7.8) | 65 (10.5) | 2477 (7.7) | 52 (10.1) | 2364 (7.4) | 46 (9.0) | 2368 (7.5) | 106 (9.2) | 2338 (7.4) | 127 (11.0) | 2338 (7.4) | 83 (8.8) | 2406 (7.6) | 112 (11.8) |
| Chronic kidney disease*= 1 (%) | 2781 (8.7) | 106 (17.1) | 2823 (8.8) | 80 (12.9) | 2810 (8.7) | 72 (14.0) | 2822 (8.8) | 86 (16.8) | 2791 (8.9) | 171 (14.8) | 2651 (8.4) | 177 (15.3) | 2738 (8.6) | 147 (15.5) | 2761 (8.7) | 144 (15.2) |
| Rheumatoid arthritis*= 1 (%) | 2424 (7.6) | 59 (9.5) | 2495 (7.8) | 67 (10.8) | 2452 (7.6) | 53 (10.3) | 2481 (7.7) | 59 (11.5) | 2452 (7.8) | 112 (9.7) | 2371 (7.5) | 110 (9.5) | 2412 (7.6) | 95 (10.0) | 2442 (7.7) | 96 (10.1) |
| Lupus*= 1 (%) | 69 (0.2) | 2 (0.3) | 73 (0.2) | 0 (0.0) | 65 (0.2) | 1 (0.2) | 78 (0.2) | 0 (0.0) | 77 (0.2) | 1 (0.1) | 65 (0.2) | 1 (0.1) | 73 (0.2) | 1 (0.1) | 68 (0.2) | 2 (0.2) |
| Systemic heart disease**= 1 (%) | 1154 (3.6) | 10 (1.6) | 1168 (3.6) | 9 (1.5) | 1146 (3.6) | 9 (1.8) | 1178 (3.7) | 8 (1.6) | 1192 (3.8) | 18 (1.6) | 1115 (3.5) | 16 (1.4) | 1110 (3.5) | 16 (1.7) | 1198 (3.8) | 17 (1.8) |
| Anti-osteoporosis use**= 1 (%) | 5671 (17.7) | 125 (20.2) | 5825 (18.2) | 131 (21.2) | 5766 (17.9) | 95 (18.5) | 5781 (18.0) | 110 (21.4) | 5633 (17.9) | 225 (19.5) | 5645 (17.9) | 249 (21.6) | 5715 (18.0) | 202 (21.3) | 5658 (17.8) | 177 (18.7) |
| Heparin use**= 1 (%) | 163 (0.5) | 3 (0.5) | 198 (0.6) | 8 (1.3) | 195 (0.6) | 3 (0.6) | 166 (0.5) | 8 (1.6) | 168 (0.5) | 11 (1.0) | 181 (0.6) | 12 (1.0) | 178 (0.6) | 13 (1.4) | 173 (0.5) | 8 (0.8) |
| Beta-blocker use**= 1 (%) | 5256 (16.4) | 154 (24.9) | 5016 (15.7) | 138 (22.3) | 5119 (15.9) | 123 (24.0) | 5180 (16.1) | 142 (27.7) | 5045 (16.0) | 286 (24.7) | 4966 (15.8) | 267 (23.1) | 5021 (15.8) | 262 (27.7) | 5049 (15.9) | 232 (24.5) |
| Hypertension**= 1 (%) | 2504 (7.8) | 69 (11.1) | 2464 (7.7) | 54 (8.7) | 2493 (7.8) | 60 (11.7) | 2485 (7.7) | 53 (10.3) | 2415 (7.7) | 105 (9.1) | 2443 (7.8) | 128 (11.1) | 2466 (7.8) | 97 (10.2) | 2428 (7.7) | 100 (10.6) |
| Deep vein thrombosis or pulmonary embolism**= 1 (%) | 257 (0.8) | 6 (1.0) | 244 (0.8) | 15 (2.4) | 267 (0.8) | 10 (1.9) | 238 (0.7) | 7 (1.4) | 249 (0.8) | 12 (1.0) | 245 (0.8) | 16 (1.4) | 244 (0.8) | 12 (1.3) | 254 (0.8) | 12 (1.3) |
| Anticoagulant use**= 1 (%) | 1578 (4.9) | 52 (8.4) | 1602 (5.0) | 53 (8.6) | 1609 (5.0) | 31 (6.0) | 1597 (5.0) | 48 (9.4) | 1576 (5.0) | 95 (8.2) | 1520 (4.8) | 94 (8.1) | 1554 (4.9) | 73 (7.7) | 1581 (5.0) | 77 (8.1) |
| Antidepressants TCA**= 1 (%) | 3418 (10.7) | 82 (13.2) | 3468 (10.8) | 71 (11.5) | 3419 (10.6) | 69 (13.5) | 3482 (10.8) | 69 (13.5) | 3361 (10.7) | 135 (11.7) | 3406 (10.8) | 137 (11.9) | 3400 (10.7) | 109 (11.5) | 3402 (10.7) | 128 (13.5) |
| Antidepressants SSRI**= 1 (%) | 2900 (9.1) | 84 (13.6) | 2928 (9.1) | 78 (12.6) | 2920 (9.1) | 68 (13.3) | 2942 (9.2) | 60 (11.7) | 2886 (9.2) | 146 (12.6) | 2808 (8.9) | 150 (13.0) | 2855 (9.0) | 116 (12.2) | 2903 (9.2) | 116 (12.2) |
| Hypercholesterolemia**= 1 (%) | 793 (2.5) | 16 (2.6) | 740 (2.3) | 12 (1.9) | 740 (2.3) | 16 (3.1) | 797 (2.5) | 8 (1.6) | 733 (2.3) | 28 (2.4) | 776 (2.5) | 24 (2.1) | 767 (2.4) | 13 (1.4) | 750 (2.4) | 31 (3.3) |
| Statin use**= 1 (%) | 7643 (23.9) | 196 (31.7) | 7542 (23.5) | 190 (30.7) | 7634 (23.8) | 165 (32.2) | 7602 (23.7) | 170 (33.1) | 7419 (23.6) | 347 (30.0) | 7434 (23.6) | 371 (32.1) | 7557 (23.8) | 294 (31.0) | 7402 (23.4) | 318 (33.6) |
| Family history of cardiovascular disease = 1 (%) | 3310 (10.3) | 55 (8.9) | 3343 (10.4) | 47 (7.6) | 3316 (10.3) | 42 (8.2) | 3343 (10.4) | 54 (10.5) | 3329 (10.6) | 94 (8.1) | 3234 (10.3) | 98 (8.5) | 3358 (10.6) | 87 (9.2) | 3213 (10.1) | 97 (10.2) |
| Family history of cardiovascular disease before age 60= 1 (%) | 46 (0.1) | 1 (0.2) | 52 (0.2) | 0 (0.0) | 59 (0.2) | 1 (0.2) | 39 (0.1) | 0 (0.0) | 48 (0.2) | 0 (0.0) | 50 (0.2) | 1 (0.1) | 54 (0.2) | 0 (0.0) | 44 (0.1) | 1 (0.1) |
| Heart failure*= 1 (%) | 1089 (3.4) | 41 (6.6) | 1127 (3.5) | 66 (10.7) | 1116 (3.5) | 45 (8.8) | 1118 (3.5) | 44 (8.6) | 1107 (3.5) | 95 (8.2) | 1030 (3.3) | 91 (7.9) | 1051 (3.3) | 75 (7.9) | 1124 (3.5) | 73 (7.7) |
| Migraine*= 1 (%) | 5040 (15.7) | 91 (14.7) | 4878 (15.2) | 94 (15.2) | 4921 (15.3) | 80 (15.6) | 5011 (15.6) | 91 (17.7) | 4862 (15.4) | 164 (14.2) | 4882 (15.5) | 195 (16.9) | 4803 (15.2) | 166 (17.5) | 4979 (15.7) | 155 (16.4) |
| Severe mental illness*= 1 (%) | 4890 (15.3) | 104 (16.8) | 5110 (16.0) | 99 (16.0) | 4940 (15.4) | 79 (15.4) | 5093 (15.8) | 91 (17.7) | 4983 (15.8) | 187 (16.2) | 4827 (15.3) | 206 (17.8) | 4931 (15.6) | 165 (17.4) | 4945 (15.6) | 162 (17.1) |
| Vascular Disease*= 1 (%) | 363 (1.1) | 16 (2.6) | 437 (1.4) | 23 (3.7) | 413 (1.3) | 19 (3.7) | 389 (1.2) | 18 (3.5) | 393 (1.2) | 39 (3.4) | 376 (1.2) | 31 (2.7) | 390 (1.2) | 32 (3.4) | 380 (1.2) | 37 (3.9) |
| Atrial fibrillation*= 1 (%) | 1788 (5.6) | 85 (13.7) | 1708 (5.3) | 83 (13.4) | 1764 (5.5) | 64 (12.5) | 1766 (5.5) | 70 (13.6) | 1707 (5.4) | 134 (11.6) | 1676 (5.3) | 147 (12.7) | 1710 (5.4) | 119 (12.6) | 1730 (5.5) | 105 (11.1) |
| On anti-hypertensive drug= 1 (%) | 18216 (56.9) | 464 (75.0) | 17934 (56.0) | 439 (70.9) | 18154 (56.5) | 381 (74.3) | 18131 (56.4) | 387 (75.4) | 17812 (56.6) | 829 (71.7) | 17545 (55.7) | 867 (75.1) | 17790 (56.1) | 730 (77.1) | 17847 (56.3) | 686 (72.4) |
| Antipsychotic use**= 1 (%) | 167 (0.5) | 4 (0.6) | 197 (0.6) | 3 (0.5) | 183 (0.6) | 2 (0.4) | 184 (0.6) | 2 (0.4) | 174 (0.6) | 9 (0.8) | 181 (0.6) | 7 (0.6) | 174 (0.5) | 6 (0.6) | 187 (0.6) | 4 (0.4) |
| Steroid use**= 1 (%) | 4494 (14.0) | 111 (17.9) | 4652 (14.5) | 110 (17.8) | 4624 (14.4) | 86 (16.8) | 4564 (14.2) | 93 (18.1) | 4534 (14.4) | 190 (16.4) | 4417 (14.0) | 226 (19.6) | 4457 (14.1) | 160 (16.9) | 4568 (14.4) | 182 (19.2) |
| Erectile dysfunction**= 1 (%) | 413 (1.3) | 11 (1.8) | 434 (1.4) | 13 (2.1) | 407 (1.3) | 7 (1.4) | 442 (1.4) | 15 (2.9) | 428 (1.4) | 20 (1.7) | 397 (1.3) | 26 (2.3) | 426 (1.3) | 13 (1.4) | 406 (1.3) | 26 (2.7) |
| Age>75 | 15094 (47.1) | 502 (81.1) | 15030 (46.9) | 479 (77.4) | 15174 (47.2) | 396 (77.2) | 15136 (47.1) | 399 (77.8) | 14674 (46.6) | 896 (77.5) | 14632 (46.5) | 903 (78.2) | 14786 (46.6) | 740 (78.1) | 14879 (46.9) | 700 (73.9) |
| Age Group (%) |  |  |  |  |  |  |  |  |  |  |  |  |  |  |  |  |
| 50-59 | 4143 (12.9) | 18 (2.9) | 4133 (12.9) | 21 (3.4) | 4167 (13.0) | 22 (4.3) | 4112 (12.8) | 14 (2.7) | 4131 (13.1) | 34 (2.9) | 4120 (13.1) | 30 (2.6) | 4095 (12.9) | 19 (2.0) | 4161 (13.1) | 40 (4.2) |
| 60-69 | 7821 (24.4) | 51 (8.2) | 8015 (25.0) | 62 (10.0) | 7906 (24.6) | 43 (8.4) | 7939 (24.7) | 61 (11.9) | 7797 (24.8) | 109 (9.4) | 7926 (25.2) | 117 (10.1) | 7892 (24.9) | 97 (10.2) | 7851 (24.8) | 109 (11.5) |
| 70-79 | 10501 (32.8) | 172 (27.8) | 10315 (32.2) | 177 (28.6) | 10412 (32.4) | 150 (29.2) | 10455 (32.5) | 148 (28.8) | 10215 (32.4) | 351 (30.4) | 10265 (32.6) | 334 (28.9) | 10396 (32.8) | 297 (31.4) | 10181 (32.1) | 291 (30.7) |
| 80-89 | 8028 (25.1) | 298 (48.1) | 7995 (25.0) | 265 (42.8) | 8094 (25.2) | 223 (43.5) | 8037 (25.0) | 232 (45.2) | 7856 (24.9) | 501 (43.3) | 7706 (24.5) | 523 (45.3) | 7798 (24.6) | 418 (44.1) | 7961 (25.1) | 409 (43.2) |
| >89 | 1536 (4.8) | 80 (12.9) | 1570 (4.9) | 94 (15.2) | 1556 (4.8) | 75 (14.6) | 1591 (5.0) | 58 (11.3) | 1493 (4.7) | 161 (13.9) | 1475 (4.7) | 151 (13.1) | 1520 (4.8) | 116 (12.2) | 1546 (4.9) | 98 (10.3) |
| Charlson score (%) |  |  |  |  |  |  |  |  |  |  |  |  |  |  |  |  |
| 0 | 18763 (58.6) | 280 (45.2) | 18477 (57.7) | 262 (42.3) | 18732 (58.3) | 226 (44.1) | 18603 (57.9) | 221 (43.1) | 18315 (58.2) | 508 (43.9) | 18443 (58.6) | 516 (44.7) | 18495 (58.3) | 417 (44.0) | 18455 (58.2) | 415 (43.8) |
| 1 | 6472 (20.2) | 143 (23.1) | 6496 (20.3) | 163 (26.3) | 6454 (20.1) | 131 (25.5) | 6568 (20.4) | 121 (23.6) | 6349 (20.2) | 291 (25.2) | 6353 (20.2) | 281 (24.3) | 6356 (20.0) | 225 (23.8) | 6447 (20.3) | 246 (26.0) |
| 2 | 3794 (11.8) | 83 (13.4) | 3995 (12.5) | 88 (14.2) | 3871 (12.0) | 67 (13.1) | 3951 (12.3) | 71 (13.8) | 3830 (12.2) | 172 (14.9) | 3813 (12.1) | 145 (12.6) | 3865 (12.2) | 140 (14.8) | 3842 (12.1) | 113 (11.9) |
| ≥3 | 3000 (9.4) | 113 (18.3) | 3060 (9.6) | 106 (17.1) | 3078 (9.6) | 89 (17.3) | 3012 (9.4) | 100 (19.5) | 2998 (9.5) | 185 (16.0) | 2883 (9.2) | 213 (18.4) | 2985 (9.4) | 165 (17.4) | 2956 (9.3) | 173 (18.3) |
| Cardiovascular disease (%) |  |  |  |  |  |  |  |  |  |  |  |  |  |  |  |  |
| No | 28476 (88.9) | 488 (78.8) | 28594 (89.3) | 476 (76.9) | 28578 (88.9) | 389 (75.8) | 28675 (89.2) | 392 (76.4) | 28079 (89.2) | 911 (78.8) | 28138 (89.3) | 906 (78.4) | 28290 (89.2) | 735 (77.6) | 28283 (89.2) | 726 (76.7) |
| Ever >1 year before index date | 2743 (8.6) | 90 (14.5) | 2687 (8.4) | 96 (15.5) | 2755 (8.6) | 83 (16.2) | 2690 (8.4) | 88 (17.2) | 2638 (8.4) | 170 (14.7) | 2628 (8.3) | 180 (15.6) | 2639 (8.3) | 145 (15.3) | 2664 (8.4) | 168 (17.7) |
| 1 year before index | 358 (1.1) | 15 (2.4) | 326 (1.0) | 20 (3.2) | 328 (1.0) | 14 (2.7) | 363 (1.1) | 14 (2.7) | 358 (1.1) | 30 (2.6) | 306 (1.0) | 25 (2.2) | 341 (1.1) | 19 (2.0) | 334 (1.1) | 25 (2.6) |
| 6 months before index | 360 (1.1) | 17 (2.7) | 322 (1.0) | 24 (3.9) | 361 (1.1) | 22 (4.3) | 324 (1.0) | 16 (3.1) | 331 (1.1) | 30 (2.6) | 324 (1.0) | 38 (3.3) | 342 (1.1) | 37 (3.9) | 319 (1.0) | 25 (2.6) |
| 1 month before index | 92 (0.3) | 9 (1.5) | 99 (0.3) | 3 (0.5) | 113 (0.4) | 5 (1.0) | 82 (0.3) | 3 (0.6) | 86 (0.3) | 15 (1.3) | 96 (0.3) | 6 (0.5) | 89 (0.3) | 11 (1.2) | 100 (0.3) | 3 (0.3) |
| MI or Stroke (%) |  |  |  |  |  |  |  |  |  |  |  |  |  |  |  |  |
| No | 30118 (94.0) | 499 (80.6) | 30236 (94.4) | 497 (80.3) | 30230 (94.1) | 409 (79.7) | 30279 (94.2) | 432 (84.2) | 29774 (94.5) | 970 (83.9) | 29670 (94.2) | 936 (81.0) | 29899 (94.3) | 798 (84.3) | 29863 (94.2) | 790 (83.4) |
| Ever >1 year before index date | 1380 (4.3) | 69 (11.1) | 1325 (4.1) | 76 (12.3) | 1373 (4.3) | 65 (12.7) | 1362 (4.2) | 50 (9.7) | 1245 (4.0) | 111 (9.6) | 1350 (4.3) | 144 (12.5) | 1308 (4.1) | 97 (10.2) | 1341 (4.2) | 104 (11.0) |
| 1 year before index | 531 (1.7) | 51 (8.2) | 467 (1.5) | 46 (7.4) | 532 (1.7) | 39 (7.6) | 493 (1.5) | 31 (6.0) | 473 (1.5) | 75 (6.5) | 472 (1.5) | 75 (6.5) | 494 (1.6) | 52 (5.5) | 496 (1.6) | 53 (5.6) |
| Established CVD *= Ever (%) | 3557 (11.1) | 187 (30.2) | 3495 (10.9) | 189 (30.5) | 3573 (11.1) | 161 (31.4) | 3545 (11.0) | 149 (29.0) | 3373 (10.7) | 314 (27.2) | 3404 (10.8) | 337 (29.2) | 3474 (11.0) | 272 (28.7) | 3423 (10.8) | 259 (27.3) |
| Any fracture history (%) |  |  |  |  |  |  |  |  |  |  |  |  |  |  |  |  |
| No | 24236 (75.7) | 449 (72.5) | 24408 (76.2) | 449 (72.5) | 24318 (75.7) | 369 (71.9) | 24490 (76.2) | 365 (71.2) | 23910 (75.9) | 867 (75.0) | 23930 (76.0) | 835 (72.3) | 24073 (75.9) | 690 (72.9) | 24089 (76.0) | 690 (72.9) |
| Ever >1 year before index date | 2899 (9.1) | 71 (11.5) | 2788 (8.7) | 55 (8.9) | 2934 (9.1) | 58 (11.3) | 2762 (8.6) | 59 (11.5) | 2780 (8.8) | 102 (8.8) | 2802 (8.9) | 129 (11.2) | 2821 (8.9) | 101 (10.7) | 2788 (8.8) | 103 (10.9) |
| 1 year before index | 4894 (15.3) | 99 (16.0) | 4832 (15.1) | 115 (18.6) | 4883 (15.2) | 86 (16.8) | 4882 (15.2) | 89 (17.3) | 4802 (15.2) | 187 (16.2) | 4760 (15.1) | 191 (16.5) | 4807 (15.2) | 156 (16.5) | 4823 (15.2) | 154 (16.3) |
| Hip fracture history (%) |  |  |  |  |  |  |  |  |  |  |  |  |  |  |  |  |
| No | 30453 (95.1) | 575 (92.9) | 30443 (95.1) | 569 (91.9) | 30577 (95.2) | 473 (92.2) | 30512 (95.0) | 478 (93.2) | 29921 (95.0) | 1080 (93.4) | 29978 (95.2) | 1061 (91.9) | 30121 (95.0) | 891 (94.1) | 30161 (95.1) | 867 (91.6) |
| Ever >1 year before index date | 541 (1.7) | 15 (2.4) | 541 (1.7) | 21 (3.4) | 554 (1.7) | 18 (3.5) | 535 (1.7) | 11 (2.1) | 548 (1.7) | 30 (2.6) | 500 (1.6) | 40 (3.5) | 553 (1.7) | 23 (2.4) | 511 (1.6) | 31 (3.3) |
| 1 year before index | 1035 (3.2) | 29 (4.7) | 1044 (3.3) | 29 (4.7) | 1004 (3.1) | 22 (4.3) | 1087 (3.4) | 24 (4.7) | 1023 (3.2) | 46 (4.0) | 1014 (3.2) | 54 (4.7) | 1027 (3.2) | 33 (3.5) | 1028 (3.2) | 49 (5.2) |
| Shoulder fracture history (%) |  |  |  |  |  |  |  |  |  |  |  |  |  |  |  |  |
| No | 31803 (99.3) | 616 (99.5) | 31805 (99.3) | 612 (98.9) | 31909 (99.3) | 511 (99.6) | 31910 (99.3) | 506 (98.6) | 31265 (99.3) | 1149 (99.4) | 31276 (99.3) | 1146 (99.2) | 31478 (99.3) | 938 (99.0) | 31479 (99.3) | 941 (99.4) |
| Ever >1 year before index date | 124 (0.4) | 0 (0.0) | 107 (0.3) | 1 (0.2) | 115 (0.4) | 0 (0.0) | 117 (0.4) | 0 (0.0) | 120 (0.4) | 0 (0.0) | 109 (0.3) | 3 (0.3) | 115 (0.4) | 2 (0.2) | 115 (0.4) | 0 (0.0) |
| 1 year before index | 102 (0.3) | 3 (0.5) | 116 (0.4) | 6 (1.0) | 111 (0.3) | 2 (0.4) | 107 (0.3) | 7 (1.4) | 107 (0.3) | 7 (0.6) | 107 (0.3) | 6 (0.5) | 108 (0.3) | 7 (0.7) | 106 (0.3) | 6 (0.6) |
| Spine fracture history (%) |  |  |  |  |  |  |  |  |  |  |  |  |  |  |  |  |
| No | 31352 (97.9) | 601 (97.1) | 31328 (97.8) | 599 (96.8) | 31434 (97.8) | 498 (97.1) | 31454 (97.9) | 494 (96.3) | 30827 (97.9) | 1129 (97.7) | 30815 (97.9) | 1109 (96.0) | 31009 (97.8) | 908 (95.9) | 31039 (97.9) | 924 (97.6) |
| Ever >1 year before index date | 159 (0.5) | 7 (1.1) | 156 (0.5) | 4 (0.6) | 165 (0.5) | 5 (1.0) | 152 (0.5) | 4 (0.8) | 154 (0.5) | 7 (0.6) | 152 (0.5) | 13 (1.1) | 156 (0.5) | 10 (1.1) | 154 (0.5) | 6 (0.6) |
| 1 year before index | 518 (1.6) | 11 (1.8) | 544 (1.7) | 16 (2.6) | 536 (1.7) | 10 (1.9) | 528 (1.6) | 15 (2.9) | 511 (1.6) | 20 (1.7) | 525 (1.7) | 33 (2.9) | 536 (1.7) | 29 (3.1) | 507 (1.6) | 17 (1.8) |
| Wrist fracture history (%) |  |  |  |  |  |  |  |  |  |  |  |  |  |  |  |  |
| No | 29468 (92.0) | 579 (93.5) | 29620 (92.5) | 570 (92.1) | 29609 (92.1) | 471 (91.8) | 29680 (92.4) | 477 (93.0) | 29021 (92.2) | 1077 (93.2) | 29068 (92.3) | 1071 (92.7) | 29209 (92.1) | 870 (91.9) | 29277 (92.4) | 881 (93.0) |
| Ever >1 year before index date | 1183 (3.7) | 21 (3.4) | 1139 (3.6) | 20 (3.2) | 1216 (3.8) | 19 (3.7) | 1112 (3.5) | 16 (3.1) | 1132 (3.6) | 36 (3.1) | 1155 (3.7) | 40 (3.5) | 1183 (3.7) | 38 (4.0) | 1112 (3.5) | 30 (3.2) |
| 1 year before index | 1378 (4.3) | 19 (3.1) | 1269 (4.0) | 29 (4.7) | 1310 (4.1) | 23 (4.5) | 1342 (4.2) | 20 (3.9) | 1339 (4.3) | 43 (3.7) | 1269 (4.0) | 44 (3.8) | 1309 (4.1) | 39 (4.1) | 1311 (4.1) | 36 (3.8) |
| BMI** (%) |  |  |  |  |  |  |  |  |  |  |  |  |  |  |  |  |
| <18.5 | 2754 (8.6) | 93 (15.0) | 2691 (8.4) | 77 (12.4) | 2693 (8.4) | 63 (12.3) | 2796 (8.7) | 63 (12.3) | 2637 (8.4) | 156 (13.5) | 2672 (8.5) | 150 (13.0) | 2700 (8.5) | 121 (12.8) | 2681 (8.5) | 113 (11.9) |
| 18.6 - 24.9 | 16003 (50.0) | 310 (50.1) | 15903 (49.7) | 348 (56.2) | 15960 (49.7) | 277 (54.0) | 16069 (50.0) | 258 (50.3) | 15780 (50.1) | 608 (52.6) | 15575 (49.5) | 601 (52.0) | 15815 (49.9) | 482 (50.9) | 15786 (49.8) | 481 (50.8) |
| 25 - 29.9 | 8949 (27.9) | 153 (24.7) | 9110 (28.4) | 131 (21.2) | 9131 (28.4) | 128 (25.0) | 8963 (27.9) | 121 (23.6) | 8826 (28.0) | 269 (23.3) | 8964 (28.5) | 284 (24.6) | 8950 (28.2) | 252 (26.6) | 8917 (28.1) | 224 (23.7) |
| 30 - 39.9 | 4016 (12.5) | 57 (9.2) | 4033 (12.6) | 58 (9.4) | 4021 (12.5) | 40 (7.8) | 4038 (12.6) | 65 (12.7) | 3962 (12.6) | 114 (9.9) | 3982 (12.6) | 106 (9.2) | 3949 (12.5) | 84 (8.9) | 4015 (12.7) | 116 (12.2) |
| >=40 | 307 (1.0) | 6 (1.0) | 291 (0.9) | 5 (0.8) | 330 (1.0) | 5 (1.0) | 268 (0.8) | 6 (1.2) | 287 (0.9) | 9 (0.8) | 299 (0.9) | 14 (1.2) | 287 (0.9) | 8 (0.8) | 301 (0.9) | 13 (1.4) |
| No. of GP visits** (%) |  |  |  |  |  |  |  |  |  |  |  |  |  |  |  |  |
| 0 | 1555 (4.9) | 32 (5.2) | 1596 (5.0) | 36 (5.8) | 1593 (5.0) | 27 (5.3) | 1578 (4.9) | 21 (4.1) | 1509 (4.8) | 71 (6.1) | 1576 (5.0) | 63 (5.5) | 1520 (4.8) | 47 (5.0) | 1604 (5.1) | 48 (5.1) |
| 1-5 | 8001 (25.0) | 95 (15.3) | 7959 (24.9) | 121 (19.5) | 7916 (24.6) | 90 (17.5) | 8084 (25.2) | 86 (16.8) | 7797 (24.8) | 200 (17.3) | 7980 (25.3) | 199 (17.2) | 7964 (25.1) | 161 (17.0) | 7891 (24.9) | 160 (16.9) |
| 6-10 | 8588 (26.8) | 138 (22.3) | 8600 (26.9) | 126 (20.4) | 8694 (27.1) | 109 (21.2) | 8537 (26.6) | 112 (21.8) | 8507 (27.0) | 259 (22.4) | 8427 (26.8) | 259 (22.4) | 8570 (27.0) | 222 (23.4) | 8453 (26.7) | 207 (21.9) |
| 11-15 | 5798 (18.1) | 111 (17.9) | 5898 (18.4) | 131 (21.2) | 5865 (18.3) | 91 (17.7) | 5878 (18.3) | 104 (20.3) | 5792 (18.4) | 219 (18.9) | 5689 (18.1) | 238 (20.6) | 5782 (18.2) | 174 (18.4) | 5791 (18.3) | 191 (20.2) |
| >=16 | 8087 (25.2) | 243 (39.3) | 7975 (24.9) | 205 (33.1) | 8067 (25.1) | 196 (38.2) | 8057 (25.1) | 190 (37.0) | 7887 (25.0) | 407 (35.2) | 7820 (24.8) | 396 (34.3) | 7865 (24.8) | 343 (36.2) | 7961 (25.1) | 341 (36.0) |
| No. of GP emergency visits** (%) |  |  |  |  |  |  |  |  |  |  |  |  |  |  |  |  |
| 0 | 26075 (81.4) | 407 (65.8) | 26200 (81.8) | 413 (66.7) | 26216 (81.6) | 339 (66.1) | 26186 (81.5) | 354 (69.0) | 25664 (81.5) | 815 (70.5) | 25832 (82.0) | 784 (67.9) | 25837 (81.5) | 680 (71.8) | 25924 (81.8) | 654 (69.1) |
| 1 | 3237 (10.1) | 88 (14.2) | 3161 (9.9) | 84 (13.6) | 3217 (10.0) | 74 (14.4) | 3218 (10.0) | 61 (11.9) | 3188 (10.1) | 144 (12.5) | 3083 (9.8) | 155 (13.4) | 3198 (10.1) | 105 (11.1) | 3138 (9.9) | 129 (13.6) |
| 2 | 1174 (3.7) | 47 (7.6) | 1171 (3.7) | 47 (7.6) | 1159 (3.6) | 39 (7.6) | 1201 (3.7) | 40 (7.8) | 1162 (3.7) | 85 (7.4) | 1118 (3.6) | 74 (6.4) | 1151 (3.6) | 63 (6.7) | 1157 (3.6) | 68 (7.2) |
| 3-5 | 1107 (3.5) | 52 (8.4) | 1078 (3.4) | 45 (7.3) | 1095 (3.4) | 36 (7.0) | 1114 (3.5) | 37 (7.2) | 1081 (3.4) | 64 (5.5) | 1041 (3.3) | 96 (8.3) | 1121 (3.5) | 65 (6.9) | 1038 (3.3) | 58 (6.1) |
| >=6 | 436 (1.4) | 25 (4.0) | 418 (1.3) | 30 (4.8) | 448 (1.4) | 25 (4.9) | 415 (1.3) | 21 (4.1) | 397 (1.3) | 48 (4.2) | 418 (1.3) | 46 (4.0) | 394 (1.2) | 34 (3.6) | 443 (1.4) | 38 (4.0) |
| eGFR** (%) |  |  |  |  |  |  |  |  |  |  |  |  |  |  |  |  |
| <=29 | 309 (1.0) | 23 (3.7) | 359 (1.1) | 17 (2.7) | 339 (1.1) | 12 (2.3) | 331 (1.0) | 26 (5.1) | 342 (1.1) | 32 (2.8) | 308 (1.0) | 26 (2.3) | 344 (1.1) | 18 (1.9) | 313 (1.0) | 33 (3.5) |
| 30 - 44 | 1672 (5.2) | 71 (11.5) | 1664 (5.2) | 63 (10.2) | 1676 (5.2) | 66 (12.9) | 1662 (5.2) | 66 (12.9) | 1584 (5.0) | 121 (10.5) | 1624 (5.2) | 141 (12.2) | 1576 (5.0) | 122 (12.9) | 1670 (5.3) | 102 (10.8) |
| 45 - 59 | 5867 (18.3) | 170 (27.5) | 5760 (18.0) | 153 (24.7) | 5795 (18.0) | 141 (27.5) | 5876 (18.3) | 138 (26.9) | 5703 (18.1) | 281 (24.3) | 5659 (18.0) | 307 (26.6) | 5704 (18.0) | 239 (25.2) | 5762 (18.2) | 245 (25.9) |
| 60 - 89 | 22375 (69.9) | 325 (52.5) | 22430 (70.0) | 367 (59.3) | 22558 (70.2) | 273 (53.2) | 22400 (69.7) | 266 (51.9) | 22043 (70.0) | 680 (58.8) | 22155 (70.4) | 619 (53.6) | 22241 (70.2) | 523 (55.2) | 22211 (70.1) | 522 (55.1) |
| >=90 | 1806 (5.6) | 30 (4.8) | 1815 (5.7) | 19 (3.1) | 1767 (5.5) | 21 (4.1) | 1865 (5.8) | 17 (3.3) | 1820 (5.8) | 42 (3.6) | 1746 (5.5) | 62 (5.4) | 1836 (5.8) | 45 (4.8) | 1744 (5.5) | 45 (4.8) |
| SBP** (%) |  |  |  |  |  |  |  |  |  |  |  |  |  |  |  |  |
| <120 | 4464 (13.9) | 69 (11.1) | 4449 (13.9) | 94 (15.2) | 4476 (13.9) | 60 (11.7) | 4465 (13.9) | 75 (14.6) | 4440 (14.1) | 136 (11.8) | 4359 (13.8) | 141 (12.2) | 4434 (14.0) | 112 (11.8) | 4416 (13.9) | 114 (12.0) |
| 120 - 139 | 13104 (40.9) | 223 (36.0) | 13053 (40.8) | 225 (36.3) | 13108 (40.8) | 179 (34.9) | 13137 (40.9) | 181 (35.3) | 12877 (40.9) | 418 (36.2) | 12894 (40.9) | 416 (36.0) | 12927 (40.8) | 333 (35.2) | 12999 (41.0) | 346 (36.5) |
| 140 - 159 | 11063 (34.5) | 250 (40.4) | 11070 (34.6) | 205 (33.1) | 11106 (34.6) | 194 (37.8) | 11099 (34.5) | 189 (36.8) | 10810 (34.3) | 433 (37.5) | 10909 (34.6) | 436 (37.7) | 10937 (34.5) | 375 (39.6) | 10936 (34.5) | 340 (35.9) |
| >=160 | 3398 (10.6) | 77 (12.4) | 3456 (10.8) | 95 (15.3) | 3445 (10.7) | 80 (15.6) | 3433 (10.7) | 68 (13.3) | 3365 (10.7) | 169 (14.6) | 3330 (10.6) | 162 (14.0) | 3403 (10.7) | 127 (13.4) | 3349 (10.6) | 147 (15.5) |
| DBP** (%) |  |  |  |  |  |  |  |  |  |  |  |  |  |  |  |  |
| <80 | 16431 (51.3) | 350 (56.5) | 16616 (51.9) | 332 (53.6) | 16547 (51.5) | 281 (54.8) | 16617 (51.7) | 284 (55.4) | 16245 (51.6) | 620 (53.6) | 16251 (51.6) | 613 (53.1) | 16367 (51.6) | 505 (53.3) | 16352 (51.6) | 505 (53.3) |
| 80 - 89 | 12144 (37.9) | 208 (33.6) | 11934 (37.3) | 219 (35.4) | 12106 (37.7) | 175 (34.1) | 12049 (37.5) | 175 (34.1) | 11868 (37.7) | 424 (36.7) | 11795 (37.5) | 418 (36.2) | 11895 (37.5) | 349 (36.9) | 11919 (37.6) | 342 (36.1) |
| 90 - 99 | 2795 (8.7) | 50 (8.1) | 2868 (9.0) | 51 (8.2) | 2847 (8.9) | 45 (8.8) | 2829 (8.8) | 43 (8.4) | 2755 (8.7) | 90 (7.8) | 2822 (9.0) | 97 (8.4) | 2825 (8.9) | 77 (8.1) | 2786 (8.8) | 76 (8.0) |
| >=100 | 659 (2.1) | 11 (1.8) | 610 (1.9) | 17 (2.7) | 635 (2.0) | 12 (2.3) | 639 (2.0) | 11 (2.1) | 624 (2.0) | 22 (1.9) | 624 (2.0) | 27 (2.3) | 614 (1.9) | 16 (1.7) | 643 (2.0) | 24 (2.5) |
| No. of concomitant medicines** (%) |  |  |  |  |  |  |  |  |  |  |  |  |  |  |  |  |
| 0 | 2541 (7.9) | 38 (6.1) | 2548 (8.0) | 44 (7.1) | 2580 (8.0) | 35 (6.8) | 2532 (7.9) | 24 (4.7) | 2423 (7.7) | 81 (7.0) | 2601 (8.3) | 66 (5.7) | 2504 (7.9) | 50 (5.3) | 2563 (8.1) | 54 (5.7) |
| 1 - 3 | 6366 (19.9) | 51 (8.2) | 6380 (19.9) | 61 (9.9) | 6377 (19.8) | 49 (9.6) | 6382 (19.9) | 50 (9.7) | 6327 (20.1) | 115 (9.9) | 6301 (20.0) | 115 (10.0) | 6399 (20.2) | 109 (11.5) | 6259 (19.7) | 91 (9.6) |
| 4 - 6 | 7192 (22.5) | 103 (16.6) | 7060 (22.0) | 91 (14.7) | 7123 (22.2) | 69 (13.5) | 7168 (22.3) | 86 (16.8) | 7012 (22.3) | 200 (17.3) | 7047 (22.4) | 187 (16.2) | 7103 (22.4) | 147 (15.5) | 7032 (22.2) | 164 (17.3) |
| 7 - 9 | 6061 (18.9) | 121 (19.5) | 6052 (18.9) | 141 (22.8) | 5981 (18.6) | 116 (22.6) | 6177 (19.2) | 101 (19.7) | 5984 (19.0) | 239 (20.7) | 5919 (18.8) | 233 (20.2) | 5985 (18.9) | 194 (20.5) | 6007 (18.9) | 189 (20.0) |
| 10 - 12 | 4253 (13.3) | 119 (19.2) | 4326 (13.5) | 90 (14.5) | 4360 (13.6) | 86 (16.8) | 4260 (13.3) | 82 (16.0) | 4202 (13.3) | 201 (17.4) | 4179 (13.3) | 206 (17.8) | 4205 (13.3) | 178 (18.8) | 4252 (13.4) | 153 (16.2) |
| >=13 | 5616 (17.5) | 187 (30.2) | 5662 (17.7) | 192 (31.0) | 5714 (17.8) | 158 (30.8) | 5615 (17.5) | 170 (33.1) | 5544 (17.6) | 320 (27.7) | 5445 (17.3) | 348 (30.1) | 5505 (17.4) | 269 (28.4) | 5587 (17.6) | 296 (31.3) |
| Cholesterol measurement** (HDL/LDL) (%) |  |  |  |  |  |  |  |  |  |  |  |  |  |  |  |  |
| <=3.5 | 19256 (60.1) | 355 (57.4) | 19195 (59.9) | 337 (54.4) | 19260 (59.9) | 308 (60.0) | 19287 (60.0) | 288 (56.1) | 18799 (59.7) | 662 (57.3) | 18990 (60.3) | 692 (59.9) | 19074 (60.2) | 546 (57.7) | 18958 (59.8) | 565 (59.7) |
| 3.6 - 5 | 10175 (31.8) | 211 (34.1) | 10189 (31.8) | 217 (35.1) | 10227 (31.8) | 157 (30.6) | 10223 (31.8) | 185 (36.1) | 10164 (32.3) | 392 (33.9) | 9875 (31.4) | 361 (31.3) | 10081 (31.8) | 305 (32.2) | 10096 (31.8) | 310 (32.7) |
| >5 | 2598 (8.1) | 53 (8.6) | 2644 (8.3) | 65 (10.5) | 2648 (8.2) | 48 (9.4) | 2624 (8.2) | 40 (7.8) | 2529 (8.0) | 102 (8.8) | 2627 (8.3) | 102 (8.8) | 2546 (8.0) | 96 (10.1) | 2646 (8.3) | 72 (7.6) |
| No. of previous fractures* (%) |  |  |  |  |  |  |  |  |  |  |  |  |  |  |  |  |
| 0 | 22780 (71.1) | 404 (65.3) | 22957 (71.7) | 410 (66.2) | 22909 (71.3) | 339 (66.1) | 22979 (71.5) | 324 (63.2) | 22504 (71.5) | 799 (69.1) | 22495 (71.4) | 753 (65.2) | 22636 (71.4) | 616 (65.0) | 22660 (71.5) | 639 (67.5) |
| 1 | 4797 (15.0) | 129 (20.8) | 4730 (14.8) | 107 (17.3) | 4835 (15.0) | 99 (19.3) | 4724 (14.7) | 105 (20.5) | 4666 (14.8) | 198 (17.1) | 4693 (14.9) | 206 (17.8) | 4734 (14.9) | 179 (18.9) | 4686 (14.8) | 164 (17.3) |
| >=2 | 4452 (13.9) | 86 (13.9) | 4341 (13.6) | 102 (16.5) | 4391 (13.7) | 75 (14.6) | 4431 (13.8) | 84 (16.4) | 4322 (13.7) | 159 (13.8) | 4304 (13.7) | 196 (17.0) | 4331 (13.7) | 152 (16.1) | 4354 (13.7) | 144 (15.2) |
| **Abbreviations**: OST, patients with incident diagnosis of osteoporosis; IFX, patients with incident fragility fracture; OBP, incident users of oral bisphosphonates; MACE, composite outcome for the occurrence of either myocardial infarction, stroke or cardiovascular disease death; MI, myocardial infarction; * ever; ** in the year prior to start; SES, socio-economic status; BMI, body mass index; eGFR, estimated Glomerular Filtration Rate; SBP, cholesterol, systolic blood pressure; DBP, diastolic blood pressure. | | | | | | | | | | | | | | | | |

# Table S2b Dataset split into train and test sets, stratified by outcome (IFX cohort)

| **IFX cohort** | **One year** | | | | | | | | **Two years** | | | | | | | |
| --- | --- | --- | --- | --- | --- | --- | --- | --- | --- | --- | --- | --- | --- | --- | --- | --- |
|  | **MACE** | | | | **Stroke/MI** | | | | **MACE** | | | | **Stroke/MI** | | | |
|  | **Development set (n=33533)** | | **Internal validation set (n=33532)** | | **Development set (n=33533)** | | **Internal validation set (n=33532)** | | **Development set (n=33533)** | | **Internal validation set (n=33532)** | | **Development set (n=33533)** | | **Internal validation set (n=33532)** | |
|  | **Outcome=NO** | **Outcome=YES** | **Outcome=NO** | **Outcome=YES** | **Outcome=NO** | **Outcome=YES** | **Outcome=NO** | **Outcome=YES** | **Outcome=NO** | **Outcome=YES** | **Outcome=NO** | **Outcome=YES** | **Outcome=NO** | **Outcome=YES** | **Outcome=NO** | **Outcome=YES** |
| n | 32062 | 1471 | 32062 | 1470 | 32644 | 889 | 32643 | 889 | 31322 | 2211 | 31322 | 2210 | 32121 | 1412 | 32120 | 1412 |
| Sex = Male (%) | 7682 (24.0) | 438 (29.8) | 7853 (24.5) | 413 (28.1) | 7916 (24.2) | 228 (25.6) | 7998 (24.5) | 244 (27.4) | 7476 (23.9) | 612 (27.7) | 7692 (24.6) | 606 (27.4) | 7801 (24.3) | 354 (25.1) | 7861 (24.5) | 370 (26.2) |
| SES (%) |  |  |  |  |  |  |  |  |  |  |  |  |  |  |  |  |
| 1 | 7132 (22.2) | 300 (20.4) | 7246 (22.6) | 302 (20.5) | 7352 (22.5) | 183 (20.6) | 7281 (22.3) | 164 (18.4) | 6976 (22.3) | 459 (20.8) | 7066 (22.6) | 479 (21.7) | 7250 (22.6) | 276 (19.5) | 7156 (22.3) | 298 (21.1) |
| 2 | 7725 (24.1) | 345 (23.5) | 7558 (23.6) | 362 (24.6) | 7697 (23.6) | 205 (23.1) | 7872 (24.1) | 216 (24.3) | 7466 (23.8) | 509 (23.0) | 7475 (23.9) | 540 (24.4) | 7689 (23.9) | 314 (22.2) | 7647 (23.8) | 340 (24.1) |
| 3 | 6760 (21.1) | 310 (21.1) | 6942 (21.7) | 310 (21.1) | 6979 (21.4) | 182 (20.5) | 6962 (21.3) | 199 (22.4) | 6771 (21.6) | 473 (21.4) | 6629 (21.2) | 449 (20.3) | 6879 (21.4) | 304 (21.5) | 6843 (21.3) | 296 (21.0) |
| 4 | 6191 (19.3) | 300 (20.4) | 6123 (19.1) | 278 (18.9) | 6278 (19.2) | 180 (20.2) | 6259 (19.2) | 175 (19.7) | 5991 (19.1) | 432 (19.5) | 6039 (19.3) | 430 (19.5) | 6179 (19.2) | 276 (19.5) | 6151 (19.2) | 286 (20.3) |
| 5 | 4223 (13.2) | 212 (14.4) | 4169 (13.0) | 216 (14.7) | 4303 (13.2) | 137 (15.4) | 4247 (13.0) | 133 (15.0) | 4090 (13.1) | 335 (15.2) | 4087 (13.0) | 308 (13.9) | 4099 (12.8) | 238 (16.9) | 4292 (13.4) | 191 (13.5) |
| Smoking** (%) |  |  |  |  |  |  |  |  |  |  |  |  |  |  |  |  |
| Ex | 10305 (32.1) | 523 (35.6) | 10179 (31.7) | 477 (32.4) | 10564 (32.4) | 302 (34.0) | 10327 (31.6) | 291 (32.7) | 10035 (32.0) | 727 (32.9) | 9935 (31.7) | 787 (35.6) | 10176 (31.7) | 489 (34.6) | 10340 (32.2) | 479 (33.9) |
| No | 16775 (52.3) | 742 (50.4) | 16965 (52.9) | 790 (53.7) | 17136 (52.5) | 455 (51.2) | 17201 (52.7) | 480 (54.0) | 16448 (52.5) | 1120 (50.7) | 16578 (52.9) | 1126 (51.0) | 17037 (53.0) | 714 (50.6) | 16813 (52.3) | 708 (50.1) |
| Yes | 4982 (15.5) | 206 (14.0) | 4918 (15.3) | 203 (13.8) | 4944 (15.1) | 132 (14.8) | 5115 (15.7) | 118 (13.3) | 4839 (15.4) | 364 (16.5) | 4809 (15.4) | 297 (13.4) | 4908 (15.3) | 209 (14.8) | 4967 (15.5) | 225 (15.9) |
| Drinking** (%) |  |  |  |  |  |  |  |  |  |  |  |  |  |  |  |  |
| Ex | 1842 (5.7) | 76 (5.2) | 1905 (5.9) | 122 (8.3) | 1917 (5.9) | 64 (7.2) | 1903 (5.8) | 61 (6.9) | 1828 (5.8) | 140 (6.3) | 1832 (5.8) | 145 (6.6) | 1844 (5.7) | 92 (6.5) | 1913 (6.0) | 96 (6.8) |
| No | 10067 (31.4) | 551 (37.5) | 9970 (31.1) | 504 (34.3) | 10251 (31.4) | 304 (34.2) | 10211 (31.3) | 326 (36.7) | 9825 (31.4) | 754 (34.1) | 9756 (31.1) | 757 (34.3) | 10071 (31.4) | 475 (33.6) | 10092 (31.4) | 454 (32.2) |
| Yes | 20153 (62.9) | 844 (57.4) | 20187 (63.0) | 844 (57.4) | 20476 (62.7) | 521 (58.6) | 20529 (62.9) | 502 (56.5) | 19669 (62.8) | 1317 (59.6) | 19734 (63.0) | 1308 (59.2) | 20206 (62.9) | 845 (59.8) | 20115 (62.6) | 862 (61.0) |
| Diabetes type I*= 1 (%) | 84 (0.3) | 5 (0.3) | 68 (0.2) | 7 (0.5) | 76 (0.2) | 2 (0.2) | 80 (0.2) | 6 (0.7) | 66 (0.2) | 6 (0.3) | 84 (0.3) | 8 (0.4) | 81 (0.3) | 2 (0.1) | 75 (0.2) | 6 (0.4) |
| Diabetes type II*= 1 (%) | 1939 (6.0) | 104 (7.1) | 2005 (6.3) | 123 (8.4) | 2011 (6.2) | 68 (7.6) | 1998 (6.1) | 94 (10.6) | 1955 (6.2) | 161 (7.3) | 1895 (6.1) | 160 (7.2) | 1961 (6.1) | 119 (8.4) | 1969 (6.1) | 122 (8.6) |
| Chronic obstructive pulmonary disease*= 1 (%) | 1786 (5.6) | 86 (5.8) | 1775 (5.5) | 98 (6.7) | 1775 (5.4) | 55 (6.2) | 1860 (5.7) | 55 (6.2) | 1751 (5.6) | 134 (6.1) | 1738 (5.5) | 122 (5.5) | 1768 (5.5) | 94 (6.7) | 1809 (5.6) | 74 (5.2) |
| Chronic kidney disease*= 1 (%) | 3767 (11.7) | 261 (17.7) | 3833 (12.0) | 256 (17.4) | 3958 (12.1) | 150 (16.9) | 3856 (11.8) | 153 (17.2) | 3632 (11.6) | 372 (16.8) | 3750 (12.0) | 363 (16.4) | 3809 (11.9) | 220 (15.6) | 3849 (12.0) | 239 (16.9) |
| Rheumatoid arthritis*= 1 (%) | 1372 (4.3) | 78 (5.3) | 1385 (4.3) | 56 (3.8) | 1457 (4.5) | 45 (5.1) | 1341 (4.1) | 48 (5.4) | 1336 (4.3) | 113 (5.1) | 1349 (4.3) | 93 (4.2) | 1404 (4.4) | 62 (4.4) | 1346 (4.2) | 79 (5.6) |
| Lupus*= 1 (%) | 22 (0.1) | 3 (0.2) | 30 (0.1) | 0 (0.0) | 30 (0.1) | 1 (0.1) | 23 (0.1) | 1 (0.1) | 28 (0.1) | 4 (0.2) | 22 (0.1) | 1 (0.0) | 30 (0.1) | 2 (0.1) | 21 (0.1) | 2 (0.1) |
| Systemic heart disease**= 1 (%) | 269 (0.8) | 3 (0.2) | 290 (0.9) | 1 (0.1) | 266 (0.8) | 1 (0.1) | 294 (0.9) | 2 (0.2) | 285 (0.9) | 9 (0.4) | 263 (0.8) | 6 (0.3) | 269 (0.8) | 4 (0.3) | 281 (0.9) | 9 (0.6) |
| Anti-osteoporosis use**= 1 (%) | 2590 (8.1) | 126 (8.6) | 2523 (7.9) | 117 (8.0) | 2590 (7.9) | 78 (8.8) | 2603 (8.0) | 85 (9.6) | 2522 (8.1) | 165 (7.5) | 2467 (7.9) | 202 (9.1) | 2589 (8.1) | 123 (8.7) | 2517 (7.8) | 127 (9.0) |
| Heparin use**= 1 (%) | 125 (0.4) | 7 (0.5) | 147 (0.5) | 3 (0.2) | 145 (0.4) | 3 (0.3) | 129 (0.4) | 5 (0.6) | 141 (0.5) | 10 (0.5) | 123 (0.4) | 8 (0.4) | 140 (0.4) | 3 (0.2) | 130 (0.4) | 9 (0.6) |
| Beta-blocker use**= 1 (%) | 4856 (15.1) | 330 (22.4) | 4885 (15.2) | 321 (21.8) | 4955 (15.2) | 221 (24.9) | 5022 (15.4) | 194 (21.8) | 4772 (15.2) | 452 (20.4) | 4684 (15.0) | 484 (21.9) | 4931 (15.4) | 322 (22.8) | 4821 (15.0) | 318 (22.5) |
| Hypertension**= 1 (%) | 1585 (4.9) | 90 (6.1) | 1582 (4.9) | 77 (5.2) | 1555 (4.8) | 64 (7.2) | 1658 (5.1) | 57 (6.4) | 1544 (4.9) | 134 (6.1) | 1526 (4.9) | 130 (5.9) | 1580 (4.9) | 96 (6.8) | 1562 (4.9) | 96 (6.8) |
| Deep vein thrombosis or pulmonary embolism**= 1 (%) | 200 (0.6) | 12 (0.8) | 221 (0.7) | 6 (0.4) | 229 (0.7) | 7 (0.8) | 198 (0.6) | 5 (0.6) | 202 (0.6) | 12 (0.5) | 207 (0.7) | 18 (0.8) | 223 (0.7) | 9 (0.6) | 196 (0.6) | 11 (0.8) |
| Anticoagulant use**= 1 (%) | 1620 (5.1) | 121 (8.2) | 1679 (5.2) | 116 (7.9) | 1705 (5.2) | 77 (8.7) | 1680 (5.1) | 74 (8.3) | 1602 (5.1) | 158 (7.1) | 1614 (5.2) | 162 (7.3) | 1611 (5.0) | 104 (7.4) | 1711 (5.3) | 110 (7.8) |
| Antidepressants TCA**= 1 (%) | 2578 (8.0) | 144 (9.8) | 2507 (7.8) | 123 (8.4) | 2646 (8.1) | 92 (10.3) | 2540 (7.8) | 74 (8.3) | 2529 (8.1) | 191 (8.6) | 2430 (7.8) | 202 (9.1) | 2578 (8.0) | 122 (8.6) | 2519 (7.8) | 133 (9.4) |
| Antidepressants SSRI**= 1 (%) | 3442 (10.7) | 170 (11.6) | 3472 (10.8) | 209 (14.2) | 3519 (10.8) | 99 (11.1) | 3565 (10.9) | 110 (12.4) | 3394 (10.8) | 274 (12.4) | 3350 (10.7) | 275 (12.4) | 3547 (11.0) | 162 (11.5) | 3436 (10.7) | 148 (10.5) |
| Hypercholesterolemia**= 1 (%) | 297 (0.9) | 15 (1.0) | 338 (1.1) | 17 (1.2) | 346 (1.1) | 15 (1.7) | 299 (0.9) | 7 (0.8) | 319 (1.0) | 19 (0.9) | 303 (1.0) | 26 (1.2) | 327 (1.0) | 11 (0.8) | 307 (1.0) | 22 (1.6) |
| Statin use**= 1 (%) | 6667 (20.8) | 394 (26.8) | 6630 (20.7) | 393 (26.7) | 6788 (20.8) | 246 (27.7) | 6796 (20.8) | 254 (28.6) | 6490 (20.7) | 546 (24.7) | 6465 (20.6) | 583 (26.4) | 6642 (20.7) | 362 (25.6) | 6690 (20.8) | 390 (27.6) |
| Osteoporosis history* = 1 | 2652 (8.3) | 124 (8.4) | 2636 (8.2) | 109 (7.4) | 2729 (8.4) | 83 (9.3) | 2640 (8.1) | 69 (7.8) | 2617 (8.4) | 160 (7.2) | 2548 (8.1) | 196 (8.9) | 2674 (8.3) | 113 (8.0) | 2607 (8.1) | 127 (9.0) |
| Family history of cardiovascular disease = 1 (%) | 1916 (6.0) | 89 (6.1) | 1942 (6.1) | 89 (6.1) | 1928 (5.9) | 64 (7.2) | 1991 (6.1) | 53 (6.0) | 1899 (6.1) | 132 (6.0) | 1883 (6.0) | 122 (5.5) | 1912 (6.0) | 90 (6.4) | 1950 (6.1) | 84 (5.9) |
| Family history of cardiovascular disease before age 60= 1 (%) | 27 (0.1) | 0 (0.0) | 20 (0.1) | 0 (0.0) | 26 (0.1) | 0 (0.0) | 21 (0.1) | 0 (0.0) | 27 (0.1) | 0 (0.0) | 20 (0.1) | 0 (0.0) | 26 (0.1) | 0 (0.0) | 21 (0.1) | 0 (0.0) |
| Heart failure*= 1 (%) | 1573 (4.9) | 135 (9.2) | 1565 (4.9) | 132 (9.0) | 1701 (5.2) | 68 (7.6) | 1559 (4.8) | 77 (8.7) | 1536 (4.9) | 182 (8.2) | 1504 (4.8) | 183 (8.3) | 1558 (4.9) | 104 (7.4) | 1631 (5.1) | 112 (7.9) |
| Migraine*= 1 (%) | 2991 (9.3) | 153 (10.4) | 3012 (9.4) | 138 (9.4) | 3022 (9.3) | 93 (10.5) | 3075 (9.4) | 104 (11.7) | 2933 (9.4) | 220 (10.0) | 2926 (9.3) | 215 (9.7) | 3042 (9.5) | 141 (10.0) | 2949 (9.2) | 162 (11.5) |
| Severe mental illness*= 1 (%) | 4202 (13.1) | 181 (12.3) | 4239 (13.2) | 214 (14.6) | 4360 (13.4) | 129 (14.5) | 4221 (12.9) | 126 (14.2) | 4138 (13.2) | 321 (14.5) | 4090 (13.1) | 287 (13.0) | 4294 (13.4) | 202 (14.3) | 4133 (12.9) | 207 (14.7) |
| Vascular Disease*= 1 (%) | 504 (1.6) | 41 (2.8) | 483 (1.5) | 50 (3.4) | 520 (1.6) | 30 (3.4) | 496 (1.5) | 32 (3.6) | 483 (1.5) | 63 (2.8) | 470 (1.5) | 62 (2.8) | 494 (1.5) | 49 (3.5) | 497 (1.5) | 38 (2.7) |
| Atrial fibrillation*= 1 (%) | 2164 (6.7) | 189 (12.8) | 2261 (7.1) | 170 (11.6) | 2291 (7.0) | 113 (12.7) | 2270 (7.0) | 110 (12.4) | 2181 (7.0) | 219 (9.9) | 2106 (6.7) | 278 (12.6) | 2141 (6.7) | 154 (10.9) | 2323 (7.2) | 166 (11.8) |
| On anti-hypertensive drug= 1 (%) | 18018 (56.2) | 1030 (70.0) | 17937 (55.9) | 1051 (71.5) | 18454 (56.5) | 623 (70.1) | 18332 (56.2) | 627 (70.5) | 17442 (55.7) | 1501 (67.9) | 17568 (56.1) | 1525 (69.0) | 18037 (56.2) | 954 (67.6) | 18073 (56.3) | 972 (68.8) |
| Antipsychotic use**= 1 (%) | 397 (1.2) | 27 (1.8) | 415 (1.3) | 28 (1.9) | 422 (1.3) | 12 (1.3) | 421 (1.3) | 12 (1.3) | 381 (1.2) | 42 (1.9) | 412 (1.3) | 32 (1.4) | 427 (1.3) | 17 (1.2) | 406 (1.3) | 17 (1.2) |
| Steroid use**= 1 (%) | 2454 (7.7) | 118 (8.0) | 2494 (7.8) | 115 (7.8) | 2496 (7.6) | 69 (7.8) | 2530 (7.8) | 86 (9.7) | 2420 (7.7) | 162 (7.3) | 2427 (7.7) | 172 (7.8) | 2467 (7.7) | 107 (7.6) | 2486 (7.7) | 121 (8.6) |
| Erectile dysfunction**= 1 (%) | 501 (1.6) | 24 (1.6) | 519 (1.6) | 21 (1.4) | 510 (1.6) | 11 (1.2) | 528 (1.6) | 16 (1.8) | 493 (1.6) | 38 (1.7) | 501 (1.6) | 33 (1.5) | 503 (1.6) | 25 (1.8) | 510 (1.6) | 27 (1.9) |
| Age>75 | 23082 (72.0) | 1314 (89.3) | 23006 (71.8) | 1324 (90.1) | 23607 (72.3) | 782 (88.0) | 23564 (72.2) | 773 (87.0) | 22452 (71.7) | 1975 (89.3) | 22348 (71.3) | 1951 (88.3) | 23039 (71.7) | 1242 (88.0) | 23229 (72.3) | 1216 (86.1) |
| Age Group (%) |  |  |  |  |  |  |  |  |  |  |  |  |  |  |  |  |
| 50-59 | 2364 (7.4) | 13 (0.9) | 2379 (7.4) | 8 (0.5) | 2368 (7.3) | 10 (1.1) | 2377 (7.3) | 9 (1.0) | 2326 (7.4) | 21 (0.9) | 2395 (7.6) | 22 (1.0) | 2383 (7.4) | 12 (0.8) | 2346 (7.3) | 23 (1.6) |
| 60-69 | 3753 (11.7) | 63 (4.3) | 3765 (11.7) | 65 (4.4) | 3781 (11.6) | 46 (5.2) | 3770 (11.5) | 49 (5.5) | 3752 (12.0) | 105 (4.7) | 3689 (11.8) | 100 (4.5) | 3814 (11.9) | 69 (4.9) | 3679 (11.5) | 84 (5.9) |
| 70-79 | 7553 (23.6) | 263 (17.9) | 7501 (23.4) | 256 (17.4) | 7605 (23.3) | 169 (19.0) | 7597 (23.3) | 202 (22.7) | 7361 (23.5) | 433 (19.6) | 7386 (23.6) | 393 (17.8) | 7431 (23.1) | 302 (21.4) | 7542 (23.5) | 298 (21.1) |
| 80-89 | 13010 (40.6) | 736 (50.0) | 13064 (40.7) | 736 (50.1) | 13293 (40.7) | 455 (51.2) | 13354 (40.9) | 444 (49.9) | 12648 (40.4) | 1143 (51.7) | 12633 (40.3) | 1122 (50.8) | 12988 (40.4) | 730 (51.7) | 13088 (40.7) | 740 (52.4) |
| >89 | 5382 (16.8) | 396 (26.9) | 5353 (16.7) | 405 (27.6) | 5597 (17.1) | 209 (23.5) | 5545 (17.0) | 185 (20.8) | 5235 (16.7) | 509 (23.0) | 5219 (16.7) | 573 (25.9) | 5505 (17.1) | 299 (21.2) | 5465 (17.0) | 267 (18.9) |
| Charlson score (%) |  |  |  |  |  |  |  |  |  |  |  |  |  |  |  |  |
| 0 | 19232 (60.0) | 734 (49.9) | 19345 (60.3) | 713 (48.5) | 19465 (59.6) | 449 (50.5) | 19678 (60.3) | 432 (48.6) | 18912 (60.4) | 1133 (51.2) | 18840 (60.1) | 1139 (51.5) | 19414 (60.4) | 753 (53.3) | 19145 (59.6) | 712 (50.4) |
| 1 | 5337 (16.6) | 266 (18.1) | 5324 (16.6) | 282 (19.2) | 5493 (16.8) | 162 (18.2) | 5395 (16.5) | 159 (17.9) | 5145 (16.4) | 412 (18.6) | 5255 (16.8) | 397 (18.0) | 5275 (16.4) | 250 (17.7) | 5440 (16.9) | 244 (17.3) |
| 2 | 3530 (11.0) | 192 (13.1) | 3453 (10.8) | 213 (14.5) | 3607 (11.0) | 127 (14.3) | 3537 (10.8) | 117 (13.2) | 3421 (10.9) | 278 (12.6) | 3388 (10.8) | 301 (13.6) | 3445 (10.7) | 174 (12.3) | 3577 (11.1) | 192 (13.6) |
| ≥3 | 3963 (12.4) | 279 (19.0) | 3940 (12.3) | 262 (17.8) | 4079 (12.5) | 151 (17.0) | 4033 (12.4) | 181 (20.4) | 3844 (12.3) | 388 (17.5) | 3839 (12.3) | 373 (16.9) | 3987 (12.4) | 235 (16.6) | 3958 (12.3) | 264 (18.7) |
| Cardiovascular disease (%) |  |  |  |  |  |  |  |  |  |  |  |  |  |  |  |  |
| No | 27974 (87.2) | 1159 (78.8) | 28066 (87.5) | 1182 (80.4) | 28521 (87.4) | 702 (79.0) | 28474 (87.2) | 684 (76.9) | 27426 (87.6) | 1758 (79.5) | 27422 (87.5) | 1775 (80.3) | 28078 (87.4) | 1120 (79.3) | 28095 (87.5) | 1088 (77.1) |
| Ever >1 year before index date | 3321 (10.4) | 233 (15.8) | 3230 (10.1) | 212 (14.4) | 3338 (10.2) | 135 (15.2) | 3372 (10.3) | 151 (17.0) | 3138 (10.0) | 343 (15.5) | 3190 (10.2) | 325 (14.7) | 3266 (10.2) | 211 (14.9) | 3272 (10.2) | 247 (17.5) |
| 1 year before index | 321 (1.0) | 34 (2.3) | 360 (1.1) | 28 (1.9) | 351 (1.1) | 17 (1.9) | 351 (1.1) | 24 (2.7) | 334 (1.1) | 43 (1.9) | 324 (1.0) | 42 (1.9) | 354 (1.1) | 28 (2.0) | 333 (1.0) | 28 (2.0) |
| 6 months before index | 370 (1.2) | 30 (2.0) | 333 (1.0) | 34 (2.3) | 357 (1.1) | 24 (2.7) | 365 (1.1) | 21 (2.4) | 349 (1.1) | 46 (2.1) | 322 (1.0) | 50 (2.3) | 341 (1.1) | 35 (2.5) | 352 (1.1) | 39 (2.8) |
| 1 month before index | 76 (0.2) | 15 (1.0) | 73 (0.2) | 14 (1.0) | 77 (0.2) | 11 (1.2) | 81 (0.2) | 9 (1.0) | 75 (0.2) | 21 (0.9) | 64 (0.2) | 18 (0.8) | 82 (0.3) | 18 (1.3) | 68 (0.2) | 10 (0.7) |
| MI or Stroke (%) |  |  |  |  |  |  |  |  |  |  |  |  |  |  |  |  |
| No | 28874 (90.1) | 1129 (76.8) | 28884 (90.1) | 1159 (78.8) | 29311 (89.8) | 713 (80.2) | 29323 (89.8) | 699 (78.6) | 28314 (90.4) | 1745 (78.9) | 28260 (90.2) | 1727 (78.1) | 28850 (89.8) | 1122 (79.5) | 28934 (90.1) | 1140 (80.7) |
| Ever >1 year before index date | 2320 (7.2) | 238 (16.2) | 2313 (7.2) | 212 (14.4) | 2406 (7.4) | 119 (13.4) | 2431 (7.4) | 127 (14.3) | 2193 (7.0) | 314 (14.2) | 2245 (7.2) | 331 (15.0) | 2365 (7.4) | 190 (13.5) | 2335 (7.3) | 193 (13.7) |
| 1 year before index | 868 (2.7) | 104 (7.1) | 865 (2.7) | 99 (6.7) | 927 (2.8) | 57 (6.4) | 889 (2.7) | 63 (7.1) | 815 (2.6) | 152 (6.9) | 817 (2.6) | 152 (6.9) | 906 (2.8) | 100 (7.1) | 851 (2.6) | 79 (5.6) |
| Established CVD *= Ever (%) | 5305 (16.5) | 474 (32.2) | 5242 (16.3) | 485 (33.0) | 5500 (16.8) | 263 (29.6) | 5457 (16.7) | 286 (32.2) | 5062 (16.2) | 669 (30.3) | 5073 (16.2) | 702 (31.8) | 5322 (16.6) | 412 (29.2) | 5357 (16.7) | 415 (29.4) |
| Any fracture history (%) |  |  |  |  |  |  |  |  |  |  |  |  |  |  |  |  |
| No | 32062 (100.0) | 1471 (100.0) | 32062 (100.0) | 1470 (100.0) | 32644 (100.0) | 889 (100.0) | 32643 (100.0) | 889 (100.0) | 31322 (100.0) | 2211 (100.0) | 31322 (100.0) | 2210 (100.0) | 32121 (100.0) | 1412 (100.0) | 32120 (100.0) | 1412 (100.0) |
| Ever >1 year before index date | 0 | 0 | 0 | 0 | 0 | 0 | 0 | 0 | 0 | 0 | 0 | 0 | 0 | 0 | 0 | 0 |
| 1 year before index | 0 | 0 | 0 | 0 | 0 | 0 | 0 | 0 | 0 | 0 | 0 | 0 | 0 | 0 | 0 | 0 |
| Hip fracture history (%) |  |  |  |  |  |  |  |  |  |  |  |  |  |  |  |  |
| No | 32062 (100.0) | 1471 (100.0) | 32062 (100.0) | 1470 (100.0) | 32644 (100.0) | 889 (100.0) | 32643 (100.0) | 889 (100.0) | 31322 (100.0) | 2211 (100.0) | 31322 (100.0) | 2210 (100.0) | 32121 (100.0) | 1412 (100.0) | 32120 (100.0) | 1412 (100.0) |
| Ever >1 year before index date | 0 | 0 | 0 | 0 | 0 | 0 | 0 | 0 | 0 | 0 | 0 | 0 | 0 | 0 | 0 | 0 |
| 1 year before index | 0 | 0 | 0 | 0 | 0 | 0 | 0 | 0 | 0 | 0 | 0 | 0 | 0 | 0 | 0 | 0 |
| Shoulder fracture history (%) |  |  |  |  |  |  |  |  |  |  |  |  |  |  |  |  |
| No | 32062 (100.0) | 1471 (100.0) | 32062 (100.0) | 1470 (100.0) | 32644 (100.0) | 889 (100.0) | 32643 (100.0) | 889 (100.0) | 31322 (100.0) | 2211 (100.0) | 31322 (100.0) | 2210 (100.0) | 32121 (100.0) | 1412 (100.0) | 32120 (100.0) | 1412 (100.0) |
| Ever | 0 | 0 | 0 | 0 | 0 | 0 | 0 | 0 | 0 | 0 | 0 | 0 | 0 | 0 | 0 | 0 |
| 1 year before index | 0 | 0 | 0 | 0 | 0 | 0 | 0 | 0 | 0 | 0 | 0 | 0 | 0 | 0 | 0 | 0 |
| Spine fracture history (%) |  |  |  |  |  |  |  |  |  |  |  |  |  |  |  |  |
| No | 32062 (100.0) | 1471 (100.0) | 32062 (100.0) | 1470 (100.0) | 32644 (100.0) | 889 (100.0) | 32643 (100.0) | 889 (100.0) | 31322 (100.0) | 2211 (100.0) | 31322 (100.0) | 2210 (100.0) | 32121 (100.0) | 1412 (100.0) | 32120 (100.0) | 1412 (100.0) |
| Ever >1 year before index date | 0 | 0 | 0 | 0 | 0 | 0 | 0 | 0 | 0 | 0 | 0 | 0 | 0 | 0 | 0 | 0 |
| 1 year before index | 0 | 0 | 0 | 0 | 0 | 0 | 0 | 0 | 0 | 0 | 0 | 0 | 0 | 0 | 0 | 0 |
| Wrist fracture history (%) |  |  |  |  |  |  |  |  |  |  |  |  |  |  |  |  |
| No | 32062 (100.0) | 1471 (100.0) | 32062 (100.0) | 1470 (100.0) | 32644 (100.0) | 889 (100.0) | 32643 (100.0) | 889 (100.0) | 31322 (100.0) | 2211 (100.0) | 31322 (100.0) | 2210 (100.0) | 32121 (100.0) | 1412 (100.0) | 32120 (100.0) | 1412 (100.0) |
| Ever >1 year before index date | 0 | 0 | 0 | 0 | 0 | 0 | 0 | 0 | 0 | 0 | 0 | 0 | 0 | 0 | 0 | 0 |
| 1 year before index | 0 | 0 | 0 | 0 | 0 | 0 | 0 | 0 | 0 | 0 | 0 | 0 | 0 | 0 | 0 | 0 |
| BMI** (%) |  |  |  |  |  |  |  |  |  |  |  |  |  |  |  |  |
| <18.5 | 4366 (13.6) | 232 (15.8) | 4465 (13.9) | 202 (13.7) | 4499 (13.8) | 124 (13.9) | 4518 (13.8) | 124 (13.9) | 4273 (13.6) | 350 (15.8) | 4310 (13.8) | 332 (15.0) | 4443 (13.8) | 202 (14.3) | 4415 (13.7) | 205 (14.5) |
| 18.6 - 24.9 | 16100 (50.2) | 753 (51.2) | 15920 (49.7) | 781 (53.1) | 16274 (49.9) | 447 (50.3) | 16381 (50.2) | 452 (50.8) | 15547 (49.6) | 1143 (51.7) | 15692 (50.1) | 1172 (53.0) | 16120 (50.2) | 702 (49.7) | 16014 (49.9) | 718 (50.8) |
| 25 - 29.9 | 7942 (24.8) | 348 (23.7) | 8084 (25.2) | 346 (23.5) | 8189 (25.1) | 225 (25.3) | 8091 (24.8) | 215 (24.2) | 7937 (25.3) | 517 (23.4) | 7762 (24.8) | 504 (22.8) | 7931 (24.7) | 347 (24.6) | 8094 (25.2) | 348 (24.6) |
| 30 - 39.9 | 3400 (10.6) | 128 (8.7) | 3324 (10.4) | 131 (8.9) | 3429 (10.5) | 86 (9.7) | 3378 (10.3) | 90 (10.1) | 3291 (10.5) | 189 (8.5) | 3313 (10.6) | 190 (8.6) | 3364 (10.5) | 150 (10.6) | 3336 (10.4) | 133 (9.4) |
| >=40 | 254 (0.8) | 10 (0.7) | 269 (0.8) | 10 (0.7) | 253 (0.8) | 7 (0.8) | 275 (0.8) | 8 (0.9) | 274 (0.9) | 12 (0.5) | 245 (0.8) | 12 (0.5) | 263 (0.8) | 11 (0.8) | 261 (0.8) | 8 (0.6) |
| No. of GP visits** (%) |  |  |  |  |  |  |  |  |  |  |  |  |  |  |  |  |
| 0 | 7195 (22.4) | 247 (16.8) | 7339 (22.9) | 236 (16.1) | 7306 (22.4) | 166 (18.7) | 7393 (22.6) | 152 (17.1) | 7121 (22.7) | 410 (18.5) | 7082 (22.6) | 404 (18.3) | 7252 (22.6) | 293 (20.8) | 7199 (22.4) | 273 (19.3) |
| 1-5 | 7355 (22.9) | 306 (20.8) | 7535 (23.5) | 332 (22.6) | 7654 (23.4) | 175 (19.7) | 7499 (23.0) | 200 (22.5) | 7201 (23.0) | 470 (21.3) | 7378 (23.6) | 479 (21.7) | 7464 (23.2) | 285 (20.2) | 7476 (23.3) | 303 (21.5) |
| 6-10 | 6574 (20.5) | 318 (21.6) | 6498 (20.3) | 287 (19.5) | 6756 (20.7) | 197 (22.2) | 6558 (20.1) | 166 (18.7) | 6451 (20.6) | 461 (20.9) | 6319 (20.2) | 446 (20.2) | 6523 (20.3) | 288 (20.4) | 6585 (20.5) | 281 (19.9) |
| 11-15 | 4375 (13.6) | 210 (14.3) | 4331 (13.5) | 224 (15.2) | 4410 (13.5) | 125 (14.1) | 4480 (13.7) | 125 (14.1) | 4282 (13.7) | 339 (15.3) | 4200 (13.4) | 319 (14.4) | 4342 (13.5) | 211 (14.9) | 4394 (13.7) | 193 (13.7) |
| >=16 | 6563 (20.5) | 390 (26.5) | 6359 (19.8) | 391 (26.6) | 6518 (20.0) | 226 (25.4) | 6713 (20.6) | 246 (27.7) | 6267 (20.0) | 531 (24.0) | 6343 (20.3) | 562 (25.4) | 6540 (20.4) | 335 (23.7) | 6466 (20.1) | 362 (25.6) |
| No. of GP emergency visits** (%) |  |  |  |  |  |  |  |  |  |  |  |  |  |  |  |  |
| 0 | 25609 (79.9) | 1031 (70.1) | 25552 (79.7) | 1029 (70.0) | 25938 (79.5) | 675 (75.9) | 25964 (79.5) | 644 (72.4) | 25107 (80.2) | 1569 (71.0) | 24953 (79.7) | 1592 (72.0) | 25612 (79.7) | 1060 (75.1) | 25483 (79.3) | 1066 (75.5) |
| 1 | 2983 (9.3) | 190 (12.9) | 3038 (9.5) | 181 (12.3) | 3124 (9.6) | 103 (11.6) | 3052 (9.3) | 113 (12.7) | 2888 (9.2) | 274 (12.4) | 2964 (9.5) | 266 (12.0) | 2966 (9.2) | 160 (11.3) | 3102 (9.7) | 164 (11.6) |
| 2 | 1287 (4.0) | 94 (6.4) | 1367 (4.3) | 96 (6.5) | 1354 (4.1) | 45 (5.1) | 1385 (4.2) | 60 (6.7) | 1296 (4.1) | 133 (6.0) | 1276 (4.1) | 139 (6.3) | 1285 (4.0) | 76 (5.4) | 1406 (4.4) | 77 (5.5) |
| 3-5 | 1496 (4.7) | 101 (6.9) | 1449 (4.5) | 117 (8.0) | 1541 (4.7) | 46 (5.2) | 1525 (4.7) | 51 (5.7) | 1389 (4.4) | 161 (7.3) | 1472 (4.7) | 141 (6.4) | 1550 (4.8) | 82 (5.8) | 1458 (4.5) | 73 (5.2) |
| >=6 | 687 (2.1) | 55 (3.7) | 656 (2.0) | 47 (3.2) | 687 (2.1) | 20 (2.2) | 717 (2.2) | 21 (2.4) | 642 (2.0) | 74 (3.3) | 657 (2.1) | 72 (3.3) | 708 (2.2) | 34 (2.4) | 671 (2.1) | 32 (2.3) |
| eGFR** (%) |  |  |  |  |  |  |  |  |  |  |  |  |  |  |  |  |
| <=29 | 890 (2.8) | 83 (5.6) | 914 (2.9) | 87 (5.9) | 960 (2.9) | 48 (5.4) | 925 (2.8) | 41 (4.6) | 888 (2.8) | 105 (4.7) | 874 (2.8) | 107 (4.8) | 920 (2.9) | 56 (4.0) | 932 (2.9) | 66 (4.7) |
| 30 - 44 | 3247 (10.1) | 278 (18.9) | 3306 (10.3) | 268 (18.2) | 3370 (10.3) | 165 (18.6) | 3432 (10.5) | 132 (14.8) | 3209 (10.2) | 360 (16.3) | 3166 (10.1) | 364 (16.5) | 3330 (10.4) | 218 (15.4) | 3331 (10.4) | 220 (15.6) |
| 45 - 59 | 7289 (22.7) | 418 (28.4) | 7202 (22.5) | 426 (29.0) | 7439 (22.8) | 234 (26.3) | 7416 (22.7) | 246 (27.7) | 7048 (22.5) | 581 (26.3) | 7100 (22.7) | 606 (27.4) | 7287 (22.7) | 372 (26.3) | 7336 (22.8) | 340 (24.1) |
| 60 - 89 | 19223 (60.0) | 651 (44.3) | 19244 (60.0) | 656 (44.6) | 19418 (59.5) | 413 (46.5) | 19496 (59.7) | 447 (50.3) | 18829 (60.1) | 1098 (49.7) | 18778 (60.0) | 1069 (48.4) | 19201 (59.8) | 726 (51.4) | 19109 (59.5) | 738 (52.3) |
| >=90 | 1413 (4.4) | 41 (2.8) | 1396 (4.4) | 33 (2.2) | 1457 (4.5) | 29 (3.3) | 1374 (4.2) | 23 (2.6) | 1348 (4.3) | 67 (3.0) | 1404 (4.5) | 64 (2.9) | 1383 (4.3) | 40 (2.8) | 1412 (4.4) | 48 (3.4) |
| SBP** (%) |  |  |  |  |  |  |  |  |  |  |  |  |  |  |  |  |
| <120 | 4436 (13.8) | 196 (13.3) | 4366 (13.6) | 186 (12.7) | 4446 (13.6) | 92 (10.3) | 4539 (13.9) | 107 (12.0) | 4254 (13.6) | 268 (12.1) | 4389 (14.0) | 273 (12.4) | 4364 (13.6) | 166 (11.8) | 4515 (14.1) | 139 (9.8) |
| 120 - 139 | 12354 (38.5) | 511 (34.7) | 12478 (38.9) | 529 (36.0) | 12702 (38.9) | 324 (36.4) | 12550 (38.4) | 296 (33.3) | 12112 (38.7) | 761 (34.4) | 12211 (39.0) | 788 (35.7) | 12534 (39.0) | 473 (33.5) | 12352 (38.5) | 513 (36.3) |
| 140 - 159 | 11101 (34.6) | 529 (36.0) | 11082 (34.6) | 501 (34.1) | 11269 (34.5) | 320 (36.0) | 11310 (34.6) | 314 (35.3) | 10892 (34.8) | 781 (35.3) | 10774 (34.4) | 766 (34.7) | 11107 (34.6) | 484 (34.3) | 11119 (34.6) | 503 (35.6) |
| >=160 | 4171 (13.0) | 235 (16.0) | 4136 (12.9) | 254 (17.3) | 4227 (12.9) | 153 (17.2) | 4244 (13.0) | 172 (19.3) | 4064 (13.0) | 401 (18.1) | 3948 (12.6) | 383 (17.3) | 4116 (12.8) | 289 (20.5) | 4134 (12.9) | 257 (18.2) |
| DBP** (%) |  |  |  |  |  |  |  |  |  |  |  |  |  |  |  |  |
| <80 | 17282 (53.9) | 797 (54.2) | 17340 (54.1) | 828 (56.3) | 17661 (54.1) | 481 (54.1) | 17640 (54.0) | 465 (52.3) | 16856 (53.8) | 1202 (54.4) | 16949 (54.1) | 1240 (56.1) | 17317 (53.9) | 755 (53.5) | 17408 (54.2) | 767 (54.3) |
| 80 - 89 | 11219 (35.0) | 509 (34.6) | 11275 (35.2) | 465 (31.6) | 11508 (35.3) | 301 (33.9) | 11359 (34.8) | 300 (33.7) | 10979 (35.1) | 743 (33.6) | 11051 (35.3) | 695 (31.4) | 11274 (35.1) | 469 (33.2) | 11266 (35.1) | 459 (32.5) |
| 90 - 99 | 2766 (8.6) | 129 (8.8) | 2710 (8.5) | 127 (8.6) | 2704 (8.3) | 78 (8.8) | 2857 (8.8) | 93 (10.5) | 2700 (8.6) | 206 (9.3) | 2619 (8.4) | 207 (9.4) | 2734 (8.5) | 139 (9.8) | 2715 (8.5) | 144 (10.2) |
| >=100 | 795 (2.5) | 36 (2.4) | 737 (2.3) | 50 (3.4) | 771 (2.4) | 29 (3.3) | 787 (2.4) | 31 (3.5) | 787 (2.5) | 60 (2.7) | 703 (2.2) | 68 (3.1) | 796 (2.5) | 49 (3.5) | 731 (2.3) | 42 (3.0) |
| No. of concomitant medicines** (%) |  |  |  |  |  |  |  |  |  |  |  |  |  |  |  |  |
| 0 | 7700 (24.0) | 243 (16.5) | 7891 (24.6) | 238 (16.2) | 7818 (23.9) | 161 (18.1) | 7937 (24.3) | 156 (17.5) | 7603 (24.3) | 422 (19.1) | 7647 (24.4) | 400 (18.1) | 7804 (24.3) | 298 (21.1) | 7695 (24.0) | 275 (19.5) |
| 1 - 3 | 5023 (15.7) | 161 (10.9) | 4888 (15.2) | 150 (10.2) | 5090 (15.6) | 93 (10.5) | 4952 (15.2) | 87 (9.8) | 4893 (15.6) | 252 (11.4) | 4837 (15.4) | 240 (10.9) | 4945 (15.4) | 140 (9.9) | 4971 (15.5) | 166 (11.8) |
| 4 - 6 | 6052 (18.9) | 279 (19.0) | 5959 (18.6) | 282 (19.2) | 6158 (18.9) | 153 (17.2) | 6093 (18.7) | 168 (18.9) | 5840 (18.6) | 425 (19.2) | 5902 (18.8) | 405 (18.3) | 6030 (18.8) | 278 (19.7) | 6034 (18.8) | 230 (16.3) |
| 7 - 9 | 5110 (15.9) | 251 (17.1) | 5266 (16.4) | 303 (20.6) | 5314 (16.3) | 173 (19.5) | 5292 (16.2) | 151 (17.0) | 5080 (16.2) | 401 (18.1) | 5028 (16.1) | 421 (19.0) | 5196 (16.2) | 246 (17.4) | 5232 (16.3) | 256 (18.1) |
| 10 - 12 | 3638 (11.3) | 230 (15.6) | 3600 (11.2) | 207 (14.1) | 3692 (11.3) | 128 (14.4) | 3731 (11.4) | 124 (13.9) | 3468 (11.1) | 305 (13.8) | 3577 (11.4) | 325 (14.7) | 3625 (11.3) | 186 (13.2) | 3663 (11.4) | 201 (14.2) |
| >=13 | 4539 (14.2) | 307 (20.9) | 4458 (13.9) | 290 (19.7) | 4572 (14.0) | 181 (20.4) | 4638 (14.2) | 203 (22.8) | 4438 (14.2) | 406 (18.4) | 4331 (13.8) | 419 (19.0) | 4521 (14.1) | 264 (18.7) | 4525 (14.1) | 284 (20.1) |
| Cholesterol measurement** (HDL/LDL) (%) |  |  |  |  |  |  |  |  |  |  |  |  |  |  |  |  |
| <=3.5 | 20268 (63.2) | 1113 (75.7) | 20396 (63.6) | 1115 (75.9) | 20839 (63.8) | 732 (82.3) | 20600 (63.1) | 721 (81.1) | 19680 (62.8) | 1692 (76.5) | 19821 (63.3) | 1699 (76.9) | 20237 (63.0) | 1142 (80.9) | 20359 (63.4) | 1154 (81.7) |
| 3.6 - 5 | 9059 (28.3) | 278 (18.9) | 9010 (28.1) | 283 (19.3) | 9108 (27.9) | 127 (14.3) | 9265 (28.4) | 130 (14.6) | 8918 (28.5) | 424 (19.2) | 8878 (28.3) | 410 (18.6) | 9207 (28.7) | 216 (15.3) | 8993 (28.0) | 214 (15.2) |
| >5 | 2735 (8.5) | 80 (5.4) | 2656 (8.3) | 72 (4.9) | 2697 (8.3) | 30 (3.4) | 2778 (8.5) | 38 (4.3) | 2724 (8.7) | 95 (4.3) | 2623 (8.4) | 101 (4.6) | 2677 (8.3) | 54 (3.8) | 2768 (8.6) | 44 (3.1) |
| No. of previous fractures* (%) |  |  |  |  |  |  |  |  |  |  |  |  |  |  |  |  |
| 0 | NA | NA | NA | NA | NA | NA | NA | NA | NA | NA | NA | NA | NA | NA | NA | NA |
| 1 | NA | NA | NA | NA | NA | NA | NA | NA | NA | NA | NA | NA | NA | NA | NA | NA |
| >=2 | NA | NA | NA | NA | NA | NA | NA | NA | NA | NA | NA | NA | NA | NA | NA | NA |
| **Abbreviations**: OST, patients with incident diagnosis of osteoporosis; IFX, patients with incident fragility fracture; OBP, incident users of oral bisphosphonates; MACE, composite outcome for the occurrence of either myocardial infarction, stroke or cardiovascular disease death; MI, myocardial infarction; * ever; ** in the year prior to start; SES, socio-economic status; BMI, body mass index; eGFR, estimated Glomerular Filtration Rate; SBP, cholesterol, systolic blood pressure; DBP, diastolic blood pressure. | | | | | | | | | | | | | | | | |

# Table S2c Dataset split into train and test sets, stratified by outcome (OBP cohort)

| **OBP cohort** | **One year** | | | | | | | | **Two years** | | | | | | | |
| --- | --- | --- | --- | --- | --- | --- | --- | --- | --- | --- | --- | --- | --- | --- | --- | --- |
|  | **MACE** | | | | **Stroke/MI** | | | | **MACE** | | | | **Stroke/MI** | | | |
|  | **Development set (n=72980)** | | **Internal validation set (n=72979)** | | **Development set (n=72980)** | | **Internal validation set (n=72979)** | | **Development set (n=72980)** | | **Internal validation set (n=72979)** | | **Development set (n=72980)** | | **Internal validation set (n=72979)** | |
|  | **Outcome=NO** | **Outcome=YES** | **Outcome=NO** | **Outcome=YES** | **Outcome=NO** | **Outcome=YES** | **Outcome=NO** | **Outcome=YES** | **Outcome=NO** | **Outcome=YES** | **Outcome=NO** | **Outcome=YES** | **Outcome=NO** | **Outcome=YES** | **Outcome=NO** | **Outcome=YES** |
| n | 71168 | 1812 | 71168 | 1811 | 71547 | 1433 | 71546 | 1433 | 69745 | 3235 | 69744 | 3235 | 70427 | 2553 | 70427 | 2552 |
| Sex = Male (%) | 14170 (19.9) | 493 (27.2) | 14413 (20.3) | 471 (26.0) | 14336 (20.0) | 372 (26.0) | 14438 (20.2) | 401 (28.0) | 13869 (19.9) | 875 (27.0) | 13984 (20.1) | 819 (25.3) | 14206 (20.2) | 676 (26.5) | 13996 (19.9) | 669 (26.2) |
| SES (%) |  |  |  |  |  |  |  |  |  |  |  |  |  |  |  |  |
| 1 | 17629 (24.8) | 406 (22.4) | 17444 (24.5) | 358 (19.8) | 17665 (24.7) | 309 (21.6) | 17583 (24.6) | 280 (19.5) | 17238 (24.7) | 682 (21.1) | 17176 (24.6) | 741 (22.9) | 17254 (24.5) | 555 (21.7) | 17462 (24.8) | 566 (22.2) |
| 2 | 17462 (24.5) | 435 (24.0) | 17405 (24.5) | 436 (24.1) | 17541 (24.5) | 338 (23.6) | 17518 (24.5) | 341 (23.8) | 17125 (24.6) | 773 (23.9) | 17108 (24.5) | 732 (22.6) | 17302 (24.6) | 599 (23.5) | 17257 (24.5) | 580 (22.7) |
| 3 | 15052 (21.1) | 396 (21.9) | 15030 (21.1) | 399 (22.0) | 15098 (21.1) | 314 (21.9) | 15154 (21.2) | 311 (21.7) | 14656 (21.0) | 718 (22.2) | 14766 (21.2) | 737 (22.8) | 14845 (21.1) | 551 (21.6) | 14910 (21.2) | 571 (22.4) |
| 4 | 12771 (17.9) | 334 (18.4) | 13096 (18.4) | 365 (20.2) | 12935 (18.1) | 279 (19.5) | 13059 (18.3) | 293 (20.4) | 12702 (18.2) | 638 (19.7) | 12636 (18.1) | 590 (18.2) | 12870 (18.3) | 486 (19.0) | 12708 (18.0) | 502 (19.7) |
| 5 | 8194 (11.5) | 236 (13.0) | 8146 (11.4) | 252 (13.9) | 8251 (11.5) | 191 (13.3) | 8178 (11.4) | 208 (14.5) | 7966 (11.4) | 419 (13.0) | 8012 (11.5) | 431 (13.3) | 8097 (11.5) | 360 (14.1) | 8038 (11.4) | 333 (13.0) |
| Smoking** (%) |  |  |  |  |  |  |  |  |  |  |  |  |  |  |  |  |
| Ex | 24339 (34.2) | 626 (34.5) | 24602 (34.6) | 595 (32.9) | 24541 (34.3) | 486 (33.9) | 24624 (34.4) | 511 (35.7) | 24015 (34.4) | 1132 (35.0) | 23855 (34.2) | 1160 (35.9) | 24344 (34.6) | 922 (36.1) | 23984 (34.1) | 912 (35.7) |
| No | 37064 (52.1) | 984 (54.3) | 36785 (51.7) | 1005 (55.5) | 37102 (51.9) | 775 (54.1) | 37206 (52.0) | 755 (52.7) | 36192 (51.9) | 1688 (52.2) | 36262 (52.0) | 1696 (52.4) | 36489 (51.8) | 1306 (51.2) | 36744 (52.2) | 1299 (50.9) |
| Yes | 9765 (13.7) | 202 (11.1) | 9781 (13.7) | 211 (11.7) | 9904 (13.8) | 172 (12.0) | 9716 (13.6) | 167 (11.7) | 9538 (13.7) | 415 (12.8) | 9627 (13.8) | 379 (11.7) | 9594 (13.6) | 325 (12.7) | 9699 (13.8) | 341 (13.4) |
| Drinking** (%) |  |  |  |  |  |  |  |  |  |  |  |  |  |  |  |  |
| Ex | 3386 (4.8) | 115 (6.3) | 3613 (5.1) | 112 (6.2) | 3555 (5.0) | 80 (5.6) | 3506 (4.9) | 85 (5.9) | 3361 (4.8) | 243 (7.5) | 3400 (4.9) | 222 (6.9) | 3445 (4.9) | 178 (7.0) | 3440 (4.9) | 163 (6.4) |
| No | 20245 (28.4) | 642 (35.4) | 20254 (28.5) | 650 (35.9) | 20380 (28.5) | 513 (35.8) | 20406 (28.5) | 492 (34.3) | 19831 (28.4) | 1129 (34.9) | 19734 (28.3) | 1097 (33.9) | 20152 (28.6) | 850 (33.3) | 19923 (28.3) | 866 (33.9) |
| Yes | 47537 (66.8) | 1055 (58.2) | 47301 (66.5) | 1049 (57.9) | 47612 (66.5) | 840 (58.6) | 47634 (66.6) | 856 (59.7) | 46553 (66.7) | 1863 (57.6) | 46610 (66.8) | 1916 (59.2) | 46830 (66.5) | 1525 (59.7) | 47064 (66.8) | 1523 (59.7) |
| Diabetes type I*= 1 (%) | 154 (0.2) | 5 (0.3) | 134 (0.2) | 4 (0.2) | 152 (0.2) | 3 (0.2) | 137 (0.2) | 5 (0.3) | 133 (0.2) | 12 (0.4) | 147 (0.2) | 5 (0.2) | 135 (0.2) | 6 (0.2) | 151 (0.2) | 5 (0.2) |
| Diabetes type II*= 1 (%) | 4024 (5.7) | 157 (8.7) | 4008 (5.6) | 151 (8.3) | 4006 (5.6) | 128 (8.9) | 4065 (5.7) | 141 (9.8) | 3971 (5.7) | 286 (8.8) | 3832 (5.5) | 251 (7.8) | 3970 (5.6) | 217 (8.5) | 3921 (5.6) | 232 (9.1) |
| Chronic obstructive pulmonary disease*= 1 (%) | 5443 (7.6) | 171 (9.4) | 5481 (7.7) | 156 (8.6) | 5590 (7.8) | 125 (8.7) | 5383 (7.5) | 153 (10.7) | 5332 (7.6) | 286 (8.8) | 5355 (7.7) | 278 (8.6) | 5447 (7.7) | 231 (9.0) | 5330 (7.6) | 243 (9.5) |
| Chronic kidney disease*= 1 (%) | 6490 (9.1) | 243 (13.4) | 6408 (9.0) | 255 (14.1) | 6490 (9.1) | 207 (14.4) | 6498 (9.1) | 201 (14.0) | 6325 (9.1) | 442 (13.7) | 6183 (8.9) | 446 (13.8) | 6371 (9.0) | 376 (14.7) | 6301 (8.9) | 348 (13.6) |
| Rheumatoid arthritis*= 1 (%) | 9616 (13.5) | 191 (10.5) | 9747 (13.7) | 192 (10.6) | 9658 (13.5) | 177 (12.4) | 9739 (13.6) | 172 (12.0) | 9623 (13.8) | 379 (11.7) | 9357 (13.4) | 387 (12.0) | 9440 (13.4) | 331 (13.0) | 9626 (13.7) | 349 (13.7) |
| Lupus*= 1 (%) | 182 (0.3) | 1 (0.1) | 143 (0.2) | 2 (0.1) | 157 (0.2) | 0 (0.0) | 168 (0.2) | 3 (0.2) | 166 (0.2) | 4 (0.1) | 155 (0.2) | 3 (0.1) | 154 (0.2) | 3 (0.1) | 167 (0.2) | 4 (0.2) |
| Systemic heart disease**= 1 (%) | 2578 (3.6) | 15 (0.8) | 2610 (3.7) | 19 (1.0) | 2600 (3.6) | 15 (1.0) | 2593 (3.6) | 14 (1.0) | 2488 (3.6) | 44 (1.4) | 2654 (3.8) | 36 (1.1) | 2571 (3.7) | 32 (1.3) | 2580 (3.7) | 39 (1.5) |
| Heparin use**= 1 (%) | 504 (0.7) | 9 (0.5) | 513 (0.7) | 9 (0.5) | 526 (0.7) | 7 (0.5) | 494 (0.7) | 8 (0.6) | 461 (0.7) | 18 (0.6) | 532 (0.8) | 24 (0.7) | 495 (0.7) | 20 (0.8) | 508 (0.7) | 12 (0.5) |
| Beta-blocker use**= 1 (%) | 11046 (15.5) | 401 (22.1) | 10904 (15.3) | 402 (22.2) | 10994 (15.4) | 357 (24.9) | 11057 (15.5) | 345 (24.1) | 10492 (15.0) | 738 (22.8) | 10836 (15.5) | 687 (21.2) | 10662 (15.1) | 612 (24.0) | 10863 (15.4) | 616 (24.1) |
| Hypertension**= 1 (%) | 4614 (6.5) | 121 (6.7) | 4651 (6.5) | 128 (7.1) | 4709 (6.6) | 105 (7.3) | 4588 (6.4) | 112 (7.8) | 4501 (6.5) | 228 (7.0) | 4536 (6.5) | 249 (7.7) | 4555 (6.5) | 210 (8.2) | 4552 (6.5) | 197 (7.7) |
| Deep vein thrombosis or pulmonary embolism**= 1 (%) | 672 (0.9) | 16 (0.9) | 624 (0.9) | 22 (1.2) | 666 (0.9) | 19 (1.3) | 638 (0.9) | 11 (0.8) | 648 (0.9) | 33 (1.0) | 619 (0.9) | 34 (1.1) | 672 (1.0) | 32 (1.3) | 612 (0.9) | 18 (0.7) |
| Anticoagulant use**= 1 (%) | 3719 (5.2) | 136 (7.5) | 3548 (5.0) | 143 (7.9) | 3658 (5.1) | 123 (8.6) | 3664 (5.1) | 101 (7.0) | 3490 (5.0) | 262 (8.1) | 3534 (5.1) | 260 (8.0) | 3612 (5.1) | 209 (8.2) | 3524 (5.0) | 201 (7.9) |
| Antidepressants TCA**= 1 (%) | 6980 (9.8) | 172 (9.5) | 7117 (10.0) | 182 (10.0) | 7027 (9.8) | 156 (10.9) | 7104 (9.9) | 164 (11.4) | 6938 (9.9) | 328 (10.1) | 6870 (9.9) | 315 (9.7) | 6961 (9.9) | 282 (11.0) | 6927 (9.8) | 281 (11.0) |
| Antidepressants SSRI**= 1 (%) | 6098 (8.6) | 197 (10.9) | 6149 (8.6) | 207 (11.4) | 6133 (8.6) | 151 (10.5) | 6215 (8.7) | 152 (10.6) | 5958 (8.5) | 341 (10.5) | 5989 (8.6) | 363 (11.2) | 6044 (8.6) | 273 (10.7) | 6083 (8.6) | 251 (9.8) |
| Hypercholesterolemia**= 1 (%) | 1292 (1.8) | 27 (1.5) | 1200 (1.7) | 24 (1.3) | 1257 (1.8) | 20 (1.4) | 1243 (1.7) | 23 (1.6) | 1230 (1.8) | 43 (1.3) | 1223 (1.8) | 47 (1.5) | 1232 (1.7) | 37 (1.4) | 1239 (1.8) | 35 (1.4) |
| Statin use**= 1 (%) | 16557 (23.3) | 532 (29.4) | 16373 (23.0) | 526 (29.0) | 16554 (23.1) | 424 (29.6) | 16535 (23.1) | 475 (33.1) | 16086 (23.1) | 962 (29.7) | 15997 (22.9) | 943 (29.1) | 16186 (23.0) | 788 (30.9) | 16207 (23.0) | 807 (31.6) |
| Osteoporosis history* = 1 | 22732 (31.9) | 417 (23.0) | 22661 (31.8) | 405 (22.4) | 22827 (31.9) | 351 (24.5) | 22681 (31.7) | 356 (24.8) | 22330 (32.0) | 738 (22.8) | 22364 (32.1) | 783 (24.2) | 22511 (32.0) | 637 (25.0) | 22424 (31.8) | 643 (25.2) |
| Family history of cardiovascular disease = 1 (%) | 6478 (9.1) | 119 (6.6) | 6476 (9.1) | 131 (7.2) | 6462 (9.0) | 104 (7.3) | 6516 (9.1) | 122 (8.5) | 6326 (9.1) | 236 (7.3) | 6403 (9.2) | 239 (7.4) | 6455 (9.2) | 207 (8.1) | 6331 (9.0) | 211 (8.3) |
| Family history of cardiovascular disease before age 60= 1 (%) | 89 (0.1) | 0 (0.0) | 77 (0.1) | 4 (0.2) | 98 (0.1) | 1 (0.1) | 69 (0.1) | 2 (0.1) | 77 (0.1) | 1 (0.0) | 89 (0.1) | 3 (0.1) | 94 (0.1) | 2 (0.1) | 73 (0.1) | 1 (0.0) |
| Heart failure*= 1 (%) | 2415 (3.4) | 130 (7.2) | 2369 (3.3) | 126 (7.0) | 2428 (3.4) | 105 (7.3) | 2393 (3.3) | 114 (8.0) | 2327 (3.3) | 218 (6.7) | 2274 (3.3) | 221 (6.8) | 2378 (3.4) | 172 (6.7) | 2312 (3.3) | 178 (7.0) |
| Migraine*= 1 (%) | 9678 (13.6) | 221 (12.2) | 9832 (13.8) | 230 (12.7) | 9690 (13.5) | 194 (13.5) | 9860 (13.8) | 217 (15.1) | 9627 (13.8) | 412 (12.7) | 9539 (13.7) | 383 (11.8) | 9648 (13.7) | 354 (13.9) | 9611 (13.6) | 348 (13.6) |
| Severe mental illness*= 1 (%) | 9365 (13.2) | 230 (12.7) | 9437 (13.3) | 224 (12.4) | 9537 (13.3) | 182 (12.7) | 9348 (13.1) | 189 (13.2) | 9219 (13.2) | 411 (12.7) | 9203 (13.2) | 423 (13.1) | 9418 (13.4) | 334 (13.1) | 9168 (13.0) | 336 (13.2) |
| Vascular Disease*= 1 (%) | 832 (1.2) | 56 (3.1) | 841 (1.2) | 36 (2.0) | 830 (1.2) | 40 (2.8) | 859 (1.2) | 36 (2.5) | 773 (1.1) | 80 (2.5) | 833 (1.2) | 79 (2.4) | 796 (1.1) | 67 (2.6) | 841 (1.2) | 61 (2.4) |
| Atrial fibrillation*= 1 (%) | 3855 (5.4) | 203 (11.2) | 3750 (5.3) | 214 (11.8) | 3773 (5.3) | 171 (11.9) | 3908 (5.5) | 170 (11.9) | 3684 (5.3) | 343 (10.6) | 3643 (5.2) | 352 (10.9) | 3796 (5.4) | 255 (10.0) | 3665 (5.2) | 306 (12.0) |
| On anti-hypertensive drug= 1 (%) | 38540 (54.2) | 1157 (63.9) | 38219 (53.7) | 1128 (62.3) | 38610 (54.0) | 948 (66.2) | 38516 (53.8) | 970 (67.7) | 37252 (53.4) | 2043 (63.2) | 37645 (54.0) | 2104 (65.0) | 37816 (53.7) | 1739 (68.1) | 37815 (53.7) | 1674 (65.6) |
| Antipsychotic use**= 1 (%) | 361 (0.5) | 22 (1.2) | 379 (0.5) | 11 (0.6) | 372 (0.5) | 7 (0.5) | 386 (0.5) | 8 (0.6) | 370 (0.5) | 23 (0.7) | 354 (0.5) | 26 (0.8) | 394 (0.6) | 10 (0.4) | 357 (0.5) | 12 (0.5) |
| Steroid use**= 1 (%) | 18111 (25.4) | 445 (24.6) | 18181 (25.5) | 464 (25.6) | 18267 (25.5) | 379 (26.4) | 18159 (25.4) | 396 (27.6) | 17760 (25.5) | 842 (26.0) | 17820 (25.6) | 779 (24.1) | 17947 (25.5) | 686 (26.9) | 17883 (25.4) | 685 (26.8) |
| Erectile dysfunction**= 1 (%) | 1376 (1.9) | 40 (2.2) | 1343 (1.9) | 38 (2.1) | 1383 (1.9) | 41 (2.9) | 1342 (1.9) | 31 (2.2) | 1332 (1.9) | 68 (2.1) | 1323 (1.9) | 74 (2.3) | 1331 (1.9) | 60 (2.4) | 1340 (1.9) | 66 (2.6) |
| Age>75 | 37245 (52.3) | 1465 (80.8) | 37009 (52.0) | 1480 (81.7) | 37455 (52.4) | 1132 (79.0) | 37487 (52.4) | 1125 (78.5) | 36108 (51.8) | 2566 (79.3) | 35902 (51.5) | 2623 (81.1) | 36707 (52.1) | 1992 (78.0) | 36519 (51.9) | 1981 (77.6) |
| Age Group (%) |  |  |  |  |  |  |  |  |  |  |  |  |  |  |  |  |
| 50-59 | 8098 (11.4) | 43 (2.4) | 8200 (11.5) | 47 (2.6) | 8142 (11.4) | 37 (2.6) | 8164 (11.4) | 45 (3.1) | 8116 (11.6) | 78 (2.4) | 8116 (11.6) | 78 (2.4) | 8100 (11.5) | 68 (2.7) | 8147 (11.6) | 73 (2.9) |
| 60-69 | 15607 (21.9) | 153 (8.4) | 15748 (22.1) | 157 (8.7) | 15719 (22.0) | 129 (9.0) | 15676 (21.9) | 141 (9.8) | 15461 (22.2) | 296 (9.1) | 15632 (22.4) | 276 (8.5) | 15527 (22.0) | 253 (9.9) | 15641 (22.2) | 244 (9.6) |
| 70-79 | 22289 (31.3) | 465 (25.7) | 22485 (31.6) | 440 (24.3) | 22402 (31.3) | 383 (26.7) | 22496 (31.4) | 398 (27.8) | 21985 (31.5) | 876 (27.1) | 21995 (31.5) | 823 (25.4) | 22035 (31.3) | 702 (27.5) | 22187 (31.5) | 755 (29.6) |
| 80-89 | 20318 (28.5) | 847 (46.7) | 20028 (28.1) | 860 (47.5) | 20437 (28.6) | 687 (47.9) | 20284 (28.4) | 645 (45.0) | 19621 (28.1) | 1467 (45.3) | 19407 (27.8) | 1558 (48.2) | 19976 (28.4) | 1203 (47.1) | 19721 (28.0) | 1153 (45.2) |
| >89 | 4856 (6.8) | 304 (16.8) | 4707 (6.6) | 307 (17.0) | 4847 (6.8) | 197 (13.7) | 4926 (6.9) | 204 (14.2) | 4562 (6.5) | 518 (16.0) | 4594 (6.6) | 500 (15.5) | 4789 (6.8) | 327 (12.8) | 4731 (6.7) | 327 (12.8) |
| Charlson score (%) |  |  |  |  |  |  |  |  |  |  |  |  |  |  |  |  |
| 0 | 39525 (55.5) | 912 (50.3) | 39354 (55.3) | 926 (51.1) | 39531 (55.3) | 682 (47.6) | 39821 (55.7) | 683 (47.7) | 38612 (55.4) | 1640 (50.7) | 38847 (55.7) | 1618 (50.0) | 39020 (55.4) | 1227 (48.1) | 39251 (55.7) | 1219 (47.8) |
| 1 | 15190 (21.3) | 340 (18.8) | 15256 (21.4) | 346 (19.1) | 15345 (21.4) | 294 (20.5) | 15213 (21.3) | 280 (19.5) | 14961 (21.5) | 648 (20.0) | 14878 (21.3) | 645 (19.9) | 15033 (21.3) | 525 (20.6) | 15030 (21.3) | 544 (21.3) |
| 2 | 8803 (12.4) | 243 (13.4) | 8848 (12.4) | 247 (13.6) | 8938 (12.5) | 200 (14.0) | 8789 (12.3) | 214 (14.9) | 8709 (12.5) | 405 (12.5) | 8566 (12.3) | 461 (14.3) | 8798 (12.5) | 373 (14.6) | 8616 (12.2) | 354 (13.9) |
| ≥3 | 7650 (10.7) | 317 (17.5) | 7710 (10.8) | 292 (16.1) | 7733 (10.8) | 257 (17.9) | 7723 (10.8) | 256 (17.9) | 7463 (10.7) | 542 (16.8) | 7453 (10.7) | 511 (15.8) | 7576 (10.8) | 428 (16.8) | 7530 (10.7) | 435 (17.0) |
| Cardiovascular disease (%) |  |  |  |  |  |  |  |  |  |  |  |  |  |  |  |  |
| No | 62945 (88.4) | 1389 (76.7) | 62804 (88.2) | 1378 (76.1) | 63095 (88.2) | 1061 (74.0) | 63297 (88.5) | 1063 (74.2) | 61818 (88.6) | 2495 (77.1) | 61689 (88.5) | 2514 (77.7) | 62317 (88.5) | 1938 (75.9) | 62338 (88.5) | 1923 (75.4) |
| Ever >1 year before index date | 6397 (9.0) | 289 (15.9) | 6487 (9.1) | 301 (16.6) | 6545 (9.1) | 253 (17.7) | 6422 (9.0) | 254 (17.7) | 6176 (8.9) | 535 (16.5) | 6263 (9.0) | 500 (15.5) | 6272 (8.9) | 453 (17.7) | 6317 (9.0) | 432 (16.9) |
| 1 year before index | 728 (1.0) | 46 (2.5) | 745 (1.0) | 32 (1.8) | 792 (1.1) | 30 (2.1) | 692 (1.0) | 37 (2.6) | 685 (1.0) | 71 (2.2) | 729 (1.0) | 66 (2.0) | 745 (1.1) | 61 (2.4) | 695 (1.0) | 50 (2.0) |
| 6 months before index | 804 (1.1) | 57 (3.1) | 834 (1.2) | 68 (3.8) | 823 (1.2) | 61 (4.3) | 829 (1.2) | 50 (3.5) | 764 (1.1) | 98 (3.0) | 799 (1.1) | 102 (3.2) | 799 (1.1) | 68 (2.7) | 792 (1.1) | 104 (4.1) |
| 1 month before index | 294 (0.4) | 31 (1.7) | 298 (0.4) | 32 (1.8) | 292 (0.4) | 28 (2.0) | 306 (0.4) | 29 (2.0) | 302 (0.4) | 36 (1.1) | 264 (0.4) | 53 (1.6) | 294 (0.4) | 33 (1.3) | 285 (0.4) | 43 (1.7) |
| MI or Stroke (%) |  |  |  |  |  |  |  |  |  |  |  |  |  |  |  |  |
| No | 66305 (93.2) | 1407 (77.6) | 66192 (93.0) | 1431 (79.0) | 66458 (92.9) | 1161 (81.0) | 66585 (93.1) | 1131 (78.9) | 65070 (93.3) | 2589 (80.0) | 65106 (93.3) | 2570 (79.4) | 65613 (93.2) | 2083 (81.6) | 65565 (93.1) | 2074 (81.3) |
| Ever >1 year before index date | 3472 (4.9) | 235 (13.0) | 3550 (5.0) | 224 (12.4) | 3624 (5.1) | 162 (11.3) | 3518 (4.9) | 177 (12.4) | 3399 (4.9) | 399 (12.3) | 3271 (4.7) | 412 (12.7) | 3390 (4.8) | 304 (11.9) | 3497 (5.0) | 290 (11.4) |
| 1 year before index | 1391 (2.0) | 170 (9.4) | 1426 (2.0) | 156 (8.6) | 1465 (2.0) | 110 (7.7) | 1443 (2.0) | 125 (8.7) | 1276 (1.8) | 247 (7.6) | 1367 (2.0) | 253 (7.8) | 1424 (2.0) | 166 (6.5) | 1365 (1.9) | 188 (7.4) |
| Established CVD *= Ever (%) | 8724 (12.3) | 586 (32.3) | 8890 (12.5) | 574 (31.7) | 9049 (12.6) | 432 (30.1) | 8847 (12.4) | 446 (31.1) | 8383 (12.0) | 988 (30.5) | 8432 (12.1) | 971 (30.0) | 8570 (12.2) | 733 (28.7) | 8725 (12.4) | 746 (29.2) |
| Any fracture history (%) |  |  |  |  |  |  |  |  |  |  |  |  |  |  |  |  |
| No | 55890 (78.5) | 1347 (74.3) | 55766 (78.4) | 1383 (76.4) | 56065 (78.4) | 1076 (75.1) | 56182 (78.5) | 1063 (74.2) | 54753 (78.5) | 2449 (75.7) | 54757 (78.5) | 2427 (75.0) | 55277 (78.5) | 1895 (74.2) | 55268 (78.5) | 1946 (76.3) |
| Ever >1 year before index date | 5128 (7.2) | 139 (7.7) | 5197 (7.3) | 134 (7.4) | 5167 (7.2) | 113 (7.9) | 5198 (7.3) | 120 (8.4) | 5022 (7.2) | 258 (8.0) | 5085 (7.3) | 233 (7.2) | 5082 (7.2) | 204 (8.0) | 5114 (7.3) | 198 (7.8) |
| 1 year before index | 10150 (14.3) | 326 (18.0) | 10205 (14.3) | 294 (16.2) | 10315 (14.4) | 244 (17.0) | 10166 (14.2) | 250 (17.4) | 9970 (14.3) | 528 (16.3) | 9902 (14.2) | 575 (17.8) | 10068 (14.3) | 454 (17.8) | 10045 (14.3) | 408 (16.0) |
| Hip fracture history (%) |  |  |  |  |  |  |  |  |  |  |  |  |  |  |  |  |
| No | 66623 (93.6) | 1637 (90.3) | 66709 (93.7) | 1641 (90.6) | 66951 (93.6) | 1297 (90.5) | 67050 (93.7) | 1312 (91.6) | 65416 (93.8) | 2927 (90.5) | 65351 (93.7) | 2916 (90.1) | 66012 (93.7) | 2307 (90.4) | 65959 (93.7) | 2332 (91.4) |
| Ever >1 year before index date | 808 (1.1) | 34 (1.9) | 844 (1.2) | 34 (1.9) | 843 (1.2) | 32 (2.2) | 822 (1.1) | 23 (1.6) | 794 (1.1) | 66 (2.0) | 804 (1.2) | 56 (1.7) | 799 (1.1) | 46 (1.8) | 833 (1.2) | 42 (1.6) |
| 1 year before index | 3737 (5.3) | 141 (7.8) | 3615 (5.1) | 136 (7.5) | 3753 (5.2) | 104 (7.3) | 3674 (5.1) | 98 (6.8) | 3535 (5.1) | 242 (7.5) | 3589 (5.1) | 263 (8.1) | 3616 (5.1) | 200 (7.8) | 3635 (5.2) | 178 (7.0) |
| Shoulder fracture history (%) |  |  |  |  |  |  |  |  |  |  |  |  |  |  |  |  |
| No | 70809 (99.5) | 1800 (99.3) | 70787 (99.5) | 1803 (99.6) | 71214 (99.5) | 1421 (99.2) | 71140 (99.4) | 1424 (99.4) | 69362 (99.5) | 3221 (99.6) | 69405 (99.5) | 3211 (99.3) | 70063 (99.5) | 2532 (99.2) | 70066 (99.5) | 2538 (99.5) |
| Ever >1 year before index date | 168 (0.2) | 3 (0.2) | 159 (0.2) | 3 (0.2) | 146 (0.2) | 2 (0.1) | 182 (0.3) | 3 (0.2) | 164 (0.2) | 5 (0.2) | 154 (0.2) | 10 (0.3) | 162 (0.2) | 8 (0.3) | 158 (0.2) | 5 (0.2) |
| 1 year before index | 191 (0.3) | 9 (0.5) | 222 (0.3) | 5 (0.3) | 187 (0.3) | 10 (0.7) | 224 (0.3) | 6 (0.4) | 219 (0.3) | 9 (0.3) | 185 (0.3) | 14 (0.4) | 202 (0.3) | 13 (0.5) | 203 (0.3) | 9 (0.4) |
| Spine fracture history (%) |  |  |  |  |  |  |  |  |  |  |  |  |  |  |  |  |
| No | 70030 (98.4) | 1776 (98.0) | 69977 (98.3) | 1771 (97.8) | 70385 (98.4) | 1400 (97.7) | 70368 (98.4) | 1401 (97.8) | 68591 (98.3) | 3180 (98.3) | 68622 (98.4) | 3161 (97.7) | 69274 (98.4) | 2496 (97.8) | 69280 (98.4) | 2504 (98.1) |
| Ever >1 year before index date | 173 (0.2) | 6 (0.3) | 169 (0.2) | 8 (0.4) | 164 (0.2) | 6 (0.4) | 180 (0.3) | 6 (0.4) | 161 (0.2) | 7 (0.2) | 176 (0.3) | 12 (0.4) | 175 (0.2) | 12 (0.5) | 164 (0.2) | 5 (0.2) |
| 1 year before index | 965 (1.4) | 30 (1.7) | 1022 (1.4) | 32 (1.8) | 998 (1.4) | 27 (1.9) | 998 (1.4) | 26 (1.8) | 993 (1.4) | 48 (1.5) | 946 (1.4) | 62 (1.9) | 978 (1.4) | 45 (1.8) | 983 (1.4) | 43 (1.7) |
| Wrist fracture history (%) |  |  |  |  |  |  |  |  |  |  |  |  |  |  |  |  |
| No | 66880 (94.0) | 1713 (94.5) | 66803 (93.9) | 1722 (95.1) | 67187 (93.9) | 1351 (94.3) | 67225 (94.0) | 1355 (94.6) | 65473 (93.9) | 3059 (94.6) | 65524 (93.9) | 3062 (94.7) | 66106 (93.9) | 2388 (93.5) | 66203 (94.0) | 2421 (94.9) |
| Ever >1 year before index date | 1963 (2.8) | 46 (2.5) | 1987 (2.8) | 44 (2.4) | 1949 (2.7) | 42 (2.9) | 2014 (2.8) | 35 (2.4) | 1985 (2.8) | 89 (2.8) | 1886 (2.7) | 80 (2.5) | 2000 (2.8) | 83 (3.3) | 1899 (2.7) | 58 (2.3) |
| 1 year before index | 2325 (3.3) | 53 (2.9) | 2378 (3.3) | 45 (2.5) | 2411 (3.4) | 40 (2.8) | 2307 (3.2) | 43 (3.0) | 2287 (3.3) | 87 (2.7) | 2334 (3.3) | 93 (2.9) | 2321 (3.3) | 82 (3.2) | 2325 (3.3) | 73 (2.9) |
| BMI** (%) |  |  |  |  |  |  |  |  |  |  |  |  |  |  |  |  |
| <18.5 | 5194 (7.3) | 248 (13.7) | 5206 (7.3) | 229 (12.6) | 5269 (7.4) | 160 (11.2) | 5288 (7.4) | 160 (11.2) | 4995 (7.2) | 357 (11.0) | 5144 (7.4) | 381 (11.8) | 5135 (7.3) | 234 (9.2) | 5239 (7.4) | 269 (10.5) |
| 18.6 - 24.9 | 32370 (45.5) | 902 (49.8) | 32352 (45.5) | 918 (50.7) | 32519 (45.5) | 704 (49.1) | 32649 (45.6) | 670 (46.8) | 31650 (45.4) | 1616 (50.0) | 31653 (45.4) | 1623 (50.2) | 32017 (45.5) | 1194 (46.8) | 32076 (45.5) | 1255 (49.2) |
| 25 - 29.9 | 21582 (30.3) | 429 (23.7) | 21631 (30.4) | 467 (25.8) | 21755 (30.4) | 373 (26.0) | 21571 (30.1) | 410 (28.6) | 21257 (30.5) | 833 (25.7) | 21202 (30.4) | 817 (25.3) | 21354 (30.3) | 735 (28.8) | 21347 (30.3) | 673 (26.4) |
| 30 - 39.9 | 11060 (15.5) | 218 (12.0) | 10987 (15.4) | 187 (10.3) | 11024 (15.4) | 186 (13.0) | 11064 (15.5) | 178 (12.4) | 10872 (15.6) | 400 (12.4) | 10789 (15.5) | 391 (12.1) | 10971 (15.6) | 368 (14.4) | 10784 (15.3) | 329 (12.9) |
| >=40 | 962 (1.4) | 15 (0.8) | 992 (1.4) | 10 (0.6) | 980 (1.4) | 10 (0.7) | 974 (1.4) | 15 (1.0) | 971 (1.4) | 29 (0.9) | 956 (1.4) | 23 (0.7) | 950 (1.3) | 22 (0.9) | 981 (1.4) | 26 (1.0) |
| No. of GP visits** (%) |  |  |  |  |  |  |  |  |  |  |  |  |  |  |  |  |
| 0 | 7330 (10.3) | 259 (14.3) | 7350 (10.3) | 238 (13.1) | 7338 (10.3) | 167 (11.7) | 7512 (10.5) | 160 (11.2) | 7287 (10.4) | 441 (13.6) | 7054 (10.1) | 395 (12.2) | 7359 (10.4) | 256 (10.0) | 7255 (10.3) | 307 (12.0) |
| 1-5 | 17739 (24.9) | 385 (21.2) | 17686 (24.9) | 403 (22.3) | 17828 (24.9) | 281 (19.6) | 17815 (24.9) | 289 (20.2) | 17315 (24.8) | 701 (21.7) | 17484 (25.1) | 713 (22.0) | 17498 (24.8) | 540 (21.2) | 17666 (25.1) | 509 (19.9) |
| 6-10 | 15800 (22.2) | 303 (16.7) | 15913 (22.4) | 328 (18.1) | 16021 (22.4) | 260 (18.1) | 15792 (22.1) | 271 (18.9) | 15625 (22.4) | 585 (18.1) | 15539 (22.3) | 595 (18.4) | 15576 (22.1) | 468 (18.3) | 15798 (22.4) | 502 (19.7) |
| 11-15 | 12035 (16.9) | 282 (15.6) | 11878 (16.7) | 271 (15.0) | 12149 (17.0) | 229 (16.0) | 11870 (16.6) | 218 (15.2) | 11756 (16.9) | 487 (15.1) | 11700 (16.8) | 523 (16.2) | 11690 (16.6) | 436 (17.1) | 11951 (17.0) | 389 (15.2) |
| >=16 | 18264 (25.7) | 583 (32.2) | 18341 (25.8) | 571 (31.5) | 18211 (25.5) | 496 (34.6) | 18557 (25.9) | 495 (34.5) | 17762 (25.5) | 1021 (31.6) | 17967 (25.8) | 1009 (31.2) | 18304 (26.0) | 853 (33.4) | 17757 (25.2) | 845 (33.1) |
| No. of GP emergency visits** (%) |  |  |  |  |  |  |  |  |  |  |  |  |  |  |  |  |
| 0 | 58545 (82.3) | 1296 (71.5) | 58536 (82.3) | 1321 (72.9) | 58771 (82.1) | 1033 (72.1) | 58859 (82.3) | 1035 (72.2) | 57400 (82.3) | 2384 (73.7) | 57541 (82.5) | 2373 (73.4) | 57934 (82.3) | 1877 (73.5) | 58001 (82.4) | 1886 (73.9) |
| 1 | 6791 (9.5) | 232 (12.8) | 6853 (9.6) | 207 (11.4) | 6870 (9.6) | 186 (13.0) | 6855 (9.6) | 172 (12.0) | 6713 (9.6) | 373 (11.5) | 6596 (9.5) | 401 (12.4) | 6807 (9.7) | 308 (12.1) | 6657 (9.5) | 311 (12.2) |
| 2 | 2581 (3.6) | 98 (5.4) | 2500 (3.5) | 103 (5.7) | 2588 (3.6) | 89 (6.2) | 2523 (3.5) | 82 (5.7) | 2435 (3.5) | 183 (5.7) | 2491 (3.6) | 173 (5.3) | 2466 (3.5) | 150 (5.9) | 2523 (3.6) | 143 (5.6) |
| 3-5 | 2401 (3.4) | 108 (6.0) | 2373 (3.3) | 111 (6.1) | 2427 (3.4) | 66 (4.6) | 2405 (3.4) | 95 (6.6) | 2312 (3.3) | 190 (5.9) | 2306 (3.3) | 185 (5.7) | 2347 (3.3) | 142 (5.6) | 2362 (3.4) | 142 (5.6) |
| >=6 | 850 (1.2) | 78 (4.3) | 906 (1.3) | 69 (3.8) | 891 (1.2) | 59 (4.1) | 904 (1.3) | 49 (3.4) | 885 (1.3) | 105 (3.2) | 810 (1.2) | 103 (3.2) | 873 (1.2) | 76 (3.0) | 884 (1.3) | 70 (2.7) |
| eGFR** (%) |  |  |  |  |  |  |  |  |  |  |  |  |  |  |  |  |
| <=29 | 871 (1.2) | 39 (2.2) | 825 (1.2) | 42 (2.3) | 841 (1.2) | 31 (2.2) | 869 (1.2) | 36 (2.5) | 801 (1.1) | 69 (2.1) | 826 (1.2) | 81 (2.5) | 823 (1.2) | 55 (2.2) | 829 (1.2) | 70 (2.7) |
| 30 - 44 | 4700 (6.6) | 206 (11.4) | 4631 (6.5) | 212 (11.7) | 4755 (6.6) | 178 (12.4) | 4648 (6.5) | 168 (11.7) | 4540 (6.5) | 389 (12.0) | 4417 (6.3) | 403 (12.5) | 4527 (6.4) | 333 (13.0) | 4601 (6.5) | 288 (11.3) |
| 45 - 59 | 14678 (20.6) | 513 (28.3) | 14590 (20.5) | 509 (28.1) | 14826 (20.7) | 421 (29.4) | 14640 (20.5) | 403 (28.1) | 14359 (20.6) | 883 (27.3) | 14170 (20.3) | 878 (27.1) | 14481 (20.6) | 730 (28.6) | 14384 (20.4) | 695 (27.2) |
| 60 - 89 | 47272 (66.4) | 999 (55.1) | 47572 (66.8) | 995 (54.9) | 47447 (66.3) | 763 (53.2) | 47845 (66.9) | 783 (54.6) | 46485 (66.6) | 1795 (55.5) | 46787 (67.1) | 1771 (54.7) | 47021 (66.8) | 1361 (53.3) | 47038 (66.8) | 1418 (55.6) |
| >=90 | 3647 (5.1) | 55 (3.0) | 3550 (5.0) | 53 (2.9) | 3678 (5.1) | 40 (2.8) | 3544 (5.0) | 43 (3.0) | 3560 (5.1) | 99 (3.1) | 3544 (5.1) | 102 (3.2) | 3575 (5.1) | 74 (2.9) | 3575 (5.1) | 81 (3.2) |
| SBP** (%) |  |  |  |  |  |  |  |  |  |  |  |  |  |  |  |  |
| <120 | 9053 (12.7) | 222 (12.3) | 9071 (12.7) | 203 (11.2) | 9057 (12.7) | 162 (11.3) | 9157 (12.8) | 173 (12.1) | 8988 (12.9) | 358 (11.1) | 8839 (12.7) | 364 (11.3) | 8842 (12.6) | 256 (10.0) | 9168 (13.0) | 283 (11.1) |
| 120 - 139 | 28165 (39.6) | 642 (35.4) | 28219 (39.7) | 659 (36.4) | 28382 (39.7) | 500 (34.9) | 28294 (39.5) | 509 (35.5) | 27539 (39.5) | 1200 (37.1) | 27821 (39.9) | 1125 (34.8) | 28015 (39.8) | 922 (36.1) | 27831 (39.5) | 917 (35.9) |
| 140 - 159 | 25143 (35.3) | 676 (37.3) | 25126 (35.3) | 644 (35.6) | 25230 (35.3) | 520 (36.3) | 25312 (35.4) | 527 (36.8) | 24602 (35.3) | 1161 (35.9) | 24611 (35.3) | 1215 (37.6) | 25088 (35.6) | 974 (38.2) | 24614 (34.9) | 913 (35.8) |
| >=160 | 8807 (12.4) | 272 (15.0) | 8752 (12.3) | 305 (16.8) | 8878 (12.4) | 251 (17.5) | 8783 (12.3) | 224 (15.6) | 8616 (12.4) | 516 (16.0) | 8473 (12.1) | 531 (16.4) | 8482 (12.0) | 401 (15.7) | 8814 (12.5) | 439 (17.2) |
| DBP** (%) |  |  |  |  |  |  |  |  |  |  |  |  |  |  |  |  |
| <80 | 36582 (51.4) | 993 (54.8) | 36535 (51.3) | 1022 (56.4) | 36689 (51.3) | 798 (55.7) | 36858 (51.5) | 787 (54.9) | 35762 (51.3) | 1766 (54.6) | 35797 (51.3) | 1807 (55.9) | 36117 (51.3) | 1400 (54.8) | 36222 (51.4) | 1393 (54.6) |
| 80 - 89 | 26337 (37.0) | 605 (33.4) | 26284 (36.9) | 572 (31.6) | 26469 (37.0) | 468 (32.7) | 26398 (36.9) | 463 (32.3) | 25697 (36.8) | 1099 (34.0) | 25943 (37.2) | 1059 (32.7) | 26145 (37.1) | 877 (34.4) | 25931 (36.8) | 845 (33.1) |
| 90 - 99 | 6592 (9.3) | 157 (8.7) | 6758 (9.5) | 171 (9.4) | 6718 (9.4) | 124 (8.7) | 6695 (9.4) | 141 (9.8) | 6663 (9.6) | 290 (9.0) | 6447 (9.2) | 278 (8.6) | 6607 (9.4) | 204 (8.0) | 6617 (9.4) | 250 (9.8) |
| >=100 | 1657 (2.3) | 57 (3.1) | 1591 (2.2) | 46 (2.5) | 1671 (2.3) | 43 (3.0) | 1595 (2.2) | 42 (2.9) | 1623 (2.3) | 80 (2.5) | 1557 (2.2) | 91 (2.8) | 1558 (2.2) | 72 (2.8) | 1657 (2.4) | 64 (2.5) |
| No. of concomitant medicines** (%) |  |  |  |  |  |  |  |  |  |  |  |  |  |  |  |  |
| 0 | 10025 (14.1) | 317 (17.5) | 10026 (14.1) | 294 (16.2) | 9996 (14.0) | 205 (14.3) | 10260 (14.3) | 201 (14.0) | 9911 (14.2) | 553 (17.1) | 9708 (13.9) | 490 (15.1) | 9962 (14.1) | 336 (13.2) | 9986 (14.2) | 378 (14.8) |
| 1 - 3 | 13032 (18.3) | 197 (10.9) | 13232 (18.6) | 225 (12.4) | 13213 (18.5) | 156 (10.9) | 13157 (18.4) | 160 (11.2) | 13013 (18.7) | 358 (11.1) | 12918 (18.5) | 397 (12.3) | 13035 (18.5) | 289 (11.3) | 13074 (18.6) | 288 (11.3) |
| 4 - 6 | 14290 (20.1) | 254 (14.0) | 14329 (20.1) | 252 (13.9) | 14363 (20.1) | 211 (14.7) | 14363 (20.1) | 188 (13.1) | 14041 (20.1) | 452 (14.0) | 14126 (20.3) | 506 (15.6) | 14092 (20.0) | 398 (15.6) | 14250 (20.2) | 385 (15.1) |
| 7 - 9 | 12823 (18.0) | 332 (18.3) | 12597 (17.7) | 297 (16.4) | 12869 (18.0) | 252 (17.6) | 12670 (17.7) | 258 (18.0) | 12454 (17.9) | 564 (17.4) | 12443 (17.8) | 588 (18.2) | 12628 (17.9) | 464 (18.2) | 12507 (17.8) | 450 (17.6) |
| 10 - 12 | 9184 (12.9) | 276 (15.2) | 9106 (12.8) | 302 (16.7) | 9140 (12.8) | 241 (16.8) | 9251 (12.9) | 236 (16.5) | 8878 (12.7) | 531 (16.4) | 8966 (12.9) | 493 (15.2) | 9017 (12.8) | 420 (16.5) | 9016 (12.8) | 415 (16.3) |
| >=13 | 11814 (16.6) | 436 (24.1) | 11878 (16.7) | 441 (24.4) | 11966 (16.7) | 368 (25.7) | 11845 (16.6) | 390 (27.2) | 11448 (16.4) | 777 (24.0) | 11583 (16.6) | 761 (23.5) | 11693 (16.6) | 646 (25.3) | 11594 (16.5) | 636 (24.9) |
| Cholesterol measurement** (HDL/LDL) (%) |  |  |  |  |  |  |  |  |  |  |  |  |  |  |  |  |
| <=3.5 | 42161 (59.2) | 1040 (57.4) | 41958 (59.0) | 1088 (60.1) | 42183 (59.0) | 850 (59.3) | 42367 (59.2) | 847 (59.1) | 41201 (59.1) | 1949 (60.2) | 41217 (59.1) | 1880 (58.1) | 41788 (59.3) | 1525 (59.7) | 41410 (58.8) | 1524 (59.7) |
| 3.6 - 5 | 22710 (31.9) | 602 (33.2) | 22794 (32.0) | 548 (30.3) | 22896 (32.0) | 446 (31.1) | 22867 (32.0) | 445 (31.1) | 22336 (32.0) | 986 (30.5) | 22311 (32.0) | 1021 (31.6) | 22364 (31.8) | 757 (29.7) | 22748 (32.3) | 785 (30.8) |
| >5 | 6297 (8.8) | 170 (9.4) | 6416 (9.0) | 175 (9.7) | 6468 (9.0) | 137 (9.6) | 6312 (8.8) | 141 (9.8) | 6208 (8.9) | 300 (9.3) | 6216 (8.9) | 334 (10.3) | 6275 (8.9) | 271 (10.6) | 6269 (8.9) | 243 (9.5) |
| No. of previous fractures* (%) |  |  |  |  |  |  |  |  |  |  |  |  |  |  |  |  |
| 0 | 54809 (77.0) | 1323 (73.0) | 54649 (76.8) | 1339 (73.9) | 55028 (76.9) | 1044 (72.9) | 55008 (76.9) | 1040 (72.6) | 53668 (76.9) | 2392 (73.9) | 53681 (77.0) | 2379 (73.5) | 54207 (77.0) | 1849 (72.4) | 54160 (76.9) | 1904 (74.6) |
| 1 | 8772 (12.3) | 256 (14.1) | 8835 (12.4) | 266 (14.7) | 8802 (12.3) | 215 (15.0) | 8899 (12.4) | 213 (14.9) | 8597 (12.3) | 453 (14.0) | 8622 (12.4) | 457 (14.1) | 8704 (12.4) | 364 (14.3) | 8693 (12.3) | 368 (14.4) |
| >=2 | 7587 (10.7) | 233 (12.9) | 7684 (10.8) | 206 (11.4) | 7717 (10.8) | 174 (12.1) | 7639 (10.7) | 180 (12.6) | 7480 (10.7) | 390 (12.1) | 7441 (10.7) | 399 (12.3) | 7516 (10.7) | 340 (13.3) | 7574 (10.8) | 280 (11.0) |
| **Abbreviations:** OST, patients with incident diagnosis of osteoporosis; IFX, patients with incident fragility fracture; OBP, incident users of oral bisphosphonates; MACE, composite outcome for the occurrence of either myocardial infarction, stroke or cardiovascular disease death; MI, myocardial infarction; * ever; ** in the year prior to start; SES, socio-economic status; BMI, body mass index; eGFR, estimated Glomerular Filtration Rate; SBP, cholesterol, systolic blood pressure; DBP, diastolic blood pressure. | | | | | | | | | | | | | | | | |

# Table S3 Model Equations of one-year MACE (risk factors selected by lasso regression)

| Predictor | OST Beta coefficients | IFX Beta coefficients | OBP Beta coefficients |
| --- | --- | --- | --- |
| Intercept | -5.214 | -6.458 | -4.416 |
| Sex = Male (%) | 0.479 | 0.257 | 0.349 |
| SES (%) | x |  |  |
| 1 | x | x | ref |
| 2 | x | x | 0.160 |
| 3 | x | x | 0.248 |
| 4 | x | x | 0.293 |
| 5 | x | x | 0.256 |
| Smoking** |  | x |  |
| Ex | ref | ref | ref |
| No | -0.070 | 0.003 | -0.016 |
| Yes | 0.353 | 0.148 | 0.134 |
| Drinking** |  | x |  |
| Ex | ref | ref | ref |
| No | 0.171 | 0.132 | 0.025 |
| Yes | -0.170 | 0.109 | -0.084 |
| Diabetes type I* | x | x | x |
| Diabetes type II* | x | x | 0.101 |
| Chronic obstructive pulmonary disease* | x | x | 0.048 |
| Chronic kidney disease* | x | x | -0.216 |
| Rheumatoid arthritis* | x | x | -0.127 |
| Lupus* | x | x | x |
| Systemic heart disease** | x | x | -0.479 |
| Anti-osteoporosis use** | x | x | x |
| Heparin use** | x | x | x |
| Beta-blocker use** | x | 0.104 | 0.149 |
| Hypertension** | x | x | x |
| Deep vein thrombosis or pulmonary embolism** | x | x | x |
| Anticoagulant use** | x | x | -0.078 |
| Antidepressants TCA** | x | x | x |
| Antidepressants SSRI** | x |  | 0.243 |
| Hypercholesterolemia** | x | x | x |
| Statin use** | x |  | x |
| Osteoporosis history* | x | x | -0.301 |
| Family history of cardiovascular disease (%) | x | x | -0.058 |
| Family history of cardiovascular disease before age 60 | x | x | x |
| Heart failure* | x | x | 0.031 |
| Migraine* | x | x | x |
| Severe mental illness* | x | x | x |
| Vascular Disease* | X | x | x |
| Atrial fibrillation* | 0.478 | 0.182 | 0.258 |
| On anti-hypertensive drug | 0.203 | 0.073 | 0.047 |
| Antipsychotic use** | x | x | x |
| Steroid use** | x | x | x |
| Erectile dysfunction** | x | x | x |
| Age Group (%) |  |  |  |
| 50-59 | ref | ref | ref |
| 60-69 | 0.311 | 2.036 | 0.457 |
| 70-79 | 1.122 | 2.531 | 0.899 |
| 80-89 | 1.589 | 3.015 | 1.449 |
| >89 | 2.065 | 3.302 | 1.756 |
| Charlson score | x | x |  |
| 0 | x | x | ref |
| 1 | x | x | 0.012 |
| 2 | x | x | -0.059 |
| ≥3 | x | x | 0.055 |
| Cardiovascular disease | x | x |  |
| No | x | x | ref |
| Ever >1 year before index date | x | x | 0.152 |
| 1 year before index | x | x | 0.166 |
| 6 months before index | x | x | 0.420 |
| 1 month before index | x | x | 0.781 |
| MI or Stroke |  |  |  |
| No | ref | ref | ref |
| Ever >1 year before index date | -0.009 | 0.206 | 0.290 |
| 1 year before index | 0.709 | 0.443 | 0.925 |
| Established CVD * | 0.643 | 0.533 | 0.401 |
| Any fracture history | x | x | x |
| No | x | x | x |
| Ever >1 year before index date | x | x | x |
| 1 year before index | x | x | x |
| Hip fracture history | x | x | x |
| No | x | x | x |
| Ever >1 year before index date | x | x | x |
| 1 year before index | x | x | x |
| Shoulder fracture history | x | x | x |
| No | x | x | x |
| Ever >1 year before index date | x | x | x |
| 1 year before index | x | x | x |
| Spine fracture history | x | x | x |
| No | x | x | x |
| Ever >1 year before index date | x | x | x |
| 1 year before index | x | x | x |
| Wrist fracture history | x | x | x |
| No | x | x | x |
| Ever >1 year before index date | x | x | x |
| 1 year before index | x | x | x |
| BMI** |  | x |  |
| <18.5 | ref | x | ref |
| 18.6 - 24.9 | -0.334 | x | -0.302 |
| 25 - 29.9 | -0.581 | x | -0.546 |
| 30 - 39.9 | -0.879 | x | -0.601 |
| >=40 | -0.421 | x | -0.946 |
| No. of GP visits** |  | x |  |
| 0 | ref | x | ref |
| 1-5 | 0.009 | x | -0.109 |
| 6-10 | -0.063 | x | -0.243 |
| 11-15 | -0.056 | x | -0.252 |
| >=16 | 0.064 | x | -0.146 |
| No. of GP emergency visits** | x |  |  |
| 0 | x | ref | ref |
| 1 | x | 0.220 | 0.143 |
| 2 | x | 0.358 | 0.339 |
| 3-5 | x | 0.190 | 0.384 |
| >=6 | x | 0.122 | 0.928 |
| eGFR** |  |  |  |
| <=29 | ref | ref | ref |
| 30 – 44 | 0.101 | -0.139 | -0.089 |
| 45 – 59 | -0.112 | -0.363 | -0.190 |
| 60 – 89 | -0.280 | -0.524 | -0.541 |
| >=90 | -0.236 | -0.661 | -0.687 |
| SBP** |  |  |  |
| <120 | ref | ref | ref |
| 120 - 139 | 0.175 | 0.041 | 0.090 |
| 140 - 159 | 0.278 | 0.181 | 0.216 |
| >=160 | 0.274 | 0.430 | 0.329 |
| DBP** | x |  |  |
| <80 | x | ref | ref |
| 80 - 89 | x | 0.037 | -0.058 |
| 90 - 99 | x | 0.167 | 0.050 |
| >=100 | x | 0.121 | 0.271 |
| No. of concomitant medicines** |  |  |  |
| 0 | ref | ref | ref |
| 1 – 3 | -0.377 | 0.134 | -0.142 |
| 4 – 6 | -0.183 | 0.257 | -0.287 |
| 7 – 9 | 0.008 | 0.110 | -0.128 |
| 10 – 12 | 0.111 | 0.233 | -0.001 |
| >=13 | 0.105 | 0.226 | -0.020 |
| Cholesterol measurement** (HDL/LDL) | x | x |  |
| <=3.5 | x | x | ref |
| 3.6 – 5 | x | 0.068 | 0.180 |
| >5 | x | 0.431 | 0.299 |
| No. of previous fractures* |  | x | x |
| 0 | ref | x | x |
| 1 | 0.217 | x | x |
| >=2 | -0.008 | x | x |
| Abbreviations: OST, patients with incident diagnosis of osteoporosis; IFX, patients with incident fragility fracture; OBP, incident users of oral bisphosphonates; OR, odds ratio; CI, confidence intervals; MACE, composite outcome for the occurrence of either myocardial infarction, stroke or cardiovascular disease death; * ever; ** in the year prior to start; SES, socio-economic status; MI, myocardial infarction; BMI, body mass index; eGFR, estimated Glomerular Filtration Rate; SBP, cholesterol, systolic blood pressure; DBP, diastolic blood pressure. | | | |

# Table S4a. Predictors of 2-year MACE models (risk factors selected by lasso regression)

| Predictor | OST OR (95%CI) | IFX OR (95%CI) | OBP  OR (95%CI) |
| --- | --- | --- | --- |
| Sex = Male (%) | 1.53 (1.3, 1.81) | 1.29 (1.14, 1.45) | 1.41 (1.28, 1.55) |
| SES (%) |  |  | x |
| 1 | ref | ref | x |
| 2 | 0.96 (0.8, 1.14) | 1 (0.88, 1.15) | x |
| 3 | 0.99 (0.83, 1.19) | 1.03 (0.9, 1.18) | x |
| 4 | 0.89 (0.73, 1.08) | 0.99 (0.86, 1.15) | x |
| 5 | 0.97 (0.78, 1.20) | 1.17 (1.01, 1.37) | x |
| Smoking** |  |  |  |
| Ex | ref | ref | ref |
| No | 1 (0.83, 1.20) | 0.98 (0.85, 1.13) | 0.94 (0.82, 1.09) |
| Yes | 1.25 (1, 1.56) | 1.2 (0.96, 1.5) | 1.15 (0.94, 1.4) |
| Drinking** |  |  |  |
| Ex | ref | ref | ref |
| No | 1.03 (0.73, 1.45) | 1.04 (0.8, 1.36) | 0.88 (0.67, 1.15) |
| Yes | 0.89 (0.6, 1.31) | 1.02 (0.77, 1.36) | 0.76 (0.58, 0.98) |
| Diabetes type I* | x | x | x |
| Diabetes type II* | x | x | 1.32 (1.14, 1.52) |
| Chronic obstructive pulmonary disease* | x | x | x |
| Chronic kidney disease* | x | x | x |
| Rheumatoid arthritis* | x | x | 0.86 (0.76, 0.97) |
| Lupus* | x | x | x |
| Systemic heart disease** | x | x | x |
| Anti-osteoporosis use** | x | x | x |
| Heparin use** | x | x | x |
| Beta-blocker use** | 1.04 (0.89, 1.21) | 1.18 (1.02, 1.35) | 1.15 (1.04, 1.26) |
| Hypertension** | x | x | x |
| Deep vein thrombosis or pulmonary embolism** | x | x | x |
| Anticoagulant use** | x | x | x |
| Antidepressants TCA** | x | x | x |
| Antidepressants SSRI** | 1.38 (1.15, 1.66) | x | x |
| Hypercholesterolemia** | x | x | x |
| Statin use** | x | 0.93 (0.74, 1.16) | x |
| Osteoporosis history* | x | 0.86 (0.72, 1.04) | 0.81 (0.74, 0.88) |
| Family history of cardiovascular disease | x | x | x |
| Family history of cardiovascular disease before age 60 | x | x | x |
| Heart failure* | x | x | x |
| Migraine* | x | x | x |
| Severe mental illness* | x | x | x |
| Vascular Disease* | x | x | x |
| Atrial fibrillation* | 1.15 (0.94, 1.4) | x | 1.35 (1.19, 1.53) |
| On anti-hypertensive drug | 1.3 (1.08, 1.55) | 1.18 (0.95, 1.47) | x |
| Antipsychotic use** | x | x | x |
| Steroid use** | x |  | x |
| Erectile dysfunction** | x | x | x |
| Age Group (%) |  |  |  |
| 50-59 | ref | ref | ref |
| 60-69 | 1.36 (0.94, 1.98) | 2.18 (1.4, 3.39) | 1.88 (1.45, 2.44) |
| 70-79 | 2.63 (1.85, 3.74) | 4.12 (2.59, 6.55) | 3.5 (2.73, 4.5) |
| 80-89 | 4.45 (3.1, 6.38) | 6.27 (3.57, 11.01) | 5.81 (4.49, 7.51) |
| >89 | 6.54 (4.36, 9.82) | 7.04 (3.59, 13.8) | 7.97 (6.02, 10.56) |
| Charlson score |  | x |  |
| 0 | ref | x | ref |
| 1 | 1.21 (1.03, 1.42) | x | 0.98 (0.88, 1.1) |
| 2 | 0.91 (0.75, 1.11) | x | 0.87 (0.77, 1) |
| ≥3 | 1.07 (0.87, 1.31) | x | 0.89 (0.77, 1.04) |
| Cardiovascular disease | x | x |  |
| No | x | x | ref |
| Ever >1 year before index date | x | x | 1.05 (0.94, 1.19) |
| 1 year before index | x | x | 1.11 (0.84, 1.45) |
| 6 months before index | x | x | 1.31 (1.03, 1.66) |
| 1 month before index | x | x | 1.86 (1.32, 2.62) |
| MI or Stroke |  |  |  |
| No | ref | ref | ref |
| Ever >1 year before index date | 1.16 (0.89, 1.51) | 1.52 (1.23, 1.86) | 1.3 (1.11, 1.52) |
| 1 year before index | 2.04 (1.49, 2.79) | 1.93 (1.53, 2.43) | 1.87 (1.55, 2.26) |
| Established CVD * | 1.65 (1.34, 2.04) | 1.37 (1.16, 1.62) | 1.68 (1.47, 1.92) |
| Any fracture history | x | x | x |
| No | x | x | x |
| Ever >1 year before index date | x | x | x |
| 1 year before index | x | x | x |
| Hip fracture history | x | x | x |
| No | x | x | x |
| Ever >1 year before index date | x | x | x |
| 1 year before index | x | x | x |
| Shoulder fracture history | x | x | x |
| No | x | x | x |
| Ever >1 year before index date | x | x | x |
| 1 year before index | x | x | x |
| Spine fracture history | x | x | x |
| No | x | x | x |
| Ever >1 year before index date | x | x | x |
| 1 year before index | x | x | x |
| Wrist fracture history | x | x | x |
| No | x | x | x |
| Ever >1 year before index date | x | x | x |
| 1 year before index | x | x | x |
| BMI** |  | x |  |
| <18.5 | ref | x | ref |
| 18.6 - 24.9 | 0.76 (0.56, 1.03) | x | 0.79 (0.66, 0.96) |
| 25 - 29.9 | 0.65 (0.44, 0.94) | x | 0.66 (0.51, 0.85) |
| 30 - 39.9 | 0.59 (0.36, 0.97) | x | 0.61 (0.45, 0.81) |
| >=40 | 0.76 (0.32, 1.79) | x | 0.62 (0.37, 1.04) |
| No. of GP visits** |  | x |  |
| 0 | ref | x | ref |
| 1-5 | 0.49 (0.35, 0.69) | x | 1 (0.86, 1.17) |
| 6-10 | 0.53 (0.37, 0.75) | x | 0.89 (0.74, 1.08) |
| 11-15 | 0.49 (0.34, 0.7) | x | 0.86 (0.71, 1.04) |
| >=16 | 0.55 (0.38, 0.78) | x | 0.93 (0.77, 1.13) |
| No. of GP emergency visits** |  |  | x |
| 0 | ref | ref | x |
| 1 | 1.19 (0.99, 1.43) | 1.25 (1.08, 1.44) | x |
| 2 | 1.32 (1.03, 1.7) | 1.21 (0.99, 1.48) | x |
| 3-5 | 1.17 (0.9, 1.52) | 1.24 (1.03, 1.5) | x |
| >=6 | 1.4 (0.98, 2.01) | 1.27 (0.97, 1.67) | x |
| eGFR** |  |  | x |
| <=29 | ref | ref | ref |
| 30 – 44 | 1.02 (0.57, 1.84) | 0.98 (0.67, 1.44) | 0.92 (0.66, 1.29) |
| 45 – 59 | 0.83 (0.47, 1.47) | 0.76 (0.37, 1.57) | 0.78 (0.51, 1.18) |
| 60 – 89 | 0.65 (0.37, 1.15) | 0.69 (0.16, 2.93) | 0.63 (0.39, 1.03) |
| >=90 | 0.67 (0.33, 1.36) | 0.62 (0.12, 3.3) | 0.61 (0.33, 1.12) |
| SBP** |  |  |  |
| <120 | ref | ref | ref |
| 120 - 139 | 1.04 (0.84, 1.29) | 1.08 (0.9, 1.3) | 1.03 (0.9, 1.17) |
| 140 - 159 | 1.25 (1.01, 1.55) | 1.18 (0.96, 1.46) | 1.14 (0.99, 1.31) |
| >=160 | 1.39 (1.08, 1.8) | 1.48 (1.06, 2.07) | 1.36 (1.14, 1.64) |
| DBP** | x |  | x |
| <80 | x | x | x |
| 80 - 89 | x | x | x |
| 90 - 99 | x | x | x |
| >=100 | x | x | x |
| No. of concomitant medicines** |  |  |  |
| 0 | ref | ref | ref |
| 1 – 3 | 0.72 (0.5, 1.03) | 0.81 (0.67, 0.98) | 0.77 (0.66, 0.91) |
| 4 – 6 | 0.8 (0.56, 1.15) | 0.99 (0.82, 1.18) | 0.87 (0.73, 1.03) |
| 7 – 9 | 1.05 (0.73, 1.52) | 0.97 (0.8, 1.18) | 1.03 (0.86, 1.23) |
| 10 – 12 | 1.1 (0.75, 1.6) | 1.02 (0.83, 1.27) | 1.2 (0.99, 1.45) |
| >=13 | 1.11 (0.75, 1.64) | 1.03 (0.82, 1.3) | 1.22 (1, 1.48) |
| Cholesterol measurement** (HDL/LDL) |  |  |  |
| <=3.5 | ref | ref | ref |
| 3.6 – 5 | 1.24 (0.95, 1.62) | 1.07 (0.55, 2.09) | 1.16 (1, 1.35) |
| >5 | 1.53 (1.07, 2.19) | 1.38 (0.27, 6.96) | 1.36 (1.03, 1.81) |
| No. of previous fractures* |  | x | x |
| 0 | ref | x | x |
| 1 | 1.06 (0.9, 1.25) | x | x |
| >=2 | 0.81 (0.68, 0.98) | x | x |
| Abbreviations: OST, patients with incident diagnosis of osteoporosis; IFX, patients with incident fragility fracture; OBP, incident users of oral bisphosphonates; OR, odds ratio; CI, confidence intervals; MACE, composite outcome for the occurrence of either myocardial infarction, stroke or cardiovascular disease death; * ever; ** in the year prior to start; SES, socio-economic status; MI, myocardial infarction; BMI, body mass index; eGFR, estimated Glomerular Filtration Rate; SBP, cholesterol, systolic blood pressure; DBP, diastolic blood pressure. | | | |

# Table S4b. Predictors of 1- and 2-year MI/Stroke models (risk factors selected by lasso regression)

| Predictor | OST OR (95%CI) | | IFX OR (95%CI) | | OBP  OR (95%CI) | |
| --- | --- | --- | --- | --- | --- | --- |
|  | One year | Two years | One year | Two years | One year | Two years |
| Sex = Male (%) | 1.79 (1.43, 2.23) | 1.4 (1.17, 1.68) | x | x | 1.39 (1.22, 1.58) | 1.38 (1.24, 1.53) |
| SES (%) | x |  | x |  | x |  |
| 1 | x | ref | x | ref | x | ref |
| 2 | x | 1.29 (1.06, 1.57) | x | 0.95 (0.8, 1.13) | x | 1.06 (0.94, 1.19) |
| 3 | x | 1.15 (0.94, 1.41) | x | 1 (0.83, 1.2) | x | 1.03 (0.91, 1.16) |
| 4 | x | 1.04 (0.84, 1.3) | x | 1.13 (0.94, 1.36) | x | 1.06 (0.93, 1.2) |
| 5 | x | 1.4 (1.11, 1.76) | x | 1.21 (0.98, 1.48) | x | 1.13 (0.98, 1.31) |
| Smoking** | x | x | x |  | x |  |
| Ex | x | x | x | ref | x | ref |
| No | x | x | x | 0.97 (0.78, 1.2) | x | 0.91 (0.76, 1.08) |
| Yes | x | x | x | 1.32 (0.83, 2.08) | x | 1.14 (0.92, 1.42) |
| Drinking** |  |  | x |  |  |  |
| Ex | ref | ref | x | ref | ref | ref |
| No | 1.07 (0.67, 1.7) | 1.06 (0.72, 1.55) | x | 1.05 (0.74, 1.49) | 1.18 (0.8, 1.74) | 0.95 (0.72, 1.26) |
| Yes | 0.83 (0.52, 1.31) | 0.91 (0.61, 1.37) | x | 1.07 (0.72, 1.59) | 0.96 (0.66, 1.39) | 0.86 (0.65, 1.13) |
| Diabetes type I* | x | x | x | x | x | x |
| Diabetes type II* | x | x | x | x | x | x |
| Chronic obstructive pulmonary disease* | x | x | x | x | x | x |
| Chronic kidney disease* | x | x | x | x | x | x |
| Rheumatoid arthritis* | x | x | x | x | x | x |
| Lupus* | x | x | x | x | x | x |
| Systemic heart disease** | x | x | x | x | x | x |
| Anti-osteoporosis use** | x | 1.02 (0.86, 1.21) | x | x | x | x |
| Heparin use** | x | x | x | x | x | x |
| Beta-blocker use** | x | 1.17 (0.99, 1.38) | 1.04 (0.85, 1.27) | 1.19 (0.98, 1.44) | 1.14 (0.99, 1.31) | 1.22 (1.1, 1.36) |
| Hypertension** | x | x | x | x | x | x |
| Deep vein thrombosis or pulmonary embolism** | x | x | x | x | x | x |
| Anticoagulant use** | x | x | x | x | x | x |
| Antidepressants TCA** | x | x | x | x | x | x |
| Antidepressants SSRI** | x | 1.27 (1.03, 1.57) | x | x | x | x |
| Hypercholesterolemia** | x | x | x | x | x | x |
| Statin use** | x | 0.97 (0.81, 1.15) | x | x | x | x |
| Osteoporosis history* | x | x | x | x | 0.8 (0.71, 0.91) | 0.81 (0.73, 0.89) |
| Family history of cardiovascular disease | x | x | x | x | x | x |
| Family history of cardiovascular disease before age 60 | x | x | x | x | x | x |
| Heart failure* | x | x | x | x | x | x |
| Migraine* | x | x | x | x | x | x |
| Severe mental illness* | x | x | x | x | x | x |
| Vascular Disease* | x | x | x | x | x | x |
| Atrial fibrillation* | x | 1.32 (1.07, 1.63) | 1.23 (0.98, 1.55) | 1.33 (1.09, 1.61) | 1.31 (1.1, 1.56) | x |
| On anti-hypertensive drug | 1.22 (0.94, 1.57) | 1.41 (1.16, 1.73) | 1.37 (0.97, 1.93) | 1.16 (0.81, 1.65) | 1.2 (1.02, 1.42) | 1.15 (1.02, 1.3) |
| Antipsychotic use** | x | x | x | x | x | x |
| Steroid use** | x |  | x | x | x | x |
| Erectile dysfunction** | x | x | x | x | x | x |
| Age Group (%) |  |  |  |  |  |  |
| 50-59 | ref | ref | ref | ref | ref | ref |
| 60-69 | 1.11 (0.65, 1.91) | 1.8 (1.17, 2.76) | 4.2 (1.94, 9.07) | 2.63 (1.49, 4.63) | 1.23 (0.87, 1.74) | 1.75 (1.34, 2.28) |
| 70-79 | 2.07 (1.26, 3.41) | 3.12 (2.06, 4.71) | 5.02 (2.19, 11.54) | 4.63 (2.24, 9.57) | 2.24 (1.62, 3.09) | 3.03 (2.35, 3.92) |
| 80-89 | 3.45 (2.09, 5.71) | 5.13 (3.37, 7.8) | 7.04 (2.69, 18.46) | 6.93 (2.65, 18.14) | 3.39 (2.43, 4.73) | 4.99 (3.83, 6.5) |
| >89 | 5.27 (3.03, 9.16) | 6.9 (4.34, 10.97) | 6.99 (2.34, 20.88) | 6.15 (1.87, 20.31) | 3.58 (2.47, 5.2) | 5.2 (3.87, 6.99) |
| Charlson score |  |  | x | x |  | x |
| 0 | ref | ref | x | x | ref | x |
| 1 | 1.26 (1, 1.59) | 1.24 (1.04, 1.47) | x | x | 1.01 (0.87, 1.17) | x |
| 2 | 0.86 (0.64, 1.15) | 0.95 (0.76, 1.18) | x | x | 0.99 (0.82, 1.19) | x |
| ≥3 | 0.96 (0.71, 1.29) | 1.14 (0.91, 1.44) | x | x | 1.04 (0.83, 1.31) | x |
| Cardiovascular disease | x | x | x | x |  |  |
| No | x | x | x | x | ref | ref |
| Ever >1 year before index date | x | x | x | x | 1.2 (1.01, 1.42) | 1.22 (1.07, 1.38) |
| 1 year before index | x | x | x | x | 1.29 (0.89, 1.85) | 1.09 (0.8, 1.48) |
| 6 months before index | x | x | x | x | 2.2 (1.65, 2.93) | 1.51 (1.16, 1.96) |
| 1 month before index | x | x | x | x | 2.62 (1.73, 3.98) | 2.15 (1.5, 3.08) |
| MI or Stroke | x | x | x |  |  |  |
| No | x | x | x | ref | ref | ref |
| Ever >1 year before index date | x | x | x | 1.16 (0.88, 1.53) | 1.21 (0.96, 1.53) | 1.3 (1.09, 1.56) |
| 1 year before index | x | x | x | 1.79 (1.32, 2.44) | 1.68 (1.29, 2.2) | 1.79 (1.45, 2.22) |
| Established CVD * | 2.19 (1.78, 2.7) | 2.14 (1.81, 2.53) | 1.8 (1.5, 2.17) | 1.41 (1.16, 1.72) | 1.36 (1.12, 1.66) | 1.37 (1.18, 1.59) |
| Any fracture history | x | x | x | x | x | x |
| No | x | x | x | x | x | x |
| Ever >1 year before index date | x | x | x | x | x | x |
| 1 year before index | x | x | x | x | x | x |
| Hip fracture history | x | x | x | x | x | x |
| No | x | x | x | x | x | x |
| Ever >1 year before index date | x | x | x | x | x | x |
| 1 year before index | x | x | x | x | x | x |
| Shoulder fracture history | x | x | x | x | x | x |
| No | x | x | x | x | x | x |
| Ever >1 year before index date | x | x | x | x | x | x |
| 1 year before index | x | x | x | x | x | x |
| Spine fracture history | x | x | x | x | x | x |
| No | x | x | x | x | x | x |
| Ever >1 year before index date | x | x | x | x | x | x |
| 1 year before index | x | x | x | x | x | x |
| Wrist fracture history | x | x | x | x | x | x |
| No | x | x | x | x | x | x |
| Ever >1 year before index date | x | x | x | x | x | x |
| 1 year before index | x | x | x | x | x | x |
| BMI** |  |  | x |  |  | x |
| <18.5 | ref | ref | x | ref | ref | x |
| 18.6 - 24.9 | 0.86 (0.55, 1.34) | 0.79 (0.57, 1.11) | x | 1.2 (0.42, 3.47) | 0.82 (0.62, 1.09) | x |
| 25 - 29.9 | 0.77 (0.44, 1.35) | 0.68 (0.46, 1) | x | 1.46 (0.25, 8.65) | 0.69 (0.49, 0.96) | x |
| 30 - 39.9 | 0.64 (0.33, 1.22) | 0.63 (0.37, 1.05) | x | 1.88 (0.17, 21.29) | 0.7 (0.47, 1.04) | x |
| >=40 | 0.77 (0.22, 2.66) | 0.8 (0.31, 2.11) | x | 2.77 (0.05, 153.82) | 0.65 (0.3, 1.41) | x |
| No. of GP visits** |  |  | x | x |  |  |
| 0 | ref | ref | x | x | ref | ref |
| 1-5 | 0.99 (0.55, 1.78) | 0.7 (0.48, 1.02) | x | x | 0.87 (0.68, 1.1) | 0.94 (0.78, 1.13) |
| 6-10 | 1.03 (0.56, 1.91) | 0.68 (0.46, 1.02) | x | x | 0.85 (0.64, 1.13) | 0.94 (0.75, 1.16) |
| 11-15 | 1.05 (0.56, 1.96) | 0.67 (0.45, 1.02) | x | x | 0.76 (0.56, 1.02) | 0.93 (0.75, 1.17) |
| >=16 | 1.28 (0.69, 2.38) | 0.82 (0.55, 1.23) | x | x | 0.82 (0.61, 1.1) | 1.05 (0.84, 1.31) |
| No. of GP emergency visits** | x | x | x | x |  |  |
| 0 | x | x | x | x | ref | ref |
| 1 | x | x | x | x | 1.17 (0.99, 1.38) | 1.11 (0.97, 1.26) |
| 2 | x | x | x | x | 1.67 (1.35, 2.07) | 1.35 (1.13, 1.61) |
| 3-5 | x | x | x | x | 1.14 (0.89, 1.46) | 1.25 (1.04, 1.5) |
| >=6 | x | x | x | x | 2.21 (1.65, 2.98) | 1.61 (1.26, 2.06) |
| eGFR** |  |  |  | x |  |  |
| <=29 | ref | ref | ref | ref | ref | ref |
| 30 – 44 | 0.79 (0.42, 1.49) | 0.96 (0.53, 1.74) | 0.77 (0.45, 1.31) | 0.93 (0.57, 1.51) | 1.04 (0.62, 1.76) | 0.96 (0.64, 1.44) |
| 45 – 59 | 0.64 (0.32, 1.27) | 0.74 (0.41, 1.35) | 0.6 (0.25, 1.41) | 0.73 (0.27, 1.94) | 0.91 (0.48, 1.7) | 0.81 (0.51, 1.3) |
| 60 – 89 | 0.44 (0.23, 0.86) | 0.57 (0.32, 1.02) | 0.53 (0.07, 4.23) | 0.66 (0.06, 7.82) | 0.65 (0.29, 1.5) | 0.65 (0.35, 1.21) |
| >=90 | 0.53 (0.2, 1.38) | 0.64 (0.28, 1.49) | 0.48 (0.06, 3.96) | 0.48 (0.03, 8.96) | 0.58 (0.22, 1.56) | 0.63 (0.31, 1.3) |
| SBP** |  |  |  |  |  |  |
| <120 | ref | ref | ref | ref | ref | ref |
| 120 - 139 | 0.94 (0.69, 1.27) | 0.87 (0.7, 1.1) | 0.95 (0.74, 1.22) | 1.1 (0.86, 1.41) | 1.01 (0.82, 1.25) | 1.08 (0.93, 1.25) |
| 140 - 159 | 1.16 (0.86, 1.58) | 0.96 (0.76, 1.22) | 1.1 (0.82, 1.48) | 1.2 (0.89, 1.63) | 1.18 (0.94, 1.48) | 1.19 (1.02, 1.39) |
| >=160 | 1.39 (0.97, 2) | 1.21 (0.91, 1.62) | 1.34 (0.85, 2.11) | 1.72 (1.05, 2.83) | 1.51 (1.14, 2.01) | 1.39 (1.15, 1.68) |
| DBP** | x |  | x | x |  | x |
| <80 | x | ref | x | x | ref | x |
| 80 - 89 | x | 1.07 (0.9, 1.27) | x | x | 1.02 (0.88, 1.18) | x |
| 90 - 99 | x | 1.07 (0.81, 1.42) | x | x | 1.03 (0.82, 1.3) | x |
| >=100 | x | 1.11 (0.66, 1.88) | x | x | 1.19 (0.76, 1.85) | x |
| No. of concomitant medicines** |  |  | x |  |  |  |
| 0 | ref | ref | x | ref | ref | ref |
| 1 – 3 | 0.96 (0.55, 1.69) | 0.93 (0.63, 1.37) | x | 0.93 (0.73, 1.18) | 0.82 (0.64, 1.07) | 0.79 (0.66, 0.96) |
| 4 – 6 | 0.76 (0.42, 1.35) | 0.8 (0.54, 1.19) | x | 0.9 (0.71, 1.12) | 0.8 (0.6, 1.05) | 0.79 (0.64, 0.97) |
| 7 – 9 | 1.04 (0.58, 1.86) | 0.86 (0.57, 1.3) | x | 0.78 (0.61, 1.01) | 1.02 (0.76, 1.36) | 0.86 (0.69, 1.07) |
| 10 – 12 | 0.96 (0.52, 1.75) | 0.83 (0.54, 1.27) | x | 0.91 (0.69, 1.21) | 1.08 (0.79, 1.47) | 0.96 (0.76, 1.21) |
| >=13 | 1.35 (0.74, 2.47) | 0.88 (0.56, 1.36) | x | 1 (0.71, 1.41) | 1.09 (0.79, 1.49) | 0.9 (0.71, 1.15) |
| Cholesterol measurement** (HDL/LDL) | x |  | x |  | x |  |
| <=3.5 | x | ref | ref | ref | x | ref |
| 3.6 – 5 | x | 1.28 (0.95, 1.73) | 1 (0.31, 3.21) | 1.03 (0.29, 3.63) | x | 1.13 (0.96, 1.32) |
| >5 | x | 1.49 (1.04, 2.14) | 1.75 (0.23, 13.25) | 1.5 (0.14, 15.72) | x | 1.36 (1.01, 1.82) |
| No. of previous fractures* | x |  | x | x | x | x |
| 0 | x | ref | x | x | x | x |
| 1 | x | 1.15 (0.96, 1.36) | x | x | x | x |
| >=2 | x | 1.05 (0.87, 1.26) | x | x | x | x |
| Abbreviations: OST, patients with incident diagnosis of osteoporosis; IFX, patients with incident fragility fracture; OBP, incident users of oral bisphosphonates; OR, odds ratio; CI, confidence intervals; MI, myocardial infarction; * ever; ** in the year prior to start; SES, socio-economic status; BMI, body mass index; eGFR, estimated Glomerular Filtration Rate; SBP, cholesterol, systolic blood pressure; DBP, diastolic blood pressure. | | | | | | |

# Table S5a. Model equations for 2-year MACE models (risk factors selected by lasso regression)

| Predictor | OST Beta coefficients | IFX Beta coefficients | OBP Beta coefficients |
| --- | --- | --- | --- |
| Intercept | -3.711 | -4.661 | -3.863 |
| Sex = Male (%) | 0.425 | 0.253 | 0.342 |
| SES (%) |  |  | x |
| 1 | ref | ref | x |
| 2 | -0.043 | 0.004 | x |
| 3 | -0.010 | 0.028 | x |
| 4 | -0.119 | -0.006 | x |
| 5 | -0.030 | 0.161 | x |
| Smoking** |  | x |  |
| Ex | ref | ref | ref |
| No | -0.001 | -0.021 | -0.058 |
| Yes | 0.223 | 0.182 | 0.139 |
| Drinking** |  |  |  |
| Ex | ref | ref | ref |
| No | 0.031 | 0.043 | -0.131 |
| Yes | -0.119 | 0.022 | -0.280 |
| Diabetes type I* | x | x | x |
| Diabetes type II* |  | x | 0.274 |
| Chronic obstructive pulmonary disease* | x | x | x |
| Chronic kidney disease* | x | x | x |
| Rheumatoid arthritis* | x | x | -0.152 |
| Lupus* | x | x | x |
| Systemic heart disease** | x | x | x |
| Anti-osteoporosis use** | x | x | x |
| Heparin use** | x | x | x |
| Beta-blocker use** | 0.039 | 0.163 | 0.137 |
| Hypertension** | x | x | x |
| Deep vein thrombosis or pulmonary embolism** | x | x | x |
| Anticoagulant use** | x | x | x |
| Antidepressants TCA** | x | x | x |
| Antidepressants SSRI** | 0.323 | x | x |
| Hypercholesterolemia** | x | x | x |
| Statin use** | x | -0.077 | x |
| Osteoporosis history* | x | -0.148 | -0.214 |
| Family history of cardiovascular disease (%) | x | x | x |
| Family history of cardiovascular disease before age 60 | x | x | x |
| Heart failure* | x | x | x |
| Migraine* | x | x | x |
| Severe mental illness* | x | x | x |
| Vascular Disease* | x | x | x |
| Atrial fibrillation* | 0.141 | x | 0.299 |
| On anti-hypertensive drug | 0.259 | 0.164 | x |
| Antipsychotic use** | x | x | x |
| Steroid use** | x |  | x |
| Erectile dysfunction** | x | x | x |
| Age Group (%) |  |  |  |
| 50-59 | ref | ref | ref |
| 60-69 | 0.309 | 0.780 | 0.630 |
| 70-79 | 0.968 | 1.416 | 1.254 |
| 80-89 | 1.492 | 1.835 | 1.759 |
| >89 | 1.878 | 1.952 | 2.076 |
| Charlson score |  | x |  |
| 0 | ref | x | ref |
| 1 | 0.190 | x | -0.016 |
| 2 | -0.092 | x | -0.134 |
| ≥3 | 0.064 | x | -0.115 |
| Cardiovascular disease | x | x |  |
| No | x | x | ref |
| Ever >1 year before index date | x | x | 0.053 |
| 1 year before index | x | x | 0.101 |
| 6 months before index | x | x | 0.267 |
| 1 month before index | x | x | 0.618 |
| MI or Stroke |  |  |  |
| No | ref | ref | ref |
| Ever >1 year before index date | 0.148 | 0.416 | 0.263 |
| 1 year before index | 0.711 | 0.655 | 0.628 |
| Established CVD * | 0.504 | 0.317 | 0.517 |
| Any fracture history | x | x | x |
| No | x | x | x |
| Ever >1 year before index date | x | x | x |
| 1 year before index | x | x | x |
| Hip fracture history | x | x | x |
| No | x | x | x |
| Ever >1 year before index date | x | x | x |
| 1 year before index | x | x | x |
| Shoulder fracture history | x | x | x |
| No | x | x | x |
| Ever >1 year before index date | x | x | x |
| 1 year before index | x | x | x |
| Spine fracture history | x | x | x |
| No | x | x | x |
| Ever >1 year before index date | x | x | x |
| 1 year before index | x | x | x |
| Wrist fracture history | x | x | x |
| No | x | x | x |
| Ever >1 year before index date | x | x | x |
| 1 year before index | x | x | x |
| BMI** |  | x |  |
| <18.5 | ref | x | ref |
| 18.6 - 24.9 | -0.276 | x | -0.233 |
| 25 - 29.9 | -0.438 | x | -0.420 |
| 30 - 39.9 | -0.531 | x | -0.501 |
| >=40 | -0.273 | x | -0.472 |
| No. of GP visits** |  | x |  |
| 0 | ref | x | ref |
| 1-5 | -0.709 | x | -0.000 |
| 6-10 | -0.636 | x | -0.112 |
| 11-15 | -0.721 | x | -0.153 |
| >=16 | -0.606 | x | -0.072 |
| No. of GP emergency visits** |  |  | x |
| 0 | ref | ref | x |
| 1 | 0.173 | 0.222 | x |
| 2 | 0.278 | 0.188 | x |
| 3-5 | 0.158 | 0.219 | x |
| >=6 | 0.338 | 0.240 | x |
| eGFR** |  |  |  |
| <=29 | ref | ref | ref |
| 30 – 44 | 0.020 | -0.018 | -0.082 |
| 45 – 59 | -0.186 | -0.278 | -0.249 |
| 60 – 89 | -0.427 | -0.369 | -0.459 |
| >=90 | -0.403 | -0.477 | -0.494 |
| SBP** |  |  |  |
| <120 | ref | ref | ref |
| 120 - 139 | 0.044 | 0.077 | 0.025 |
| 140 - 159 | 0.221 | 0.165 | 0.127 |
| >=160 | 0.330 | 0.394 | 0.311 |
| DBP** | x | x | x |
| <80 | x | x | x |
| 80 - 89 | x | x | x |
| 90 - 99 | x | x | x |
| >=100 | x | x | x |
| No. of concomitant medicines** |  |  |  |
| 0 | ref | ref | ref |
| 1 – 3 | -0.334 | -0.212 | -0.255 |
| 4 – 6 | -0.219 | -0.015 | -0.143 |
| 7 – 9 | 0.050 | -0.030 | 0.031 |
| 10 – 12 | 0.091 | 0.025 | 0.180 |
| >=13 | 0.102 | 0.031 | 0.195 |
| Cholesterol measurement** (HDL/LDL) |  |  |  |
| <=3.5 | ref | ref | ref |
| 3.6 – 5 | 0.215 | 0.067 | 0.148 |
| >5 | 0.423 | 0.322 | 0.310 |
| No. of previous fractures* |  | x | x |
| 0 | ref | x | x |
| 1 | 0.060 | x | x |
| >=2 | -0.205 | x | x |
| Abbreviations: OST, patients with incident diagnosis of osteoporosis; IFX, patients with incident fragility fracture; OBP, incident users of oral bisphosphonates; OR, odds ratio; CI, confidence intervals; MACE, composite outcome for the occurrence of either myocardial infarction, stroke or cardiovascular disease death; * ever; ** in the year prior to start; SES, socio-economic status; MI, myocardial infarction; BMI, body mass index; eGFR, estimated Glomerular Filtration Rate; SBP, cholesterol, systolic blood pressure; DBP, diastolic blood pressure. | | | |

# Table S5b. Model equations for 1- and 2-year MI/Stroke models (risk factors selected by lasso regression)

| Predictor | OST Beta coefficients | | IFX Beta coefficients | | OBP  Beta coefficients | |
| --- | --- | --- | --- | --- | --- | --- |
|  | One year | Two years | One year | Two years | One year | Two years |
| Intercept | -4.635 | -4.341 | -5.493 | -5.549 | -4.580 | -4.456 |
| Sex = Male (%) | 0.582 | 0.337 | x | x | 0.327 | 0.321 |
| SES (%) | x |  | x |  | x |  |
| 1 | x | ref | x | ref | x | ref |
| 2 | x | 0.255 | x | -0.048 | x | 0.056 |
| 3 | x | 0.140 | x | -0.001 | x | 0.026 |
| 4 | x | 0.042 | x | 0.126 | x | 0.057 |
| 5 | x | 0.337 | x | 0.187 | x | 0.126 |
| Smoking** | x | x | x |  | x |  |
| Ex | x | x | x | ref | x | ref |
| No | x | x | x | -0.032 | x | -0.098 |
| Yes | x | x | x | 0.274 | x | 0.135 |
| Drinking** |  |  | x |  |  |  |
| Ex | ref | ref | x | ref | ref | ref |
| No | 0.065 | 0.055 | x | 0.053 | 0.168 | -0.049 |
| Yes | -0.192 | -0.091 | x | 0.065 | -0.045 | -0.151 |
| Diabetes type I* | x | x | x | x | x | x |
| Diabetes type II* | x | x | x | x | x | x |
| Chronic obstructive pulmonary disease* | x | x | x | x | x | x |
| Chronic kidney disease* | x | x | x | x | x | x |
| Rheumatoid arthritis* | x | x | x | x | x | x |
| Lupus* | x | x | x | x | x | x |
| Systemic heart disease** | x | x | x | x | x | x |
| Anti-osteoporosis use** | x | 0.019 | x | x | x | x |
| Heparin use** | x | x | x | x | x | x |
| Beta-blocker use** | x | 0.155 | 0.039 | 0.174 | 0.127 | 0.203 |
| Hypertension** | x | x | x | x | x | x |
| Deep vein thrombosis or pulmonary embolism** | x | x | x | x | x | x |
| Anticoagulant use** | x | x | x | x | x | x |
| Antidepressants TCA** | x | x | x | x | x | x |
| Antidepressants SSRI** | x | 0.240 | x | x | x | x |
| Hypercholesterolemia** | x | x | x | x | x | x |
| Statin use** | x | -0.035 | x | x | x | x |
| Osteoporosis history* | x | x | x | x | -0.221 | -0.212 |
| Family history of cardiovascular disease (%) | x | x | x | x | x | x |
| Family history of cardiovascular disease before age 60 | x | x | x | x | x | x |
| Heart failure* | x | x | x | x | x | x |
| Migraine* | x | x | x | x | x | x |
| Severe mental illness* | x | x | x | x | x | x |
| Vascular Disease* | x | x | x | x | x | x |
| Atrial fibrillation* | x | 0.279 | 0.211 | 0.283 | 0.270 | x |
| On anti-hypertensive drug | 0.196 | 0.347 | 0.312 | 0.145 | 0.185 | 0.140 |
| Antipsychotic use** | x | x | x | x | x | x |
| Steroid use** | x |  | x | x | x | x |
| Erectile dysfunction** | x | x | x | x | x | x |
| Age Group (%) |  |  |  |  |  |  |
| 50-59 | ref | ref | ref | ref | ref | ref |
| 60-69 | 0.108 | 0.586 | 1.434 | 0.966 | 0.208 | 0.560 |
| 70-79 | 0.730 | 1.136 | 1.614 | 1.532 | 0.805 | 1.109 |
| 80-89 | 1.239 | 1.634 | 1.952 | 1.936 | 1.222 | 1.608 |
| >89 | 1.662 | 1.932 | 1.945 | 1.817 | 1.277 | 1.648 |
| Charlson score |  |  | x | x |  | x |
| 0 | ref | ref | x | x | ref | x |
| 1 | 0.233 | 0.212 | x | x | 0.009 | x |
| 2 | -0.150 | -0.056 | x | x | -0.010 | x |
| ≥3 | -0.042 | 0.134 | x | x | 0.040 | x |
| Cardiovascular disease | x | x | x | x |  |  |
| No | x | x | x | x | ref | ref |
| Ever >1 year before index date | x | x | x | x | 0.181 | 0.197 |
| 1 year before index | x | x | x | x | 0.252 | 0.083 |
| 6 months before index | x | x | x | x | 0.789 | 0.414 |
| 1 month before index | x | x | x | x | 0.964 | 0.767 |
| MI or Stroke | x | x | x |  |  |  |
| No | x | x | x | ref | ref | ref |
| Ever >1 year before index date | x | x | x | 0.146 | 0.190 | 0.264 |
| 1 year before index | x | x | x | 0.585 | 0.519 | 0.583 |
| Established CVD * | 0.785 | 0.760 | 0.591 | 0.345 | 0.310 | 0.312 |
| Any fracture history | x | x | x | x | x | x |
| No | x | x | x | x | x | x |
| Ever >1 year before index date | x | x | x | x | x | x |
| 1 year before index | x | x | x | x | x | x |
| Hip fracture history | x | x | x | x | x | x |
| No | x | x | x | x | x | x |
| Ever >1 year before index date | x | x | x | x | x | x |
| 1 year before index | x | x | x | x | x | x |
| Shoulder fracture history | x | x | x | x | x | x |
| No | x | x | x | x | x | x |
| Ever >1 year before index date | x | x | x | x | x | x |
| 1 year before index | x | x | x | x | x | x |
| Spine fracture history | x | x | x | x | x | x |
| No | x | x | x | x | x | x |
| Ever >1 year before index date | x | x | x | x | x | x |
| 1 year before index | x | x | x | x | x | x |
| Wrist fracture history | x | x | x | x | x | x |
| No | x | x | x | x | x | x |
| Ever >1 year before index date | x | x | x | x | x | x |
| 1 year before index | x | x | x | x | x | x |
| BMI** |  |  | x | x |  | x |
| <18.5 | ref | ref | x | x | ref | x |
| 18.6 - 24.9 | -0.149 | -0.232 | x | 0.184 | -0.201 | x |
| 25 - 29.9 | -0.261 | -0.391 | x | 0.376 | -0.371 | x |
| 30 - 39.9 | -0.453 | -0.469 | x | 0.633 | -0.361 | x |
| >=40 | -0.265 | -0.218 | x | 1.0188 | -0.434 | x |
| No. of GP visits** |  |  | x | x |  |  |
| 0 | ref | ref | x | x | ref | ref |
| 1-5 | -0.009 | -0.356 | x | x | -0.142 | -0.061 |
| 6-10 | 0.032 | -0.380 | x | x | -0.164 | -0.065 |
| 11-15 | 0.045 | -0.394 | x | x | -0.279 | -0.068 |
| >=16 | 0.246 | -0.200 | x | x | -0.197 | 0.051 |
| No. of GP emergency visits** | x | x | x | x |  |  |
| 0 | x | x | x | x | ref | ref |
| 1 | x | x | x | x | 0.156 | 0.100 |
| 2 | x | x | x | x | 0.513 | 0.299 |
| 3-5 | x | x | x | x | 0.129 | 0.222 |
| >=6 | x | x | x | x | 0.795 | 0.476 |
| eGFR** |  |  |  |  |  |  |
| <=29 | ref | ref | ref | ref | ref | ref |
| 30 – 44 | -0.237 | -0.039 | -0.263 | -0.073 | 0.040 | -0.037 |
| 45 – 59 | -0.448 | -0.301 | -0.518 | -0.321 | -0.098 | -0.208 |
| 60 – 89 | -0.812 | -0.565 | -0.633 | -0.416 | -0.424 | -0.426 |
| >=90 | -0.642 | -0.442 | -0.738 | -0.727 | -0.540 | -0.458 |
| SBP** |  |  |  |  |  |  |
| <120 | ref | ref | ref | ref | ref | ref |
| 120 - 139 | -0.062 | -0.136 | -0.056 | 0.097 | 0.014 | 0.077 |
| 140 - 159 | 0.151 | -0.039 | 0.096 | 0.185 | 0.164 | 0.175 |
| >=160 | 0.331 | 0.193 | 0.289 | 0.543 | 0.413 | 0.329 |
| DBP** | x |  | x | x |  | x |
| <80 | x | ref | x | x | ref | x |
| 80 - 89 | x | 0.063 | x | x | 0.018 | x |
| 90 - 99 | x | 0.071 | x | x | 0.034 | x |
| >=100 | x | 0.108 | x | x | 0.171 | x |
| No. of concomitant medicines** |  |  | x |  |  |  |
| 0 | ref | ref | x | ref | ref | ref |
| 1 – 3 | -0.040 | -0.076 | x | -0.072 | -0.193 | -0.231 |
| 4 – 6 | -0.279 | -0.223 | x | -0.111 | -0.229 | -0.237 |
| 7 – 9 | 0.038 | -0.153 | x | -0.243 | 0.015 | -0.151 |
| 10 – 12 | -0.045 | -0.191 | x | -0.091 | 0.077 | -0.044 |
| >=13 | 0.298 | -0.133 | x | 0.004 | 0.085 | -0.104 |
| Cholesterol measurement** (HDL/LDL) | x |  | x |  | x |  |
| <=3.5 | x | ref | ref | ref | x | ref |
| 3.6 – 5 | x | 0.249 | -0.004 | 0.027 | x | 0.120 |
| >5 | x | 0.399 | 0.557 | 0.405 | x | 0.306 |
| No. of previous fractures* | x |  | x | x |  | x |
| 0 | x | ref | x | x | ref | x |
| 1 | x | 0.136 | x | x |  | x |
| >=2 | x | 0.047 | x | x |  | x |
| Abbreviations: OST, patients with incident diagnosis of osteoporosis; IFX, patients with incident fragility fracture; OBP, incident users of oral bisphosphonates; MI, myocardial infarction; * ever; ** in the year prior to start; SES, socio-economic status; BMI, body mass index; eGFR, estimated Glomerular Filtration Rate; SBP, cholesterol, systolic blood pressure; DBP, diastolic blood pressure. | | | | | | |

# Table S6a. Risk factors selected by lasso for one- and two-year models in gender based models (OST cohort)

| OST cohort  Predictor | Women OR (95%CI) | | | | Men OR (95%CI) | | | |
| --- | --- | --- | --- | --- | --- | --- | --- | --- |
|  | One year | | Two year | | One year | | Two year | |
|  | MACE | Stroke/MI | MACE | Stroke/MI | MACE | Stroke/MI | MACE | Stroke/MI |
| SES |  | x |  | x | x | x | x | x |
| 1 | ref | x | ref | x | x | x | x | x |
| 2 | 0.92 (0.7, 1.21) | x | 1.01 (0.83, 1.24) | x | x | x | x | x |
| 3 | 1 (0.76, 1.32) | x | 1.15 (0.94, 1.42) | x | x | x | x | x |
| 4 | 1.03 (0.78, 1.37) | x | 0.99 (0.8, 1.24) | x | x | x | x | x |
| 5 | 0.96 (0.69, 1.32) | x | 1.35 (1.07, 1.69) | x | x | x | x | x |
| Smoking** |  | x |  | x | x | x | x | x |
| Ex | ref | x | ref | x | x | x | x | x |
| No | 1.01 (0.77, 1.32) | x | 1.03 (0.83, 1.29) | x | x | x | x | x |
| Yes | 1.51 (1.06, 2.16) | x | 1.32 (0.99, 1.76) | x | x | x | x | x |
| Drinking** |  |  |  |  | x | x | x | x |
| Ex | ref | ref | ref | ref | x | x | x | x |
| No | 1.03 (0.61, 1.75) | 1.02 (0.65, 1.61) | 0.97 (0.64, 1.45) | 0.99 (0.66, 1.46) | x | x | x | x |
| Yes | 0.8 (0.47, 1.35) | 0.75 (0.45, 1.27) | 0.83 (0.54, 1.26) | 0.85 (0.55, 1.3) | x | x | x | x |
| Diabetes type I* | x | x | x | x | x | x | x | x |
| Diabetes type II* | x | x | x |  | x | x | x | x |
| Chronic obstructive pulmonary disease* | x | x | x | x | x | x | x | x |
| Chronic kidney disease* | x | x | 0.88 (0.66, 1.18) | x | x | x | x | x |
| Rheumatoid arthritis* | x | x | x | x | x | x | x | x |
| Lupus* | x | x | x | x | x | x | x | x |
| Systemic heart disease** | x | x | x | x | x | x | x | x |
| Anti-osteoporosis use** |  | x | 1.12 (0.94, 1.32) | x | x | x | x | x |
| Heparin use** | x | x | x | x | x | x | x | x |
| Beta-blocker use** | x | 1.48 (1.17, 1.87) | x | 1.23 (1.03, 1.47) | x | x | x | x |
| Hypertension** | x | x | x | x | x | x | x | x |
| Deep vein thrombosis or pulmonary embolism** | x | x | x | x | x | x | x | x |
| Anticoagulant use** | x | x | x | x | x | x | x | x |
| Antidepressants TCA** | x | x | x | x | x | x | x | x |
| Antidepressants SSRI** | 1.42 (1.09, 1.86) | x | 1.27 (1.03, 1.57) | x | x | x | x | x |
| Hypercholesterolemia** | x | x | x | x | x | x | x | x |
| Statin use** | x | x | x | x | x | x | x | x |
| Osteoporosis history* | x | x | x | x | x | x | x | x |
| Family history of cardiovascular disease (%) | x | x | x | x | x | x | x | x |
| Family history of cardiovascular disease before age 60 | x | x | x | x | x | x | x | x |
| Heart failure* | x | x | x | x | x | x | x | x |
| Migraine* | x | x | x |  | x | x | x | x |
| Severe mental illness* | x | x | x | x | x | x | x | x |
| Vascular Disease* | x | x | x | x | x | x | x | x |
| Atrial fibrillation* | 1.37 (1.03, 1.81) | x | 1.35 (1.08, 1.69) | x | x | x | x | x |
| On anti-hypertensive drug | 1.27 (0.97, 1.66) | 1.21 (0.89, 1.62) | 1.17 (0.97, 1.42) | 1.51 (1.21, 1.89) | x | x |  | x |
| Antipsychotic use** | x | x | x | x | x | x | x | x |
| Steroid use** | 1.05 (0.81, 1.38) | x | 1.14 (0.94, 1.39) |  | x | x | x | x |
| Erectile dysfunction** | x | x | x | x | x | x | x | x |
| Age Group |  |  |  |  |  | x |  | x |
| 50-59 | ref | ref | ref | ref | ref | x | ref | x |
| 60-69 | 1.03 (0.56, 1.89) | 1.04 (0.52, 2.06) | 2.23 (1.35, 3.68) | 1.63 (0.98, 2.72) | 1.73 (0.81, 3.71) | x | 2.98 (1.39, 6.41) | x |
| 70-79 | 2.57 (1.49, 4.42) | 2.87 (1.57, 5.27) | 4.57 (2.82, 7.4) | 3.5 (2.17, 5.64) | 1.52 (0.73, 3.2) | x | 3.46 (1.64, 7.3) | x |
| 80-89 | 4.66 (2.67, 8.15) | 5.21 (2.84, 9.56) | 7.87 (4.81, 12.86) | 5.25 (3.24, 8.52) | 2.2 (1.06, 4.56) | x | 4.66 (2.2, 9.88) | x |
| >89 | 5.83 (3.13, 10.83) | 6.73 (3.48, 13.01) | 11.44 (6.74, 19.42) | 7.45 (4.42, 12.56) | 2.11 (0.8, 5.6) | x | 5.12 (2.1, 12.48) | x |
| Charlson score |  | x |  |  | x | x | x | x |
| 0 | ref | x | ref | ref | x | x | x | x |
| 1 | 1.28 (1, 1.62) | x | 1.25 (1.05, 1.49) | 1.26 (1.05, 1.53) | x | x | x | x |
| 2 | 0.93 (0.69, 1.26) | x | 1.03 (0.81, 1.3) | 1 (0.79, 1.27) | x | x | x | x |
| ≥3 | 1.3 (0.96, 1.76) | x | 1.22 (0.92, 1.61) | 1.21 (0.95, 1.54) | x | x | x | x |
| Cardiovascular disease | x | x | x | x | x | x | x | x |
| No | x | x | x | x | x | x | x | x |
| Ever >1 year before index date | x | x | x | x | x | x | x | x |
| 1 year before index | x | x | x | x | x | x | x | x |
| 6 months before index | x | x | x | x | x | x | x | x |
| 1 month before index | x | x | x | x | x | x | x | x |
| MI or Stroke |  | x |  | x | x | x | x | x |
| No | ref | x | ref | x | x | x | x | x |
| Ever >1 year before index date | 1.33 (0.89, 1.99) | x | 1.3 (0.96, 1.77) | x | x | x | x | x |
| 1 year before index | 2.45 (1.55, 3.86) | x | 1.68 (1.15, 2.47) | x | x | x | x | x |
| Established CVD * | 1.53 (1.11, 2.11) | 2.21 (1.75, 2.78) | 1.6 (1.26, 2.04) | 2.01 (1.68, 2.4) | 2.31 (1.58, 3.39) | 2.19 (1.42, 3.36) | 2.24 (1.67, 3.01) | 2.29 (1.64, 3.19) |
| Any fracture history |  | x | x | x | x | x | x | x |
| No | ref | x | x | x | x | x | x | x |
| Ever >1 year before index date | 1 (0.64, 1.55) | x | x | x | x | x | x | x |
| 1 year before index | 1.16 (0.78, 1.73) | x | x | x | x | x | x | x |
| Hip fracture history | x | x | x | x | x | x | x | x |
| No | x | x | x | x | x | x | x | x |
| Ever >1 year before index date | x | x | x | x | x | x | x | x |
| 1 year before index | x | x | x | x | x | x | x | x |
| Shoulder fracture history | x | x | x | x | x | x | x | x |
| No | x | x | x | x | x | x | x | x |
| Ever >1 year before index date | x | x | x | x | x | x | x | x |
| 1 year before index | x | x | x | x | x | x | x | x |
| Spine fracture history | x | x | x | x | x | x | x | x |
| No | x | x | x | x | x | x | x | x |
| Ever >1 year before index date | x | x | x | x | x | x | x | x |
| 1 year before index | x | x | x | x | x | x | x | x |
| Wrist fracture history | x | x | x | x | x | x | x | x |
| No | x | x | x | x | x | x | x | x |
| Ever >1 year before index date | x | x | x | x | x | x | x | x |
| 1 year before index | x | x | x | x | x | x | x | x |
| BMI** |  | x |  |  | x | x | x | x |
| <18.5 | ref | x | ref | ref | x | x | x | x |
| 18.6 - 24.9 | 0.65 (0.46, 0.91) | x | 0.71 (0.53, 0.95) | 0.83 (0.55, 1.25) | x | x | x | x |
| 25 - 29.9 | 0.52 (0.32, 0.85) | x | 0.6 (0.42, 0.86) | 0.71 (0.45, 1.13) | x | x | x | x |
| 30 - 39.9 | 0.42 (0.23, 0.76) | x | 0.51 (0.29, 0.9) | 0.7 (0.37, 1.35) | x | x | x | x |
| >=40 | 0.52 (0.14, 1.94) | x | 0.9 (0.37, 2.16) | 0.95 (0.37, 2.45) | x | x | x | x |
| No. of GP visits** |  |  | x |  | x | x | x | x |
| 0 | ref | ref | x | ref | x | x | x | x |
| 1-5 | 0.73 (0.44, 1.22) | 0.88 (0.49, 1.56) | x | 0.66 (0.43, 1) | x | x | x | x |
| 6-10 | 0.57 (0.33, 1) | 0.76 (0.41, 1.41) | x | 0.63 (0.4, 0.97) | x | x | x | x |
| 11-15 | 0.6 (0.34, 1.06) | 0.78 (0.41, 1.46) | x | 0.65 (0.41, 1.02) | x | x | x | x |
| >=16 | 0.64 (0.36, 1.13) | 0.9 (0.48, 1.68) | x | 0.73 (0.47, 1.14) | x | x | x | x |
| No. of GP emergency visits** |  | x |  | x | x | x | x | x |
| 0 | ref | x | ref | x | x | x | x | x |
| 1 | 1.59 (1.23, 2.06) | x | 1.01 (0.81, 1.25) | x | x | x | x | x |
| 2 | 1.67 (1.17, 2.37) | x | 1.15 (0.86, 1.53) | x | x | x | x | x |
| 3-5 | 1.46 (1.01, 2.1) | x | 1.37 (1.04, 1.8) | x | x | x | x | x |
| >=6 | 2.09 (1.31, 3.34) | x | 1.91 (1.34, 2.71) | x | x | x | x | x |
| eGFR** |  |  |  |  | x | x |  |  |
| <=29 | ref | ref | ref | ref | x | x | ref | ref |
| 30 – 44 | 0.6 (0.3, 1.22) | 0.71 (0.33, 1.55) | 0.98 (0.54, 1.76) | 0.72 (0.4, 1.29) | x | x | 1.96 (0.26, 14.93) | 1.47 (0.19, 11.23) |
| 45 – 59 | 0.56 (0.29, 1.1) | 0.59 (0.29, 1.19) | 0.77 (0.43, 1.38) | 0.61 (0.35, 1.08) | x | x | 1.44 (0.21, 9.73) | 0.96 (0.14, 6.55) |
| 60 – 89 | 0.44 (0.23, 0.84) | 0.46 (0.23, 0.95) | 0.62 (0.34, 1.15) | 0.49 (0.28, 0.84) | x | x | 1.17 (0.19, 7.38) | 0.74 (0.11, 4.75) |
| >=90 | 0.47 (0.17, 1.28) | 0.55 (0.2, 1.56) | 0.67 (0.29, 1.56) | 0.52 (0.23, 1.16) | x | x | 1.19 (0.17, 8.26) | 0.59 (0.08, 4.5) |
| SBP** |  | x |  | x | x | x | x | x |
| <120 | ref | x | ref | x | x | x | x | x |
| 120 - 139 | 1.09 (0.78, 1.52) | x | 0.96 (0.75, 1.22) | x | x | x | x | x |
| 140 - 159 | 1.21 (0.86, 1.7) | x | 1.21 (0.94, 1.57) | x | x | x | x | x |
| >=160 | 1.3 (0.87, 1.94) | x | 1.32 (0.97, 1.81) | x | x | x | x | x |
| DBP** | x | x |  | x | x | x | x | x |
| <80 | x | x | ref | x | x | x | x | x |
| 80 – 89 | x | x | 1.08 (0.9, 1.29) | x | x | x | x | x |
| 90 – 99 | x | x | 1.02 (0.76, 1.36) | x | x | x | x | x |
| >=100 | x | x | 1.26 (0.76, 2.07) | x | x | x | x | x |
| No. of concomitant medicines** |  |  |  |  | x |  | x | x |
| 0 | ref | ref | ref | ref | x | ref | x | x |
| 1 – 3 | 0.54 (0.32, 0.94) | 0.72 (0.4, 1.28) | 0.64 (0.46, 0.89) | 0.84 (0.54, 1.31) | x | 0.35 (0.09, 1.42) | x | x |
| 4 – 6 | 0.72 (0.43, 1.22) | 0.68 (0.38, 1.22) | 0.66 (0.48, 0.91) | 0.84 (0.54, 1.32) | x | 0.9 (0.29, 2.75) | x | x |
| 7 – 9 | 0.81 (0.47, 1.4) | 0.81 (0.44, 1.47) | 0.74 (0.53, 1.02) | 0.91 (0.57, 1.44) | x | 1.66 (0.57, 4.83) | x | x |
| 10 – 12 | 0.95 (0.54, 1.67) | 0.82 (0.44, 1.54) | 0.86 (0.61, 1.21) | 1.05 (0.65, 1.69) | x | 0.95 (0.3, 2.96) | x | x |
| >=13 | 0.95 (0.53, 1.69) | 1.03 (0.55, 1.91) | 0.82 (0.57, 1.16) | 1.1 (0.68, 1.78) | x | 1.9 (0.67, 5.42) | x | x |
| Cholesterol measurement** (HDL/LDL) |  | x |  |  | x | x | x | x |
| <=3.5 | ref | x | ref | ref | x | x | x | x |
| 3.6 – 5 | 1.37 (0.92, 2.03) | x | 1.21 (0.93, 1.58) |  | x | x | x | x |
| >5 | 1.8 (1.1, 2.94) | x | 1.4 (0.95, 2.08) |  | x | x | x | x |
| No. of previous fractures* |  |  | x |  | x | x | x | x |
| 0 | ref | ref | x | ref | x | x | x | x |
| 1 | 1.15 (0.78, 1.69) | 1.12 (0.87, 1.46) | x |  | x | x | x | x |
| >=2 | 0.89 (0.6, 1.32) | 0.92 (0.69, 1.21) | x |  | x | x | x | x |
| Abbreviations: OST, patients with incident diagnosis of osteoporosis; IFX, patients with incident fragility fracture; OBP, incident users of oral bisphosphonates; OR, odds ratio; CI, confidence intervals; MACE, composite outcome for the occurrence of either myocardial infarction, stroke or cardiovascular disease death; MI, myocardial infarction; * ever; ** in the year prior to start; SES, socio-economic status; BMI, body mass index; eGFR, estimated Glomerular Filtration Rate; SBP, cholesterol, systolic blood pressure; DBP, diastolic blood pressure. | | | | | | | | |

# Table S6b. Risk factors selected by lasso for one- and two-year models in gender based models (IFX cohort)

| IFX cohort  Predictor | Women OR (95%CI) | | | | Men OR (95%CI) | | | |
| --- | --- | --- | --- | --- | --- | --- | --- | --- |
|  | One year | | Two year | | One year | | Two year | |
|  | MACE | Stroke/MI | MACE | Stroke/MI | MACE | Stroke/MI | MACE | Stroke/MI |
| SES | x |  | x |  | x | x | x | x |
| 1 | x | ref | x | ref | x | x | x | x |
| 2 | x | 1.21 (0.94, 1.57) | x | 1.02 (0.84, 1.25) | x | x | x | x |
| 3 | x | 1.17 (0.9, 1.52) | x | 1.01 (0.82, 1.24) | x | x | x | x |
| 4 | x | 1.35 (1.04, 1.77) | x | 1.11 (0.89, 1.38) | x | x | x | x |
| 5 | x | 1.56 (1.17, 2.06) | x | 1.28 (1.01, 1.61) | x | x | x | x |
| Smoking** | x | x | x | x | x | x |  | x |
| Ex | x | x | x | x | x | x | ref | x |
| No | x | x | x | x | x | x | 0.97 (0.75, 1.25) | x |
| Yes | x | x | x | x | x | x | 1.26 (0.93, 1.7) | x |
| Drinking** |  | x |  |  | x | x |  | x |
| Ex | ref | x | ref | ref | x | x | ref | x |
| No | 1.07 (0.74, 1.56) | 1.01 (0.63, 1.62) | 1.06 (0.77, 1.45) | 0.95 (0.66, 1.38) | x | x | 1.03 (0.65, 1.62) | x |
| Yes | 1.04 (0.69, 1.59) | 0.97 (0.58, 1.62) | 1.01 (0.74, 1.39) | 0.95 (0.6, 1.51) | x | x | 0.96 (0.61, 1.49) | x |
| Diabetes type I* | x | x | x | x | x | x | x | x |
| Diabetes type II* | x | 1.26 (0.82, 1.93) | x | 1.39 (0.97, 1.99) | x | x | x | x |
| Chronic obstructive pulmonary disease* | x | x | x | x | x | x | 0.87 (0.61, 1.24) | x |
| Chronic kidney disease* | x | x | x | 0.92 (0.41, 2.1) | x | x | x | x |
| Rheumatoid arthritis* | x | x | x | x | x | x | x | x |
| Lupus* | x | x | x | x | x | x | x | x |
| Systemic heart disease** | x | x | x | x | x | x | x | x |
| Anti-osteoporosis use** | x | x | x | x | x | x | x | x |
| Heparin use** | x | x | x | x | x | x | x | x |
| Beta-blocker use** | 1.1 (0.93, 1.31) | 1.06 (0.83, 1.34) | x | 1.18 (0.97, 1.43) | x | x | x | x |
| Hypertension** | x | x | x | x | x | x | x | x |
| Deep vein thrombosis or pulmonary embolism** | x | x | x | x | x | x | x | x |
| Anticoagulant use** | x | x | x | x | x | x | x | x |
| Antidepressants TCA** | x | x | x | x | x | x | x | x |
| Antidepressants SSRI** | x | x | x | x | x | x | x | x |
| Hypercholesterolemia** | x | x | x | x | x | x | x | x |
| Statin use** | x | x | x | x | x | x | 0.82 (0.59, 1.14) | x |
| Osteoporosis history* | x | x | x | 0.93 (0.67, 1.28) | x | x | x | x |
| Family history of cardiovascular disease (%) | x | x | x | x | x | x | x | x |
| Family history of cardiovascular disease before age 60 | x | x | x | x | x | x | x | x |
| Heart failure* | x | x | x | 1.02 (0.72, 1.44) | x | x | x | x |
| Migraine* | x | x | x | x | x | x | x | x |
| Severe mental illness* | x | x | x | x | x | x | x | x |
| Vascular Disease* | X | x | x | x | x | x | x | x |
| Atrial fibrillation* | 1.34 (1.08, 1.65) | 1.46 (1.13, 1.9) | x | 1.22 (0.97, 1.54) | x | x | x | x |
| On anti-hypertensive drug | 1.25 (0.96, 1.61) | 1.15 (0.78, 1.71) | 1.19 (0.94, 1.51) | 1.07 (0.78, 1.46) | 1.19 (0.87, 1.64) | 1.92 (1.13, 3.27) | 1.36 (1.01, 1.83) | 1.31 (0.98, 1.76) |
| Antipsychotic use** | x | x | x | x | x | x | x | x |
| Steroid use** | x | x | x | x | x | x | 0.78 (0.55, 1.11) | x |
| Erectile dysfunction** | x | x | x | x | x | x | x | x |
| Age Group (%) |  |  |  |  |  | x |  |  |
| 50-59 | ref | ref | ref | ref | ref | x | ref | ref |
| 60-69 | 4 (1.4, 11.43) | 2.55 (0.85, 7.6) | 1.71 (0.88, 3.33) | 4.99 (1.73, 14.37) | 5.24 (1.84, 14.9) | x | 4.17 (1.98, 8.8) | 2.61 (1.27, 5.35) |
| 70-79 | 8.86 (3.2, 24.56) | 6.49 (2.16, 19.53) | 5.06 (2.65, 9.66) | 11.91 (3.87, 36.64) | 7.06 (2.51, 19.85) | x | 6.31 (2.9, 13.72) | 3.37 (1.66, 6.85) |
| 80-89 | 13.47 (4.72, 38.48) | 9.04 (2.69, 30.41) | 7.62 (3.75, 15.51) | 18.01 (5.06, 64.07) | 11.3 (3.95, 32.35) | x | 9.76 (4.18, 22.77) | 4.61 (2.19, 9.73) |
| >89 | 18.16 (6.06, 54.4) | 8.58 (2.21, 33.29) | 9.36 (4.22, 20.78) | 18 (4.31, 75.18) | 12.26 (4.05, 37.09) | x | 9.58 (3.68, 24.97) | 3.49 (1.56, 7.8) |
| Charlson score | x | x | x | x | x | x |  | x |
| 0 | x | x | x | x | x | x | ref | x |
| 1 | x | x | x | x | x | x | 1.14 (0.89, 1.48) | x |
| 2 | x | x | x | x | x | x | 0.87 (0.61, 1.25) | x |
| ≥3 | x | x | x | x | x | x | 0.86 (0.55, 1.36) | x |
| Cardiovascular disease | x | x | x |  | x | x |  | x |
| No | x | x | x | ref | x | x | ref | x |
| Ever >1 year before index date | x | x | x | 1.32 (1.05, 1.65) | x | x | 0.91 (0.69, 1.19) | x |
| 1 year before index | x | x | x | 0.96 (0.53, 1.72) | x | x | 1.86 (1.08, 3.19) | x |
| 6 months before index | x | x | x | 1.81 (1.15, 2.85) | x | x | 1.26 (0.67, 2.37) | x |
| 1 month before index | x | x | x | 2.09 (0.85, 5.13) | x | x | 1.91 (0.7, 5.22) | x |
| MI or Stroke | x |  | x |  | x | x | x |  |
| No | x | ref | x | ref | x | x | x | ref |
| Ever >1 year before index date | x | 0.95 (0.65, 1.39) | x | 1.15 (0.85, 1.57) | x | x | x | 1.63 (1.05, 2.52) |
| 1 year before index | x | 1.91 (1.26, 2.89) | x | 1.32 (0.9, 1.92) | x | x | x | 1.74 (1, 3.03) |
| Established CVD * | 1.77 (1.51, 2.07) | 1.5 (1.14, 1.97) | 1.77 (1.54, 2.04) | 1.48 (1.16, 1.9) | 1.91 (1.55, 2.37) | 1.66 (1.21, 2.3) | 2.21 (1.77, 2.75) | 1.26 (0.86, 1.84) |
| Any fracture history | X | x | x | x | x | x | x | x |
| No | x | x | x | x | x | x | x | x |
| Ever >1 year before index date | x | x | x | x | x | x | x | x |
| 1 year before index | x | x | x | x | x | x | x | x |
| Hip fracture history | x | x | x | x | x | x | x | x |
| No | x | x | x | x | x | x | x | x |
| Ever >1 year before index date | x | x | x | x | x | x | x | x |
| 1 year before index | x | x | x | x | x | x | x | x |
| Shoulder fracture history | x | x | x | x | x | x | x | x |
| No | x | x | x | x | x | x | x | x |
| Ever >1 year before index date | x | x | x | x | x | x | x | x |
| 1 year before index | x | x | x | x | x | x | x | x |
| Spine fracture history | x | x | x | x | x | x | x | x |
| No | x | x | x | x | x | x | x | x |
| Ever >1 year before index date | x | x | x | x | x | x | x | x |
| 1 year before index | x | x | x | x | x | x | x | x |
| Wrist fracture history | x | x | x | x | x | x | x | x |
| No | x | x | x | x | x | x | x | x |
| Ever >1 year before index date | x | x | x | x | x | x | x | x |
| 1 year before index | x | x | x | x | x | x | x | x |
| BMI** | x |  | x |  | x | x |  | x |
| <18.5 | x | ref | x | ref | x | x | ref | x |
| 18.6 - 24.9 | x | 1.08 (0.48, 2.44) | x | 1.11 (0.44, 2.79) | x | x | 0.94 (0.53, 1.68) | x |
| 25 - 29.9 | x | 1.18 (0.27, 5.13) | x | 1.22 (0.22, 6.65) | x | x | 0.99 (0.41, 2.42) | x |
| 30 - 39.9 | x | 1.32 (0.16, 10.98) | x | 1.56 (0.14, 16.82) | x | x | 1 (0.27, 3.65) | x |
| >=40 | x | 2.35 (0.1, 54.93) | x | 2.55 (0.05, 118.47) | x | x | 1.57 (0.1, 25.96) | x |
| No. of GP visits** | x |  | x | x | x | x |  | x |
| 0 | x | ref | x | x | x | x | ref | x |
| 1-5 | x | 0.74 (0.47, 1.17) | x | x | x | x | 0.93 (0.68, 1.27) | x |
| 6-10 | x | 0.69 (0.42, 1.11) | x | x | x | x | 0.71 (0.51, 1.01) | x |
| 11-15 | x | 0.66 (0.4, 1.1) | x | x | x | x | 0.86 (0.6, 1.23) | x |
| >=16 | x | 0.73 (0.45, 1.2) | x | x | x | x | 0.75 (0.53, 1.06) | x |
| No. of GP emergency visits** | x | x | x | x | x | x | x | x |
| 0 | x | x | x | x | x | x | x | x |
| 1 | x | x | x | x | x | x | x | x |
| 2 | x | x | x | x | x | x | x | x |
| 3-5 | x | x | x | x | x | x | x | x |
| >=6 | x | x |  | x | x | x | x | x |
| eGFR** |  |  |  |  |  |  |  | x |
| <=29 | ref | ref | ref | ref | ref | ref | ref | x |
| 30 – 44 | 0.78 (0.52, 1.16) | 0.87 (0.5, 1.49) | 0.96 (0.65, 1.43) | 0.95 (0.53, 1.72) | 0.84 (0.42, 1.67) | 0.76 (0.26, 2.21) | 0.89 (0.48, 1.65) | x |
| 45 – 59 | 0.65 (0.35, 1.2) | 0.7 (0.27, 1.81) | 0.79 (0.37, 1.66) | 0.7 (0.23, 2.12) | 0.66 (0.32, 1.37) | 0.65 (0.2, 2.07) | 0.72 (0.3, 1.71) | x |
| 60 – 89 | 0.54 (0.15, 1.96) | 0.69 (0.08, 5.86) | 0.69 (0.15, 3.13) | 0.69 (0.05, 10.07) | 0.57 (0.17, 1.92) | 0.51 (0.06, 4.31) | 0.62 (0.14, 2.72) | x |
| >=90 | 0.48 (0.11, 2.12) | 0.51 (0.04, 6.41) | 0.7 (0.14, 3.57) | 0.67 (0.05, 8.47) | 0.35 (0.07, 1.79) | 0.3 (0.02, 3.9) | 0.45 (0.09, 2.37) | x |
| SBP** | x |  |  |  | x | x |  |  |
| <120 | x | ref | ref | ref | x | x | ref | ref |
| 120 - 139 | x | 1.04 (0.74, 1.47) | 1.02 (0.83, 1.26) | 1.11 (0.82, 1.49) | x | x | 0.95 (0.72, 1.24) | 1.19 (0.79, 1.79) |
| 140 - 159 | x | 1.33 (0.91, 1.93) | 1.11 (0.88, 1.41) | 1.17 (0.82, 1.65) | x | x | 1.2 (0.89, 1.61) | 1.6 (1.05, 2.44) |
| >=160 | x | 1.42 (0.84, 2.38) | 1.39 (0.97, 2.01) | 1.25 (0.77, 2.04) | x | x | 1.39 (0.89, 2.16) | 2.77 (1.62, 4.73) |
| DBP** | x | x | x |  | x | x | x | x |
| <80 | x | x | x | ref | x | x | x | x |
| 80 – 89 | x | x | x | 1.08 (0.91, 1.29) | x | x | x | x |
| 90 – 99 | x | x | x | 1.18 (0.85, 1.63) | x | x | x | x |
| >=100 | x | x | x | 1.34 (0.8, 2.23) | x | x | x | x |
| No. of concomitant medicines** | x |  |  |  | x | x | x | x |
| 0 | x | ref | ref | ref | x | x | x | x |
| 1 – 3 | x | 1.1 (0.66, 1.82) | 0.91 (0.73, 1.12) | 0.99 (0.76, 1.28) | x | x | x | x |
| 4 – 6 | x | 1.41 (0.85, 2.32) | 1.05 (0.86, 1.29) | 0.95 (0.73, 1.23) | x | x | x | x |
| 7 – 9 | x | 1.61 (0.96, 2.72) | 1.13 (0.91, 1.41) | 1.03 (0.79, 1.34) | x | x | x | x |
| 10 – 12 | x | 1.71 (0.99, 2.97) | 1.17 (0.92, 1.49) | 0.96 (0.72, 1.29) | x | x | x | x |
| >=13 | x | 2.17 (1.21, 3.88) | 1.21 (0.93, 1.57) | 1 (0.72, 1.4) | x | x | x | x |
| Cholesterol measurement** (HDL/LDL) |  |  |  |  | x |  |  |  |
| <=3.5 | ref | ref | ref | ref | x | ref | ref | ref |
| 3.6 – 5 | 1.06 (0.54, 2.08) | 1.06 (0.29, 3.86) | 1.14 (0.58, 2.24) | 1.07 (0.29, 3.95) | x | 0.96 (0.31, 2.91) | 0.91 (0.43, 1.93) | 1.04 (0.29, 3.76) |
| >5 | 1.43 (0.33, 6.24) | 1.45 (0.13, 16.46) | 1.44 (0.28, 7.5) | 1.37 (0.1, 19.2) | x | 1.85 (0.34, 10.09) | 1.26 (0.27, 5.9) | 1.92 (0.25, 14.9) |
| No. of previous fractures* | x | x | x | x | x | x | x | x |
| 0 | x | x | x | x | x | x | x | x |
| 1 | x | x | x | x | x | x | x | x |
| >=2 | x | x | x | x | x | x | x | x |
| Abbreviations: OST, patients with incident diagnosis of osteoporosis; IFX, patients with incident fragility fracture; OBP, incident users of oral bisphosphonates; OR, odds ratio; CI, confidence intervals; MACE, composite outcome for the occurrence of either myocardial infarction, stroke or cardiovascular disease death; MI, myocardial infarction; * ever; ** in the year prior to start; SES, socio-economic status; BMI, body mass index; eGFR, estimated Glomerular Filtration Rate; SBP, cholesterol, systolic blood pressure; DBP, diastolic blood pressure. | | | | | | | | |

# Table S6c. Risk factors selected by lasso for one- and two-year models in gender based models (OBP cohort)

| OBP cohort  Predictor | Women OR (95%CI) | | | | Men OR (95%CI) | | | |
| --- | --- | --- | --- | --- | --- | --- | --- | --- |
|  | One year | | Two year | | One year | | Two year | |
|  | MACE | Stroke/MI | MACE | Stroke/MI | MACE | Stroke/MI | MACE | Stroke/MI |
| SES |  |  |  |  | x | x |  | x |
| 1 | ref | ref | ref | x | x | x | ref | x |
| 2 | 0.99 (0.84, 1.17) | 1.14 (0.94, 1.38) | 1.07 (0.95, 1.22) | x | x | x | 1.11 (0.9, 1.37) | x |
| 3 | 1.18 (1, 1.39) | 1.34 (1.11, 1.61) | 1.25 (1.1, 1.42) | x | x | x | 1.09 (0.88, 1.35) | x |
| 4 | 1.14 (0.95, 1.36) | 1.26 (1.03, 1.54) | 1.19 (1.04, 1.37) | x | x | x | 1.26 (1.01, 1.56) | x |
| 5 | 1.27 (1.05, 1.54) | 1.24 (0.99, 1.56) | 1.2 (1.03, 1.4) | x | x | x | 1.1 (0.85, 1.43) | x |
| Smoking** | x |  |  |  | x |  | x | x |
| Ex | x | ref | ref | ref | x | ref | x | x |
| No | x | 0.83 (0.61, 1.13) | 0.88 (0.74, 1.04) | 0.91 (0.74, 1.11) | x | 0.97 (0.68, 1.41) | x | x |
| Yes | x | 0.91 (0.67, 1.24) | 1.09 (0.88, 1.35) | 1.23 (0.96, 1.58) | x | 1.17 (0.78, 1.75) | x | x |
| Drinking** |  |  |  |  | x | x |  | x |
| Ex | ref | ref | ref | ref | x | x | ref | x |
| No | 0.95 (0.64, 1.42) | 1.05 (0.69, 1.6) | 0.88 (0.65, 1.2) | 0.9 (0.67, 1.2) | x | x | 0.84 (0.58, 1.22) | x |
| Yes | 0.82 (0.57, 1.19) | 0.87 (0.59, 1.28) | 0.78 (0.57, 1.05) | 0.78 (0.59, 1.04) | x | x | 0.81 (0.58, 1.14) | x |
| Diabetes type I* | x | x | x | x | x | x | x | x |
| Diabetes type II* | 1.45 (1.15, 1.84) | 1.49 (1.18, 1.9) | 1.36 (1.14, 1.63) | x | x | x | x | x |
| Chronic obstructive pulmonary disease* | x | 1.26 (0.97, 1.64) | x | x | x | x | x | x |
| Chronic kidney disease* | 0.81 (0.61, 1.08) | x | 0.86 (0.7, 1.07) | x | x | x | x | x |
| Rheumatoid arthritis* | x | x | 1 (0.86, 1.16) | x | 0.57 (0.42, 0.76) | 0.73 (0.52, 1.02) | x | x |
| Lupus* | x | x | x | x | x | x | x | x |
| Systemic heart disease** | x | x | x | x | x | x | x | x |
| Anti-osteoporosis use** | x | x | x | x | x | x | x | x |
| Heparin use** | x | x | x | x | x | x | x | x |
| Beta-blocker use** | 1.08 (0.93, 1.25) | 1.25 (1.06, 1.48) | 1.19 (1.06, 1.34) | 1.17 (1.03, 1.32) | x | 1.04 (0.8, 1.37) | x | x |
| Hypertension** | x | x | x | x | x | x | x | x |
| Deep vein thrombosis or pulmonary embolism** | x | x | x | x | x | x | x | x |
| Anticoagulant use** | x | x | x | x | x | x | x | x |
| Antidepressants TCA** | x | x | x | x | x | x | x | x |
| Antidepressants SSRI** | x | 1.29 (1.06, 1.56) | 1.16 (1.01, 1.34) | x | x | x | x | x |
| Hypercholesterolemia** | x | x | x | x | x | x | x | x |
| Statin use** | x | 0.84 (0.69, 1.01) | x | x | x | x | x | x |
| Osteoporosis history* | 0.76 (0.66, 0.86) | 0.77 (0.66, 0.89) | 0.77 (0.7, 0.85) | 0.85 (0.76, 0.95) | 0.84 (0.66, 1.06) | x | 0.83 (0.69, 1.01) | 0.82 (0.66, 1.01) |
| Family history of cardiovascular disease | x | x | 0.99 (0.84, 1.16) | x | x | x | x | x |
| Family history of cardiovascular disease before age 60 | x | x | x | x | x | x | x | x |
| Heart failure* | x | x | x | x | x | 1.43 (1.01, 2.05) | x | x |
| Migraine* | x | x | x | x | x | x |  | x |
| Severe mental illness* | x | x | x | x | x | x | x | x |
| Vascular Disease* | x | x | x | x | x | x | x | x |
| Atrial fibrillation* | 1.57 (1.3, 1.88) | 1.45 (1.17, 1.78) | 1.52 (1.31, 1.76) | 1.4 (1.19, 1.64) | x | x | x | x |
| On anti-hypertensive drug | x | 1.14 (0.94, 1.39) | 1.09 (0.96, 1.25) | 1.17 (1.01, 1.36) | x | x | 1.14 (0.93, 1.4) | 1.11 (0.88, 1.39) |
| Antipsychotic use** | x | x | x | x | x | x | x | x |
| Steroid use** | x | 1.05 (0.89, 1.25) | x | x | x | 0.99 (0.77, 1.26) | 0.89 (0.75, 1.06) | x |
| Erectile dysfunction** | x | x | x | x | x | x | x | x |
| Age Group |  |  |  |  |  |  |  |  |
| 50-59 | ref | ref | ref | ref | ref | ref | ref | ref |
| 60-69 | 2.31 (1.42, 3.76) | 1.86 (1.19, 2.9) | 1.62 (1.17, 2.23) | 1.8 (1.27, 2.55) | 1.58 (0.95, 2.62) | 1.26 (0.77, 2.06) | 1.39 (0.95, 2.03) | 1.2 (0.81, 1.78) |
| 70-79 | 4.33 (2.72, 6.88) | 2.68 (1.75, 4.12) | 3.3 (2.44, 4.47) | 3.4 (2.44, 4.75) | 2.39 (1.49, 3.85) | 1.48 (0.92, 2.38) | 1.95 (1.37, 2.8) | 1.96 (1.36, 2.84) |
| 80-89 | 8.6 (5.38, 13.74) | 4.91 (3.18, 7.6) | 6.33 (4.65, 8.62) | 5.76 (4.09, 8.1) | 2.8 (1.73, 4.53) | 1.81 (1.11, 2.95) | 2.53 (1.76, 3.65) | 2.01 (1.38, 2.95) |
| >89 | 9.96 (6.07, 16.35) | 5.22 (3.23, 8.43) | 8.2 (5.88, 11.45) | 6.35 (4.37, 9.23) | 5.41 (3.2, 9.17) | 2.27 (1.25, 4.12) | 3.3 (2.17, 5.02) | 2.35 (1.49, 3.72) |
| Charlson score |  | x |  | x | x |  |  | x |
| 0 | ref | x | ref | x | x | ref | ref | x |
| 1 | 0.85 (0.72, 1.01) | x | 0.94 (0.82, 1.06) | x | x | 1.02 (0.75, 1.38) | 0.89 (0.73, 1.08) | x |
| 2 | 0.9 (0.73, 1.1) | x | 0.97 (0.83, 1.13) | x | x | 1.09 (0.77, 1.53) | 0.78 (0.62, 0.99) | x |
| ≥3 | 0.98 (0.77, 1.25) | x | 0.97 (0.79, 1.18) | x | x | 1.08 (0.75, 1.56) | 0.93 (0.74, 1.18) | x |
| Cardiovascular disease |  |  | x |  | x |  | x |  |
| No | ref | ref | x | ref | x | ref | x | ref |
| Ever >1 year before index date | 1.15 (0.95, 1.38) | 1.31 (1.06, 1.61) | x | 1.38 (1.19, 1.61) | x | 1.42 (1.05, 1.93) | x | 1.26 (1, 1.58) |
| 1 year before index | 1.11 (0.73, 1.69) | 0.98 (0.6, 1.61) | x | 1.09 (0.74, 1.59) | x | 1.83 (1.03, 3.27) | x | 1.17 (0.69, 2) |
| 6 months before index | 1.24 (0.85, 1.82) | 1.5 (1, 2.24) | x | 1.5 (1.09, 2.08) | x | 3.03 (1.9, 4.85) | x | 2.69 (1.86, 3.88) |
| 1 month before index | 2.15 (1.32, 3.49) | 2.84 (1.75, 4.61) | x | 2.14 (1.35, 3.38) | x | 3.66 (1.91, 7.03) | x | 2.51 (1.39, 4.53) |
| MI or Stroke |  |  | x |  | x |  |  | x |
| No | ref | ref | ref | ref | x | ref | ref | x |
| Ever >1 year before index date | 1.31 (1.02, 1.68) | 1.03 (0.77, 1.38) | 1.4 (1.15, 1.69) | 1.28 (1.03, 1.58) | x | 0.76 (0.5, 1.17) | 1.35 (1.02, 1.78) | x |
| 1 year before index | 2.09 (1.59, 2.77) | 2.15 (1.57, 2.95) | 1.98 (1.59, 2.47) | 1.64 (1.27, 2.12) | x | 1.88 (1.19, 2.98) | 2.33 (1.68, 3.23) | x |
| Established CVD * | 1.58 (1.28, 1.96) | 1.41 (1.11, 1.79) | 1.64 (1.4, 1.91) | 1.37 (1.15, 1.64) | 2.3 (1.89, 2.8) | 1.38 (0.98, 1.94) | 1.71 (1.35, 2.16) | 1.49 (1.22, 1.82) |
| Any fracture history | x | x | x | x | x | x | x | x |
| No | x | x | x | x | x | x | x | x |
| Ever >1 year before index date | x | x | x | x | x | x | x | x |
| 1 year before index | x | x | x | x | x | x | x | x |
| Hip fracture history | x | x | x | x | x | x |  | x |
| No | x | x | x | x | x | x | ref | x |
| Ever >1 year before index date | x | x | x | x | x | x | 0.9 (0.43, 1.87) | x |
| 1 year before index | x | x | x | x | x | x | 1.18 (0.91, 1.54) | x |
| Shoulder fracture history | x | x | x | x | x | x | x | x |
| No | x | x | x | x | x | x | x | x |
| Ever >1 year before index date | x | x | x | x | x | x | x | x |
| 1 year before index | x | x | x | x | x | x | x | x |
| Spine fracture history | x | x | x | x | x | x | x | x |
| No | x | x | x | x | x | x | x | x |
| Ever >1 year before index date | x | x | x | x | x | x | x | x |
| 1 year before index | x | x | x | x | x | x | x | x |
| Wrist fracture history | x | x | x | x | x | x | x | x |
| No | x | x | x | x | x | x | x | x |
| Ever >1 year before index date | x | x | x | x | x | x | x | x |
| 1 year before index | x | x | x | x | x | x | x | x |
| BMI** |  |  |  |  | x |  |  |  |
| <18.5 | ref | ref | ref | ref | x | ref | ref | ref |
| 18.6 - 24.9 | 0.72 (0.54, 0.96) | 0.78 (0.58, 1.04) | 0.75 (0.61, 0.91) | 0.79 (0.64, 0.97) | x | 1.11 (0.59, 2.07) | 0.83 (0.58, 1.2) | 0.92 (0.6, 1.39) |
| 25 - 29.9 | 0.58 (0.4, 0.84) | 0.68 (0.47, 0.98) | 0.58 (0.44, 0.75) | 0.67 (0.51, 0.88) | x | 0.87 (0.44, 1.74) | 0.72 (0.48, 1.08) | 0.82 (0.53, 1.27) |
| 30 - 39.9 | 0.54 (0.34, 0.86) | 0.59 (0.37, 0.93) | 0.58 (0.43, 0.78) | 0.68 (0.49, 0.93) | x | 0.78 (0.39, 1.57) | 0.57 (0.37, 0.9) | 0.76 (0.47, 1.23) |
| >=40 | 0.42 (0.17, 1.04) | 0.59 (0.25, 1.42) | 0.61 (0.32, 1.15) | 0.73 (0.37, 1.44) | x | 0.89 (0.24, 3.39) | 0.81 (0.31, 2.14) | 0.83 (0.29, 2.34) |
| No. of GP visits** |  |  | x |  | x |  |  |  |
| 0 | ref | ref | x | ref | x | ref | ref | ref |
| 1-5 | 0.95 (0.76, 1.2) | 0.85 (0.65, 1.12) | x | 0.91 (0.74, 1.12) | x | 0.52 (0.3, 0.9) | 1.06 (0.76, 1.5) | 0.97 (0.65, 1.44) |
| 6-10 | 0.84 (0.64, 1.11) | 0.87 (0.63, 1.2) | x | 0.84 (0.66, 1.06) | x | 0.56 (0.31, 1.04) | 0.88 (0.59, 1.31) | 0.69 (0.44, 1.08) |
| 11-15 | 0.7 (0.52, 0.94) | 0.79 (0.56, 1.11) | x | 0.77 (0.6, 0.99) | x | 0.55 (0.29, 1.03) | 0.89 (0.59, 1.34) | 0.75 (0.47, 1.19) |
| >=16 | 0.79 (0.59, 1.06) | 0.86 (0.62, 1.2) | x | 0.88 (0.68, 1.12) | x | 0.69 (0.37, 1.27) | 1.14 (0.76, 1.7) | 0.87 (0.55, 1.36) |
| No. of GP emergency visits** |  |  |  | x | x | x | x | x |
| 0 | ref | ref | ref | x | x | x | x | x |
| 1 | 1.09 (0.91, 1.31) | 1.09 (0.89, 1.33) | 1.17 (1.02, 1.34) | x | x | x | x | x |
| 2 | 1.32 (1.03, 1.7) | 1.36 (1.05, 1.78) | 1.33 (1.11, 1.61) | x | x | x | x | x |
| 3-5 | 1.39 (1.09, 1.78) | 1.09 (0.82, 1.45) | 1.19 (0.99, 1.44) | x | x | x | x | x |
| >=6 | 2.41 (1.8, 3.24) | 1.7 (1.2, 2.43) | 1.64 (1.26, 2.12) | x | x | x | x | x |
| eGFR** |  |  |  |  |  |  |  |  |
| <=29 | ref | ref | ref | ref | ref | ref | ref | ref |
| 30 – 44 | 0.89 (0.51, 1.54) | 0.94 (0.49, 1.82) | 0.93 (0.6, 1.44) | 0.88 (0.54, 1.42) | 1.43 (0.5, 4.1) | 1.21 (0.35, 4.21) | 1.38 (0.6, 3.17) | 1.57 (0.53, 4.68) |
| 45 – 59 | 0.8 (0.43, 1.48) | 0.8 (0.38, 1.68) | 0.82 (0.5, 1.35) | 0.76 (0.46, 1.26) | 1.29 (0.46, 3.63) | 1.29 (0.35, 4.73) | 1.17 (0.51, 2.65) | 1.31 (0.47, 3.67) |
| 60 – 89 | 0.56 (0.25, 1.23) | 0.58 (0.22, 1.52) | 0.64 (0.34, 1.2) | 0.6 (0.32, 1.13) | 1.05 (0.34, 3.24) | 1.05 (0.24, 4.59) | 0.99 (0.42, 2.34) | 1.1 (0.38, 3.17) |
| >=90 | 0.58 (0.2, 1.64) | 0.53 (0.19, 1.5) | 0.63 (0.29, 1.37) | 0.54 (0.28, 1.06) | 0.79 (0.21, 3.06) | 0.78 (0.15, 4.15) | 0.82 (0.28, 2.35) | 0.81 (0.2, 3.23) |
| SBP** |  |  |  |  |  |  |  |  |
| <120 | ref | ref | ref | ref | ref | ref | ref | ref |
| 120 - 139 | 1.02 (0.81, 1.27) | 0.94 (0.74, 1.19) | 1.14 (0.97, 1.33) | 1.06 (0.88, 1.29) | 0.94 (0.7, 1.27) | 1.34 (0.94, 1.9) | 1.03 (0.82, 1.31) | 1.3 (0.99, 1.69) |
| 140 - 159 | 1.06 (0.82, 1.38) | 1.05 (0.82, 1.34) | 1.18 (0.99, 1.4) | 1.14 (0.93, 1.41) | 1.19 (0.87, 1.62) | 1.76 (1.21, 2.56) | 1.34 (1.05, 1.71) | 1.55 (1.17, 2.05) |
| >=160 | 1.2 (0.88, 1.66) | 1.29 (0.95, 1.74) | 1.42 (1.16, 1.75) | 1.43 (1.15, 1.79) | 1.46 (1, 2.13) | 1.7 (1.05, 2.73) | 1.51 (1.11, 2.06) | 1.94 (1.37, 2.74) |
| DBP** |  |  |  | x | x | x | x | x |
| <80 | ref | ref | ref | x | x | x | x | x |
| 80 – 89 | 0.99 (0.84, 1.16) | 0.93 (0.77, 1.12) | 0.96 (0.86, 1.08) | x | x | x | x | x |
| 90 – 99 | 1.15 (0.89, 1.5) | 1.16 (0.89, 1.51) | 0.96 (0.78, 1.19) | x | x | x | x | x |
| >=100 | 1.54 (1.04, 2.27) | 1 (0.6, 1.65) | 1.36 (1, 1.84) | x | x | x | x | x |
| No. of concomitant medicines** |  |  |  |  | x |  |  |  |
| 0 | ref | ref | ref | ref | x | ref | ref | ref |
| 1 – 3 | 0.94 (0.74, 1.19) | 0.86 (0.64, 1.15) | 0.71 (0.6, 0.84) | 0.82 (0.66, 1.02) | x | 1.1 (0.61, 1.97) | 0.79 (0.56, 1.13) | 0.69 (0.45, 1.05) |
| 4 – 6 | 0.87 (0.67, 1.12) | 0.83 (0.61, 1.15) | 0.67 (0.56, 0.8) | 0.88 (0.69, 1.11) | x | 0.93 (0.5, 1.73) | 0.78 (0.54, 1.15) | 0.94 (0.61, 1.45) |
| 7 – 9 | 1.07 (0.81, 1.4) | 0.96 (0.68, 1.35) | 0.81 (0.67, 0.97) | 0.98 (0.76, 1.26) | x | 1.27 (0.68, 2.38) | 0.91 (0.61, 1.35) | 1.08 (0.69, 1.69) |
| 10 – 12 | 1.24 (0.93, 1.66) | 0.98 (0.68, 1.42) | 0.91 (0.74, 1.1) | 1.16 (0.89, 1.51) | x | 1.7 (0.9, 3.23) | 0.96 (0.64, 1.46) | 1.19 (0.75, 1.9) |
| >=13 | 1.28 (0.95, 1.73) | 1.11 (0.76, 1.62) | 0.88 (0.72, 1.08) | 1.21 (0.92, 1.6) | x | 1.66 (0.87, 3.2) | 0.98 (0.64, 1.5) | 1.33 (0.83, 2.12) |
| Cholesterol measurement** (HDL/LDL) |  |  |  | x | x |  | x | x |
| <=3.5 | ref | ref | ref | x | x | ref | x | x |
| 3.6 – 5 | 1.18 (0.95, 1.48) | 1.15 (0.91, 1.46) | 1.13 (0.95, 1.34) | x | x | 1.21 (0.82, 1.78) | x | x |
| >5 | 1.44 (0.96, 2.18) | 1.28 (0.82, 2) | 1.39 (1.02, 1.9) | x | x | 1.56 (0.85, 2.86) |  | x |
| No. of previous fractures* | x |  | x | x | x | x | x | x |
| 0 | x | ref | x | x | x | x | x | x |
| 1 | x | 1.09 (0.91, 1.31) | x | x | x | x | x | x |
| >=2 | x | 1.01 (0.83, 1.23) | x | x | x | x | x | x |
| Abbreviations: OST, patients with incident diagnosis of osteoporosis; IFX, patients with incident fragility fracture; OBP, incident users of oral bisphosphonates; OR, odds ratio; CI, confidence intervals; MACE, composite outcome for the occurrence of either myocardial infarction, stroke or cardiovascular disease death; MI, myocardial infarction; * ever; ** in the year prior to start; SES, socio-economic status; BMI, body mass index; eGFR, estimated Glomerular Filtration Rate; SBP, cholesterol, systolic blood pressure; DBP, diastolic blood pressure. | | | | | | | | |

# Table S7a. Model equations from lasso selection for women and men based models (OST cohort)

| OST cohort  Predictor | Women Beta coefficients | | | | Men Beta coefficients | | | |
| --- | --- | --- | --- | --- | --- | --- | --- | --- |
|  | One year | | Two year | | One year | | Two year | |
|  | MACE | Stroke/MI | MACE | Stroke/MI | MACE | Stroke/MI | MACE | Stroke/MI |
| Intercept | -4.047 | -4.538 | -4.560 | -3.974 | -4.267 | -4.134 | -4.577 | -3.165 |
| SES |  | x |  | x | x | x | x | x |
| 1 | ref | x | ref | x | x | x | x | x |
| 2 | -0.083 | x | 0.014 | x | x | x | x | x |
| 3 | 0.003 | x | 0.144 | x | x | x | x | x |
| 4 | 0.030 | x | -0.006 | x | x | x | x | x |
| 5 | -0.046 | x | 0.298 | x | x | x | x | x |
| Smoking** |  | x |  | x | x | x | x | x |
| Ex | ref | x | ref | x | x | x | x | x |
| No | 0.009 | x | 0.032 | x | x | x | x | x |
| Yes | 0.414 | x | 0.280 | x | x | x | x | x |
| Drinking** |  |  |  |  | x | x | x | x |
| Ex | ref | ref | ref | ref | x | x | x | x |
| No | 0.031 | 0.022 | -0.036 | -0.014 | x | x | x | x |
| Yes | -0.226 | -0.283 | -0.186 | -0.168 | x | x | x | x |
| Diabetes type I* | x | x | x | x | x | x | x | x |
| Diabetes type II* | 0.343 | x | x | x | x | x | x | x |
| Chronic obstructive pulmonary disease* | x | x | x | x | x | x | x | x |
| Chronic kidney disease* | x | x | -0.124 | x | x | x | x | x |
| Rheumatoid arthritis* | x | x | x | x | x | x | x | x |
| Lupus* | x | x | x | x | x | x | x | x |
| Systemic heart disease** | x | x | x | x | x | x | x | x |
| Anti-osteoporosis use** |  | x | 0.111 | x | x | x | x | x |
| Heparin use** | x | x | x | x | x | x | x | x |
| Beta-blocker use** | x | 0.391 | x | 0.210 | x | x | x | x |
| Hypertension** | x | x | x | x | x | x | x | x |
| Deep vein thrombosis or pulmonary embolism** | x | x | x | x | x | x | x | x |
| Anticoagulant use** | x | x | x | x | x | x | x | x |
| Antidepressants TCA** | x | x | x | x | x | x | x | x |
| Antidepressants SSRI** | 0.353 | x | 0.242 |  | x | x | x | x |
| Hypercholesterolemia** | x | x | x | x | x | x | x | x |
| Statin use** | x | x | x | x | x | x | x | x |
| Osteoporosis history* | x | x | x | x | x | x | x | x |
| Family history of cardiovascular disease (%) | x | x | x | x | x | x | x | x |
| Family history of cardiovascular disease before age 60 | x | x | x | x | x | x | x | x |
| Heart failure* | x | x | x | x | x | x | x | x |
| Migraine* | x | x | x |  | x | x | x | x |
| Severe mental illness* | x | x | x | x | x | x | x | x |
| Vascular Disease* | x | x | x | x | x | x | x | x |
| Atrial fibrillation* | 0.312 | x | 0.302 | x | x | x | x | x |
| On anti-hypertensive drug | 0.236 | 0.187 | 0.158 | 0.413 | x | x |  | x |
| Antipsychotic use** | x | x | x | x | x | x | x | x |
| Steroid use** | 0.052 | x | 0.134 | x | x | x | x | x |
| Erectile dysfunction** | x | x | x | x | x | x | x | x |
| Age Group |  |  |  |  |  | x |  | x |
| 50-59 | ref | ref | ref | ref | ref | x | ref | x |
| 60-69 | 0.034 | 0.039 | 0.801 | 0.488 | 0.548 | x | 1.093 | x |
| 70-79 | 0.942 | 1.055 | 1.520 | 1.252 | 0.420 | x | 1.240 | x |
| 80-89 | 1.539 | 1.650 | 2.063 | 1.659 | 0.789 | x | 1.539 | x |
| >89 | 1.762 | 1.906 | 2.437 | 2.008 | 0.749 | x | 1.633 | x |
| Charlson score |  | x |  |  | x | x | x | x |
| 0 | ref | x | ref | ref | x | x | x | x |
| 1 | 0.244 | x | 0.224 | 0.235 | x | x | x | x |
| 2 | -0.071 | x | 0.027 | 0.001 | x | x | x | x |
| ≥3 | 0.262 | x | 0.197 | 0.187 | x | x | x | x |
| Cardiovascular disease | x | x | x | x | x | x | x | x |
| No | x | x | x | x | x | x | x | x |
| Ever >1 year before index date | x | x | x | x | x | x | x | x |
| 1 year before index | x | x | x | x | x | x | x | x |
| 6 months before index | x | x | x | x | x | x | x | x |
| 1 month before index | x | x | x | x | x | x | x | x |
| MI or Stroke |  | x |  | x | x | x | x | x |
| No | ref | x | ref | x | x | x | x | x |
| Ever >1 year before index date | 0.284 | x | 0.263 | x | x | x | x | x |
| 1 year before index | 0.895 | x | 0.522 | x | x | x | x | x |
| Established CVD * | 0.427 | 0.791 | 0.472 | 0.699 | 0.839 | 0.783 | 0.808 | 0.828 |
| Any fracture history |  | x | x | x | x | x | x | x |
| No | ref | x | x | x | x | x | x | x |
| Ever >1 year before index date | -0.000 | x | x | x | x | x | x | x |
| 1 year before index | 0.149 | x | x | x | x | x | x | x |
| Hip fracture history | x | x | x | x | x | x | x | x |
| No | x | x | x | x | x | x | x | x |
| Ever >1 year before index date | x | x | x | x | x | x | x | x |
| 1 year before index | x | x | x | x | x | x | x | x |
| Shoulder fracture history | x | x | x | x | x | x | x | x |
| No | x | x | x | x | x | x | x | x |
| Ever >1 year before index date | x | x | x | x | x | x | x | x |
| 1 year before index | x | x | x | x | x | x | x | x |
| Spine fracture history | x | x | x | x | x | x | x | x |
| No | x | x | x | x | x | x | x | x |
| Ever >1 year before index date | x | x | x | x | x | x | x | x |
| 1 year before index | x | x | x | x | x | x | x | x |
| Wrist fracture history | x | x | x | x | x | x | x | x |
| No | x | x | x | x | x | x | x | x |
| Ever >1 year before index date | x | x | x | x | x | x | x | x |
| 1 year before index | x | x | x | x | x | x | x | x |
| BMI** |  | x |  |  | x | x | x | x |
| <18.5 | ref | x | ref | ref | x | x | x | x |
| 18.6 - 24.9 | -0.438 | x | -0.347 | -0.186 | x | x | x | x |
| 25 - 29.9 | -0.646 | x | -0.507 | -0.344 | x | x | x | x |
| 30 - 39.9 | -0.879 | x | -0.666 | -0.352 | x | x | x | x |
| >=40 | -0.647 | x | -0.107 | -0.046 | x | x | x | x |
| No. of GP visits** |  | x | x |  | x | x | x | x |
| 0 | ref | x | x | ref | x | x | x | x |
| 1-5 | -0.312 | x | x | -0.416 | x | x | x | x |
| 6-10 | -0.559 | x | x | -0.469 | x | x | x | x |
| 11-15 | -0.517 | x | x | -0.432 | x | x | x | x |
| >=16 | -0.447 | x | x | -0.315 | x | x | x | x |
| No. of GP emergency visits** |  |  |  | x | x | x | x | x |
| 0 | ref | ref | ref | x | x | x | x | x |
| 1 | 0.463 | -0.133 | 0.010 | x | x | x | x | x |
| 2 | 0.511 | -0.271 | 0.136 | x | x | x | x | x |
| 3-5 | 0.378 | -0.253 | 0.314 | x | x | x | x | x |
| >=6 | 0.739 | -0.103 | 0.646 | x | x | x | x | x |
| eGFR** |  |  |  |  | x | x |  |  |
| <=29 | ref | ref | ref | ref | x | x | ref | ref |
| 30 – 44 | -0.508 | -0.338 | -0.022 | -0.335 | x | x | 0.673 | 0.385 |
| 45 – 59 | -0.573 | -0.533 | -0.257 | -0.490 | x | x | 0.368 | -0.042 |
| 60 – 89 | -0.820 | -0.770 | -0.473 | -0.723 | x | x | 0.159 | -0.307 |
| >=90 | -0.763 | -0.591 | -0.400 | -0.663 | x | x | 0.171 | -0.531 |
| SBP** |  | x |  | x | x | x | x | x |
| <120 | ref | x | ref | x | x | x | x | x |
| 120 - 139 | 0.085 | x | -0.046 | x | x | x | x | x |
| 140 - 159 | 0.189 | x | 0.193 | x | x | x | x | x |
| >=160 | 0.262 | x | 0.279 | x | x | x | x | x |
| DBP** | x | x |  | x | x | x | x | x |
| <80 | x | x | ref | x | x | x | x | x |
| 80 – 89 | x | x | 0.073 | x | x | x | x | x |
| 90 – 99 | x | x | 0.021 | x | x | x | x | x |
| >=100 | x | x | 0.229 | x | x | x | x | x |
| No. of concomitant medicines** |  |  |  |  | x |  | x | x |
| 0 | ref | ref | ref | ref | x | ref | x | x |
| 1 – 3 | -0.608 | -0.329 | -0.441 | -0.177 | x | -1.044 | x | x |
| 4 – 6 | -0.328 | -0.387 | -0.409 | -0.173 | x | -0.111 | x | x |
| 7 – 9 | -0.209 | -0.215 | -0.308 | -0.099 | x | 0.506 | x | x |
| 10 – 12 | -0.052 | -0.193 | -0.153 | 0.048 | x | -0.052 | x | x |
| >=13 | -0.055 | 0.025 | -0.204 | 0.096 | x | 0.642 | x | x |
| Cholesterol measurement** (HDL/LDL) |  | x |  | x | x | x | x | x |
| <=3.5 | ref | x | ref | x | x | x | x | x |
| 3.6 – 5 | 0.315 | x | 0.193 | x | x | x | x | x |
| >5 | 0.586 | x | 0.339 | x | x | x | x | x |
| No. of previous fractures* |  |  | x | x | x | x | x | x |
| 0 | ref | ref | x | x | x | x | x | x |
| 1 | 0.138 | 0.117 | x | x | x | x | x | x |
| >=2 | -0.118 | -0.087 | x | x | x | x | x | x |
| Abbreviations: OST, patients with incident diagnosis of osteoporosis; IFX, patients with incident fragility fracture; OBP, incident users of oral bisphosphonates; MACE, composite outcome for the occurrence of either myocardial infarction, stroke or cardiovascular disease death; MI, myocardial infarction; * ever; ** in the year prior to start; SES, socio-economic status; BMI, body mass index; eGFR, estimated Glomerular Filtration Rate; SBP, cholesterol, systolic blood pressure; DBP, diastolic blood pressure. | | | | | | | | |

# Table S7b. Model equations from lasso selection for women and men based models (IFX cohort)

| IFX cohort  Predictor | Women Beta coefficients | | | | Men Beta coefficients | | | |
| --- | --- | --- | --- | --- | --- | --- | --- | --- |
|  | One year | | Two year | | One year | | Two year | |
|  | MACE | Stroke/MI | MACE | Stroke/MI | MACE | Stroke/MI | MACE | Stroke/MI |
| Intercept | -5.570 | -6.431 | -4.829 | -6.399 | -4.932 | -3.788 | -4.399 | -5.203 |
| SES | x |  | x |  | x | x | x | x |
| 1 | x | ref | x | ref | x | x | x | x |
| 2 | x | 0.195 | x | 0.022 | x | x | x | x |
| 3 | x | 0.157 | x | 0.009 | x | x | x | x |
| 4 | x | 0.304 | x | 0.103 | x | x | x | x |
| 5 | x | 0.442 | x | 0.245 | x | x | x | x |
| Smoking** | x | x | x | x | x | x |  | x |
| Ex | x | x | x | x | x | x | ref | x |
| No | x | x | x | x | x | x | -0.035 | x |
| Yes | x | x | x | x | x | x | 0.231 | x |
| Drinking** |  |  |  |  | x | x |  | x |
| Ex | ref | ref | ref | ref | x | x | ref | x |
| No | 0.068 | 0.010 | 0.055 | -0.047 | x | x | 0.026 | x |
| Yes | 0.044 | -0.031 | 0.012 | -0.051 | x | x | -0.045 | x |
| Diabetes type I* | x | x | x | x | x | x | x | x |
| Diabetes type II* | x | 0.232 | x | 0.329 | x | x | x | x |
| Chronic obstructive pulmonary disease* | x | x | x | x | x | x | -0.139 | x |
| Chronic kidney disease* | x | x | x | -0.078 | x | x | x | x |
| Rheumatoid arthritis* | x | x | x | x | x | x | x | x |
| Lupus* | x | x | x | x | x | x | x | x |
| Systemic heart disease** | x | x | x | x | x | x | x | x |
| Anti-osteoporosis use** | x | x | x | x | x | x | x | x |
| Heparin use** | x | x | x | x | x | x | x | x |
| Beta-blocker use** | 0.098 | 0.055 | x | 0.163 | x | x | x | x |
| Hypertension** | x | x | x | x | x | x | x | x |
| Deep vein thrombosis or pulmonary embolism** | x | x | x | x | x | x | x | x |
| Anticoagulant use** | x | x | x | x | x | x | x | x |
| Antidepressants TCA** | x | x | x | x | x | x | x | x |
| Antidepressants SSRI** | x | x | x | x | x | x | x | x |
| Hypercholesterolemia** | x | x | x | x | x | x | x | x |
| Statin use** | x | x | x | x | x | x | -0.199 | x |
| Osteoporosis history* | x | x | x | -0.076 | x | x | x | x |
| Family history of cardiovascular disease (%) | x | x | x | x | x | x | x | x |
| Family history of cardiovascular disease before age 60 | x | x | x | x | x | x | x | x |
| Heart failure* | x | x | x | 0.023 | x | x | x | x |
| Migraine* | x | x | x | x | x | x | x | x |
| Severe mental illness* | x | x | x | x | x | x | x | x |
| Vascular Disease* | X | x | x | x | x | x | x | x |
| Atrial fibrillation* | 0.290 | 0.381 | x | 0.200 | x | x | x | x |
| On anti-hypertensive drug | 0.220 | 0.142 | 0.173 | 0.067 | 0.178 | 0.654 | 0.305 | 0.269 |
| Antipsychotic use** | x | x | x | x | x | x | x | x |
| Steroid use** | x | x | x | x | x | x | -0.243 | x |
| Erectile dysfunction** | x | x | x | x | x | x | x | x |
| Age Group (%) |  |  |  |  |  | x |  |  |
| 50-59 | ref | ref | ref | ref | ref | x | ref | ref |
| 60-69 | 1.385 | 0.935 | 0.535 | 1.608 | 1.656 | x | 1.428 | 0.959 |
| 70-79 | 2.182 | 1.871 | 1.620 | 2.477 | 1.955 | x | 1.843 | 1.215 |
| 80-89 | 2.601 | 2.202 | 2.031 | 2.891 | 2.425 | x | 2.278 | 1.529 |
| >89 | 2.899 | 2.150 | 2.236 | 2.890 | 2.506 | x | 2.260 | 1.249 |
| Charlson score | x | x | x | x | x | x |  | x |
| 0 | x | x | x | x | x | x | ref | x |
| 1 | x | x | x | x | x | x | 0.135 | x |
| 2 | x | x | x | x | x | x | -0.141 | x |
| ≥3 | x | x | x | x | x | x | -0.145 | x |
| Cardiovascular disease | x | x | x |  | x | x |  | x |
| No | x | x | x | ref | x | x | ref | x |
| Ever >1 year before index date | x | x | x | 0.274 | x | x | -0.094 | x |
| 1 year before index | x | x | x | -0.0446 | x | x | 0.618 | x |
| 6 months before index | x | x | x | 0.595 | x | x | 0.232 | x |
| 1 month before index | x | x | x | 0.735 | x | x | 0.647 | x |
| MI or Stroke | x |  | x |  | x | x | x |  |
| No | x | ref | x | ref | x | x | x | ref |
| Ever >1 year before index date | x | -0.051 | x | 0.143 | x | x | x | 0.486 |
| 1 year before index | x | 0.648 | x | 0.274 | x | x | x | 0.554 |
| Established CVD * | 0.569 | 0.405 | 0.571 | 0.395 | 0.649 | 0.509 | 0.791 | 0.229 |
| Any fracture history | x | x | x | x | x | x | x | x |
| No | x | x | x | x | x | x | x | x |
| Ever >1 year before index date | x | x | x | x | x | x | x | x |
| 1 year before index | x | x | x | x | x | x | x | x |
| Hip fracture history | x | x | x | x | x | x | x | x |
| No | x | x | x | x | x | x | x | x |
| Ever >1 year before index date | x | x | x | x | x | x | x | x |
| 1 year before index | x | x | x | x | x | x | x | x |
| Shoulder fracture history | x | x | x | x | x | x | x | x |
| No | x | x | x | x | x | x | x | x |
| Ever >1 year before index date | x | x | x | x | x | x | x | x |
| 1 year before index | x | x | x | x | x | x | x | x |
| Spine fracture history | x | x | x | x | x | x | x | x |
| No | x | x | x | x | x | x | x | x |
| Ever >1 year before index date | x | x | x | x | x | x | x | x |
| 1 year before index | x | x | x | x | x | x | x | x |
| Wrist fracture history | x | x | x | x | x | x | x | x |
| No | x | x | x | x | x | x | x | x |
| Ever >1 year before index date | x | x | x | x | x | x | x | x |
| 1 year before index | x | x | x | x | x | x | x | x |
| BMI** | x |  | x |  | x | x |  | x |
| <18.5 | x | ref | x | ref | x | x | ref | x |
| 18.6 - 24.9 | x | 0.076 | x | 0.108 | x | x | -0.057 | x |
| 25 - 29.9 | x | 0.163 | x | 0.198 | x | x | -0.010 | x |
| 30 - 39.9 | x | 0.276 | x | 0.445 | x | x | -0.001 | x |
| >=40 | x | 0.856 | x | 0.934 | x | x | 0.452 | x |
| No. of GP visits** | x |  | x | x | x | x |  | x |
| 0 | x | ref | x | x | x | x | ref | x |
| 1-5 | x | -0.298 | x | x | x | x | -0.074 | x |
| 6-10 | x | -0.377 | x | x | x | x | -0.337 | x |
| 11-15 | x | -0.411 | x | x | x | x | -0.154 | x |
| >=16 | x | -0.309 | x | x | x | x | -0.288 | x |
| No. of GP emergency visits** | x | x | x | x | x | x | x | x |
| 0 | x | x | x | x | x | x | x | x |
| 1 | x | x | x | x | x | x | x | x |
| 2 | x | x | x | x | x | x | x | x |
| 3-5 | x | x | x | x | x | x | x | x |
| >=6 | x | x | x | x | x | x | x | x |
| eGFR** |  |  |  |  |  |  |  | x |
| <=29 | ref | ref | ref | ref | ref | ref | ref | x |
| 30 – 44 | -0.253 | -0.144 | -0.041 | -0.047 | -0.174 | -0.274 | -0.121 | x |
| 45 – 59 | -0.438 | -0.359 | -0.242 | -0.361 | -0.409 | -0.432 | -0.334 | x |
| 60 – 89 | -0.614 | -0.366 | -0.372 | -0.365 | -0.563 | -0.667 | -0.473 | x |
| >=90 | -0.729 | -0.671 | -0.358 | -0.400 | -1.060 | -1.191 | -0.794 | x |
| SBP** | x |  |  |  | x | x |  |  |
| <120 | x | ref | ref | ref | x | x | ref | ref |
| 120 - 139 | x | 0.040 | 0.022 | 0.100 | x | x | -0.055 | 0.174 |
| 140 - 159 | x | 0.284 | 0.106 | 0.153 | x | x | 0.181 | 0.468 |
| >=160 | x | 0.349 | 0.332 | 0.224 | x | x | 0.328 | 1.019 |
| DBP** | x | x | x |  | x | x | x | x |
| <80 | x | x | x | ref | x | x | x | x |
| 80 – 89 | x | x | x | 0.080 | x | x | x | x |
| 90 – 99 | x | x | x | 0.162 | x | x | x | x |
| >=100 | x | x | x | 0.293 | x | x | x | x |
| No. of concomitant medicines** | x |  |  |  | x | x | x | x |
| 0 | x | ref | ref | ref | x | x | x | x |
| 1 – 3 | x | 0.093 | -0.097 | -0.014 | x | x | x | x |
| 4 – 6 | x | 0.343 | 0.050 | -0.053 | x | x | x | x |
| 7 – 9 | x | 0.478 | 0.126 | 0.029 | x | x | x | x |
| 10 – 12 | x | 0.538 | 0.159 | -0.041 | x | x | x | x |
| >=13 | x | 0.774 | 0.188 | 0.003 | x | x | x | x |
| Cholesterol measurement** (HDL/LDL) |  |  |  | x | x |  |  |  |
| <=3.5 | ref | ref | ref | ref | x | ref | ref | ref |
| 3.6 – 5 | 0.060 | 0.054 | 0.131 | 0.068 | x | -0.043 | -0.091 | 0.043 |
| >5 | 0.361 | 0.369 | 0.363 | 0.315 | x | 0.613 | 0.229 | 0.652 |
| No. of previous fractures* | x | x | x | x | x | x | x | x |
| 0 | x | x | x | x | x | x | x | x |
| 1 | x | x | x | x | x | x | x | x |
| >=2 | x | x | x | x | x | x | x | x |
| Abbreviations: OST, patients with incident diagnosis of osteoporosis; IFX, patients with incident fragility fracture; OBP, incident users of oral bisphosphonates; MACE, composite outcome for the occurrence of either myocardial infarction, stroke or cardiovascular disease death; MI, myocardial infarction; * ever; ** in the year prior to start; SES, socio-economic status; BMI, body mass index; eGFR, estimated Glomerular Filtration Rate; SBP, cholesterol, systolic blood pressure; DBP, diastolic blood pressure. | | | | | | | | |

# Table S7c. Model equations from lasso selection for women and men based models (OBP cohort)

| OBP cohort  Predictor | Women Beta coefficients | | | | Men Beta coefficients | | | |
| --- | --- | --- | --- | --- | --- | --- | --- | --- |
|  | One year | | Two year | | One year | | Two year | |
|  | MACE | Stroke/MI | MACE | Stroke/MI | MACE | Stroke/MI | MACE | Stroke/MI |
| Intercept | -4.769 | -4.669 | -3.946 | -4.056 | -4.521 | -4.673 | -3.412 | -4.022 |
| SES |  |  |  | x | x | x |  | x |
| 1 | ref | ref | ref | x | x | x | ref | x |
| 2 | -0.006 | 0.131 | 0.070 | x | x | x | 0.106 | x |
| 3 | 0.164 | 0.291 | 0.223 | x | x | x | 0.087 | x |
| 4 | 0.129 | 0.230 | 0.177 | x | x | x | 0.228 | x |
| 5 | 0.240 | 0.216 | 0.181 | x | x | x | 0.099 | x |
| Smoking** | x |  |  |  | x |  | x | x |
| Ex | x | ref | ref | ref | x | ref | x | x |
| No | x | -0.184 | -0.128 | -0.099 | x | -0.025 | x | x |
| Yes | x | -0.090 | 0.085 | 0.206 | x | 0.154 | x | x |
| Drinking** |  |  |  |  | x | x |  | x |
| Ex | ref | ref | ref | ref | x | x | ref | x |
| No | -0.046 | 0.049 | -0.126 | -0.107 | x | x | -0.172 | x |
| Yes | -0.194 | -0.139 | -0.253 | -0.249 | x | x | -0.206 | x |
| Diabetes type I* | x | x | x | x | x | x | x | x |
| Diabetes type II* | 0.374 | 0.401 | 0.308 | x | x | x | x | x |
| Chronic obstructive pulmonary disease* | x | 0.234 | x | x | x | x | x | x |
| Chronic kidney disease* | -0.208 | x | -0.148 | x | x | x | x | x |
| Rheumatoid arthritis* | x | x | -0.004 | x | -0.569 | -0.317 | x | x |
| Lupus* | x | x | x | x | x | x | x | x |
| Systemic heart disease** | x | x | x | x | x | x | x | x |
| Anti-osteoporosis use** | x | x | x | x | x | x | x | x |
| Heparin use** | x | x | x | x | x | x | x | x |
| Beta-blocker use** | 0.073 | 0.225 | 0.175 | 0.154 | x | 0.044 | x | x |
| Hypertension** | x | x | x | x | x | x | x | x |
| Deep vein thrombosis or pulmonary embolism** | x | x | x | x | x | x | x | x |
| Anticoagulant use** | x | x | x | x | x | x | x | x |
| Antidepressants TCA** | x | x | x | x | x | x | x | x |
| Antidepressants SSRI** | x | 0.252 | 0.152 | x | x | x | x | x |
| Hypercholesterolemia** | x | x | x | x | x | x | x | x |
| Statin use** | x | -0.179 | x | x | x | x | x | x |
| Osteoporosis history* | -0.280 | -0.261 | -0.263 | -0.164 | -0.180 | x | -0.185 | -0.201 |
| Family history of cardiovascular disease | x | x | -0.013 | x | x | x | x | x |
| Family history of cardiovascular disease before age 60 | x | x | x | x | x | x | x | x |
| Heart failure* | x | x | x | x | x | 0.361 | x | x |
| Migraine* | x | x | x | x | x | x |  | x |
| Severe mental illness* | x | x | x | x | x | x | x | x |
| Vascular Disease* | x | x | x | x | x | x | x | x |
| Atrial fibrillation* | 0.448 | 0.369 | 0.418 | 0.336 | x | x | x | x |
| On anti-hypertensive drug | x | 0.130 | 0.089 | 0.160 | x | x | 0.131 | 0.101 |
| Antipsychotic use** | x | x | x | x | x | x | x | x |
| Steroid use** | x | 0.052 | x | x | x | -0.013 | x | x |
| Erectile dysfunction** | x | x | x | x | x | x | -0.112 | x |
| Age Group |  |  |  |  |  |  |  |  |
| 50-59 | ref | ref | ref | ref | ref | ref | ref | ref |
| 60-69 | 0.836 | 0.621 | 0.481 | 0.587 | 0.455 | 0.231 | 0.328 | 0.182 |
| 70-79 | 1.465 | 0.987 | 1.195 | 1.225 | 0.872 | 0.395 | 0.670 | 0.675 |
| 80-89 | 2.152 | 1.592 | 1.845 | 1.751 | 1.030 | 0.592 | 0.930 | 0.700 |
| >89 | 2.299 | 1.653 | 2.105 | 1.849 | 1.689 | 0.818 | 1.193 | 0.855 |
| Charlson score |  | x |  | x | x |  |  | x |
| 0 | ref | x | ref | x | x | ref | ref | x |
| 1 | -0.159 | x | -0.066 | x | x | 0.018 | -0.121 | x |
| 2 | -0.108 | x | -0.032 | x | x | 0.085 | -0.246 | x |
| ≥3 | -0.020 | x | -0.032 | x | x | 0.078 | -0.070 | x |
| Cardiovascular disease |  |  | x |  | x |  | x |  |
| No | ref | ref | x | ref | x | ref | x | ref |
| Ever >1 year before index date | 0.136 | 0.267 | x | 0.322 | x | 0.351 | x | 0.229 |
| 1 year before index | 0.106 | -0.019 | x | 0.083 | x | 0.605 | x | 0.159 |
| 6 months before index | 0.218 | 0.403 | x | 0.409 | x | 1.109 | x | 0.988 |
| 1 month before index | 0.764 | 1.042 | x | 0.759 | x | 1.298 | x | 0.920 |
| MI or Stroke |  |  |  |  | x |  |  | x |
| No | ref | ref | ref | ref | x | ref | ref | x |
| Ever >1 year before index date | 0.267 | 0.030 | 0.334 | 0.244 | x | -0.268 | 0.299 | x |
| 1 year before index | 0.739 | 0.765 | 0.684 | 0.494 | x | 0.634 | 0.846 | x |
| Established CVD * | 0.459 | 0.344 | 0.493 | 0.317 | 0.834 | 0.319 | 0.535 | 0.399 |
| Any fracture history | x | x | x | x | x | x | x | x |
| No | x | x | x | x | x | x | x | x |
| Ever >1 year before index date | x | x | x | x | x | x | x | x |
| 1 year before index | x | x | x | x | x | x | x | x |
| Hip fracture history | x | x | x | x | x | x |  | x |
| No | x | x | x | x | x | x | ref | x |
| Ever >1 year before index date | x | x | x | x | x | x | -0.107 | x |
| 1 year before index | x | x | x | x | x | x | 0.166 | x |
| Shoulder fracture history | x | x | x | x | x | x | x | x |
| No | x | x | x | x | x | x | x | x |
| Ever >1 year before index date | x | x | x | x | x | x | x | x |
| 1 year before index | x | x | x | x | x | x | x | x |
| Spine fracture history | x | x | x | x | x | x | x | x |
| No | x | x | x | x | x | x | x | x |
| Ever >1 year before index date | x | x | x | x | x | x | x | x |
| 1 year before index | x | x | x | x | x | x | x | x |
| Wrist fracture history | x | x | x | x | x | x | x | x |
| No | x | x | x | x | x | x | x | x |
| Ever >1 year before index date | x | x | x | x | x | x | x | x |
| 1 year before index | x | x | x | x | x | x | x | x |
| BMI** |  |  |  |  | x |  |  |  |
| <18.5 | ref | ref | ref | ref | x | ref | ref | ref |
| 18.6 - 24.9 | -0.328 | -0.249 | -0.290 | -0.234 | x | 0.100 | -0.185 | -0.087 |
| 25 - 29.9 | -0.553 | -0.389 | -0.549 | -0.404 | x | -0.136 | -0.330 | -0.198 |
| 30 - 39.9 | -0.609 | -0.529 | -0.540 | -0.389 | x | -0.252 | -0.558 | -0.272 |
| >=40 | -0.867 | -0.525 | -0.493 | -0.313 | x | -0.112 | -0.206 | -0.187 |
| No. of GP visits** |  |  | x |  | x |  |  |  |
| 0 | ref | ref | x | ref | x | ref | ref | ref |
| 1-5 | -0.046 | -0.159 | x | -0.093 | x | -0.658 | 0.061 | -0.029 |
| 6-10 | -0.175 | -0.137 | x | -0.180 | x | -0.575 | -0.126 | -0.376 |
| 11-15 | -0.364 | -0.233 | x | -0.266 | x | -0.594 | -0.118 | -0.287 |
| >=16 | -0.233 | -0.150 | x | -0.132 | x | -0.377 | 0.130 | -0.141 |
| No. of GP emergency visits** |  |  |  | x | x | x | x | x |
| 0 | ref | ref | ref | x | x | x | x | x |
| 1 | 0.086 | 0.085 | 0.157 | x | x | x | x | x |
| 2 | 0.281 | 0.310 | 0.288 | x | x | x | x | x |
| 3-5 | 0.332 | 0.087 | 0.177 | x | x | x | x | x |
| >=6 | 0.881 | 0.533 | 0.492 | x | x | x | x | x |
| eGFR** |  |  |  |  |  |  |  |  |
| <=29 | ref | ref | ref | ref | ref | ref | ref | ref |
| 30 – 44 | -0.118 | -0.060 | -0.069 | -0.132 | 0.355 | 0.190 | 0.320 | 0.451 |
| 45 – 59 | -0.221 | -0.224 | -0.195 | -0.276 | 0.253 | 0.255 | 0.154 | 0.270 |
| 60 – 89 | -0.585 | -0.550 | -0.445 | -0.508 | 0.049 | 0.050 | -0.008 | 0.092 |
| >=90 | -0.553 | -0.630 | -0.455 | -0.609 | -0.233 | -0.247 | -0.202 | -0.212 |
| SBP** |  |  |  |  |  |  |  |  |
| <120 | ref | ref | ref | ref | ref | ref | ref | ref |
| 120 - 139 | 0.015 | -0.063 | 0.129 | 0.061 | -0.058 | 0.290 | 0.033 | 0.260 |
| 140 - 159 | 0.062 | 0.047 | 0.166 | 0.134 | 0.174 | 0.566 | 0.291 | 0.440 |
| >=160 | 0.186 | 0.253 | 0.353 | 0.360 | 0.379 | 0.528 | 0.413 | 0.663 |
| DBP** |  |  |  | x | x | x | x | x |
| <80 | ref | ref | ref | x | x | x | x | x |
| 80 – 89 | -0.014 | -0.072 | -0.041 | x | x | x | x | x |
| 90 – 99 | 0.144 | 0.148 | -0.040 | x | x | x | x | x |
| >=100 | 0.431 | -0.003 | 0.304 | x | x | x | x | x |
| No. of concomitant medicines** |  |  |  |  | x |  |  |  |
| 0 | ref | ref | ref | ref | x | ref | ref | ref |
| 1 – 3 | -0.063 | -0.152 | -0.339 | -0.197 | x | 0.091 | -0.231 | -0.372 |
| 4 – 6 | -0.141 | -0.182 | -0.400 | -0.131 | x | -0.075 | -0.243 | -0.061 |
| 7 – 9 | 0.063 | -0.041 | -0.214 | -0.021 | x | 0.242 | -0.095 | 0.077 |
| 10 – 12 | 0.217 | -0.016 | -0.098 | 0.147 | x | 0.532 | -0.037 | 0.177 |
| >=13 | 0.247 | 0.106 | -0.126 | 0.193 | x | 0.510 | -0.018 | 0.285 |
| Cholesterol measurement** (HDL/LDL) |  |  |  | x | x |  | x | x |
| <=3.5 | ref | ref | ref | x | x | ref | x | x |
| 3.6 – 5 | 0.169 | 0.141 | 0.119 | x | x | 0.190 | x | x |
| >5 | 0.368 | 0.247 | 0.331 | x | x | 0.442 | x | x |
| No. of previous fractures* | x |  | x | x | x | x | x | x |
| 0 | x | ref | x | x | x | x | x | x |
| 1 | x | 0.089 | x | x | x | x | x | x |
| >=2 | x | 0.010 | x | x | x | x | x | x |
| Abbreviations: OST, patients with incident diagnosis of osteoporosis; IFX, patients with incident fragility fracture; OBP, incident users of oral bisphosphonates; MACE, composite outcome for the occurrence of either myocardial infarction, stroke or cardiovascular disease death; MI, myocardial infarction; * ever; ** in the year prior to start; SES, socio-economic status; BMI, body mass index; eGFR, estimated Glomerular Filtration Rate; SBP, cholesterol, systolic blood pressure; DBP, diastolic blood pressure. | | | | | | | | |
